# Ensemble molecular mimicry correlates with antibody cross-reactivity in proteome-wide studies

**DOI:** 10.3389/fimmu.2026.1749369

**Published:** 2026-02-10

**Authors:** James O. Wrabl, Josh Beale, Gabriel Fortunato, Antonieta van den Berg Monsalve, Vincent J. Hilser

**Affiliations:** 1 Department of Biology, Johns Hopkins University, Baltimore MD, United States; 2 Cell, Molecular, Developmental Biology, and Biophysics Graduate Program, Johns Hopkins University, Baltimore MD, United States; 3 T. C. Jenkins Department of Biophysics, Johns Hopkins University, Baltimore MD, United States; 4 Program in Molecular Biophysics Graduate Program, Johns Hopkins University, Baltimore MD, United States

**Keywords:** autoimmunity, binding energetics, conformational equilibrium, polyclonal, protein ensemble

## Abstract

Energetics of protein–protein binding necessarily include contributions both from conformational equilibria and from interfacial interactions. In the particular case of an antibody binding to a protein epitope, the conformational contribution is typically neglected as the antibody-bound and free forms of the protein are usually highly similar, leading to the reasonable conclusion that binding affinity in most cases can be reconciled in the context of observed interfacial interactions. However, the phenomenon of molecular mimicry has also been widely observed, wherein antibodies raised against one sequence/structure are able to recognize a completely different sequence/structure. This observation suggests that, in some cases, the conformational contribution could play a significant role in facilitating this cross-reactivity. Here, this conjecture is supported, utilizing a recent discovery that permits evaluation of the thermodynamic compatibility of any sequence for the conformational ensemble of any other protein—in effect providing direct access to the conformational contribution to binding. The importance of the contribution could then be assessed on a proteome-wide scale, in the context of the unexpected cross-reactivity observed when the human proteome is challenged with antibodies raised against a set of virus protein antigens. Because the virus protein antigens and the cross-reactive human proteins share substantial similarity when modeled as thermodynamic ensembles, despite the absence of detectable sequence or structural similarity, we hypothesize that these cross-reactive epitopes share a novel kind of immunological molecular mimicry, termed “ensemble molecular mimicry” (EMM). To investigate potential mechanisms, a sequence-based algorithm was developed to probe for the relationship between high scoring sequence segments and cross-reacting epitopes, and it was discovered that 9 of 11 medically relevant cross-reactive epitopes taken from the literature exhibited higher-than-expected local EMM values. Taken together, the results suggest that conformational equilibrium can affect affinity and that it is hypothetically possible for cross-reactive epitopes to share a pairwise thermodynamic signature, even in the absence of sequence or structural similarity.

## 1 Introduction

Thousands of high-resolution structures of antibody–antigen complexes exist in the Protein Data Bank (1, 2). This wealth of data has been transformative for the understanding of antibody specificity and computation of the intermolecular energetics underlying affinity. However, routine estimation of accurate binding energies from structure remains elusive and often requires resource-intensive simulations. As such, significant efforts have been directed towards force-field development and artificial intelligence-driven molecular modeling (3–7), but despite these efforts, reliable prediction of which antibodies will bind to which antigens, and how tightly, is not currently achievable (8).

While the binding energetics at the antibody–antigen interface are undoubtedly important and most accessible for study, less attention has been paid to the idea that every protein–protein binding reaction also includes a contribution from the ensemble conformational equilibrium. There is always a separate energy cost for the antigen (as well as the antibody) to adopt the specific conformations necessary to form the complex, i.e., a free energy of stability upon which the population of binding-competent antigen, as dictated by statistical thermodynamics, depends. In the case of high-affinity, monoclonal antibody complexes, this contribution might be safely ignored, as strong interfacial atomic interactions would be expected to dominate overall binding affinity. For weaker polyclonal responses, however, ensemble conformational equilibrium can be the decisive factor, as demonstrated by the elegant pioneering experiments of Anfinsen and colleagues (9, 10).

If ensemble conformational equilibrium is relevant to the polyclonal response to antigen, how can it be detected? In this work, we take a biophysical approach to the question by deploying state-of-the-art proteome chip technology, which enables large-scale measurements of relative binding affinities for a panel of polyclonal antibodies against nearly the entire human proteome (*N* ~ 20,000 proteins). These relative affinities are then compared to high-throughput computational estimates of ensemble conformational equilibrium developed in this laboratory, which are calculated in pairwise fashion between highest-affinity proteins from the chip and the antigens against which the polyclonal antibodies were raised.

In these experiments we test the null hypothesis that affinity and conformational equilibrium are unrelated, and based on modest but significant correlation between empirical affinities and computations, we conclude that ensemble conformational equilibrium should not be neglected as part of the polyclonal response. Furthermore, because the antigens are derived from a virus and the binding proteins come from the human proteome, the results report unexpected cross-reactivity and may help to explain documented cases of molecular mimicry in varied auto-immune diseases characterized by cross-reactive epitopes that do not exhibit obvious sequence or structure similarity (11–14). Instead, we hypothesize a new type of epitope based on “ensemble molecular mimicry” (EMM), which could be revealed through detailed thermodynamic analysis of local conformational equilibria between pairs of proteins. This ensemble information may be useful for the future prediction of auto-antigenic proteins and epitopes.

## 2 Results

### 2.1 Fluorescence as a proxy for total binding free energy

**Figure 1:**
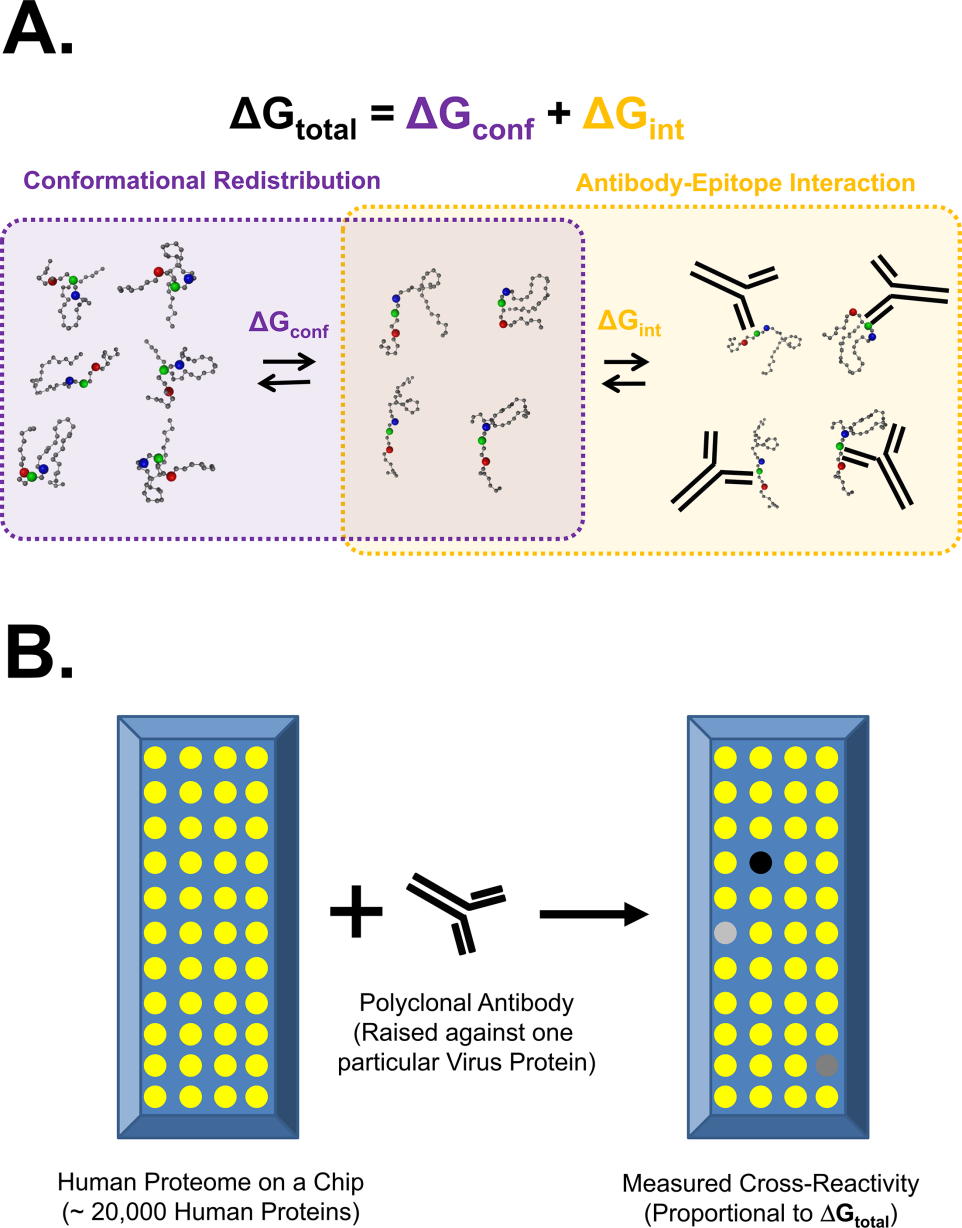
Conformational equilibrium contributes to binding affinity and affinity can be measured by proteome-on-a-chip technology. **(A)** Conformational equilibrium contributes to binding affinity. In general, coupled equilibria exist describing the binding between an antibody (black cartoon Y) and an antigen (colored beads-on-string cartoon). The free energy of binding (Δ*G*_total_) has two major contributions: a free energy of the antigen epitope interacting with the antibody paratope (Δ*G*_int_), and a free energy of the antigen epitope adopting the correct conformation to permit interaction (Δ*G*_conf_). For binding to occur (orange box, right side), the antigen must adopt a binding-competent conformation (purple box, right side) as opposed to a binding-incompetent conformation (purple box, left side). Conformational equilibrium of the epitope between binding-incompetent and binding-competent shapes is suggested by the purple box. The hypothesis tested in this work is that while epitope sequence conservation is reflected by Δ*G*_int_, thermodynamic molecular mimicry in the absence of sequence identity can be facilitated by a favorable Δ*G*_conf_. **(B)** Affinity can be measured by proteome-on-a-chip technology. Almost the entire human proteome can be expressed, purified, and spotted on a nitrocellulose/glass chip (left side) using technology developed by CDI Laboratories, Inc. (Baltimore, MD). Each yellow spot represents many copies of a localized particular human protein. An antibody or serum sample (black cartoon Y) can be passed over the chip, probing all of the proteome simultaneously with a primary antibody. Binding of primary antibody to localized proteins on the chip can be quantified with a fluorescent secondary antibody (not shown), akin to a Western blot. Schematized locations of binding are shown by dots with shades of gray. It is assumed that degree of fluorescence is proportional to relative affinity.

We consider any macromolecular binding reaction, such as that between an antibody (Ab) and a specific antigen, as a series of two coupled equilibria (**Figure 1A**) (9). In this scheme, binding free energy (Δ*G*_total_) is influenced by at least two energetic contributions: An *intrinsic* contribution (Δ*G*_int_) resulting from the interactions of particular chemical groups between the antibody and the antigen (such as hydrogen bonding, charge–charge interactions, and hydrophobic packing), and a conformational contribution (Δ*G*_conf_) reflecting the free energy required for the epitope to adopt a binding-competent geometry. We expect that the particular sequence of the amino acids at the binding interface is primarily accounted for by the Δ*G*_int_ term, whereas contributions associated with redistributing the conformational ensemble to those conformations capable of binding the antibody, e.g., protein folding, comprise the Δ*G*_conf_ term.

The classic view of antibody binding is one of rigid-body association, which assumes that the Δ*G*_conf_ term is relatively small and that tight binding originates from high structural compatibility between the Fab region of the antibody and the antigen that it binds. Indeed, high similarity is often observed when the structures of unbound and antibody-bound antigens are compared, supporting the notion that the conformational free energy difference between unbound and bound states is small, at least in those cases (Δ*G*_conf_ ~ 0) (15, 16). However, it is also well known that the antibody maturation process involves an initial polyclonal response, which consists of numerous sequence-distinct antibodies whose individual binding affinities are significantly lower than the mature (monoclonal) antibody, but whose collective binding affinity is relatively high. Moreover, the two contributors to overall binding energy may compensate for each other. For example, if an antigen readily populates a large sub-ensemble of conformational states, each of which can only provide sub-optimal contacts at the binding interface(s), then this “penalty” of a relatively high Δ*G*_int_ can be countered by a relatively low Δ*G*_conf_ (**Figure 1A**). In other words, the bound complex can be conformationally heterogeneous, as schematically depicted in **Figure 1A**.

Although the gold-standard measurement of binding affinity (Δ*G*_total_) requires bulk-solution biophysical methods that monitor direct binding, such as isothermal titration calorimetry, fluorescence anisotropy, or nuclear magnetic resonance, these low-throughput methods are not amenable to proteome-scale measurements of antibody–target complexes. To estimate Δ*G*_total_ on a large scale, we instead measure the secondary fluorescence signal generated when a primary antibody binds to a target protein localized on a nitrocellulose/glass chip, as implemented on *HuProt* chips by CDI Labs, Inc. (**Figure 1B**, Section 4.1). One *HuProt* chip can localize large numbers of folded proteins, including essentially the entire human proteome (**Figure 1B**), and the relative binding of an antibody sample probing the entire proteome can be quantified.

### 2.2 Thermodynamic similarity (eTFR) as a proxy for free energy of conformational equilibrium

The conformational equilibrium shown in **Figure 1A** is akin to a free energy of folding, which is notoriously difficult to measure by high-throughput methods [although progress is being made (17, 18)]. For this work, we continue the development of a computational model of the energetics of the protein ensemble (*COREX*), which recently has been shown to measure the relative energetic distance (Δ*G*_conf_) between two protein conformations (19–21). In direct support of the current work, we demonstrated in those studies that protein sequences sharing no similarity can nonetheless adopt the same conformation, and that this propensity is predictable based on our characterization of ensembles from numerous proteins. Here, we go further and ask, if a sequence has a propensity to adopt an alternative fold, can that protein also be recognized by a ligand (or antibody) that binds that alternative fold? To address this question, we modified our previously presented methodology, as described briefly below.

Our approach is termed *eScape* Thermodynamic Fold Recognition (*e*TFR). *e*TFR merges two previously published computational resources. The *eScape* (*e*nergy land*Scape*) algorithm is a sequence-based predictor of the local folding stability (Δ*G*) of a protein, along with the enthalpic (apolar and polar Δ*H*) and entropic (*T*Δ*S* of apolar and polar solvation, as well as conformational *T*Δ*S*) components of that stability (22, 23). This algorithm generates the energy landscape of the protein ensemble from information provided by *thermodynamics*, not amino acid sequence or structure (24–26). Specifically, the energy landscape identifies which regions of the protein are more or less locally stable (i.e., more or less likely to be populated in a locally structured conformation).

Thermodynamic Fold Recognition uses “thermodynamic environments” (27–29), defined by the clustering of a large database of vectors {Δ*G*, Δ*H*, *T*Δ*S*}, to create a protein’s “thermodynamic profile” (30, 31), which is used as a query to search a database of arbitrary amino acid sequences. Each pairwise alignment in the search is scored by dynamic programming subject to a thermodynamic substitution matrix, and the significance of each score is computed against a calibrated null model (19, 32). In short, *e*TFR uses a thermodynamic profile derived from *eScape* as a query to search a sequence database with a *thermodynamic* substitution matrix instead of an *amino acid* substitution matrix. Importantly, this procedure essentially quantifies the compatibility of a protein thermodynamic ensemble with an amino acid sequence, providing a potentially powerful alternative to traditional sequence–sequence, sequence–structure, or structure–structure algorithms, especially when the information output of the traditional methods is undetectably low.

Highest-significance *e*TFR matches are reliably obtained when the sequence of an extant protein is queried against its native thermodynamic profile, while next-highest matches often occur when *homologous sequences* are queried against the same profile (32). It has also been demonstrated that thermodynamic similarity can exist even below the “twilight zone” of sequence identity (31). It is thus likely that significant *e*TFR matches between two sequence-dissimilar proteins could predict a substantially favorable value of Δ*G*_conf_, indicating that the conformational ensembles of the two proteins overlap to some degree and that each protein (or parts thereof) may transiently populate similar local conformations to the other. If true, then antibodies raised against one protein (e.g., protein A) may bind to a second protein (e.g., protein B). This could happen because, as shown previously (19–21), high sequence compatibility corresponds to a low Δ*G*_conf_ for the sequence of protein B to adopt the structure of protein A. Importantly, these potential cross-reactivities could occur even in the absence of sequence or structural similarity.

We have demonstrated predictable antibody cross-reactivity on a small scale in at least three prospective cases of sequence-dissimilar pairs of proteins—one a member of the SARS-CoV-2 proteome and the other identified from the human proteome—which exhibit a high *e*TFR significance between them (**Supplementary Figure S1A**) and bind to the same antibody on Western blot (**Supplementary Figure S1B**). Of potential medical significance, these unexpected observations of cross-reactivity correspond to three of seven human proteins for which autoantibodies have been previously documented in the blood sera of acute COVID-19 patients (12, 13). We hypothesize that *e*TFR can report on the likelihood that two proteins, even ostensibly unrelated ones, share conformational ensemble similarity. In as much as the computed similarity has been shown to allow one protein to even adopt the fold of the second protein (19), without explicit consideration of structural complementarity, we were motivated to determine if those same principles applied to the thermodynamic signatures of epitopes, thus motivating the current proteome-wide study.

**Figure 2:**
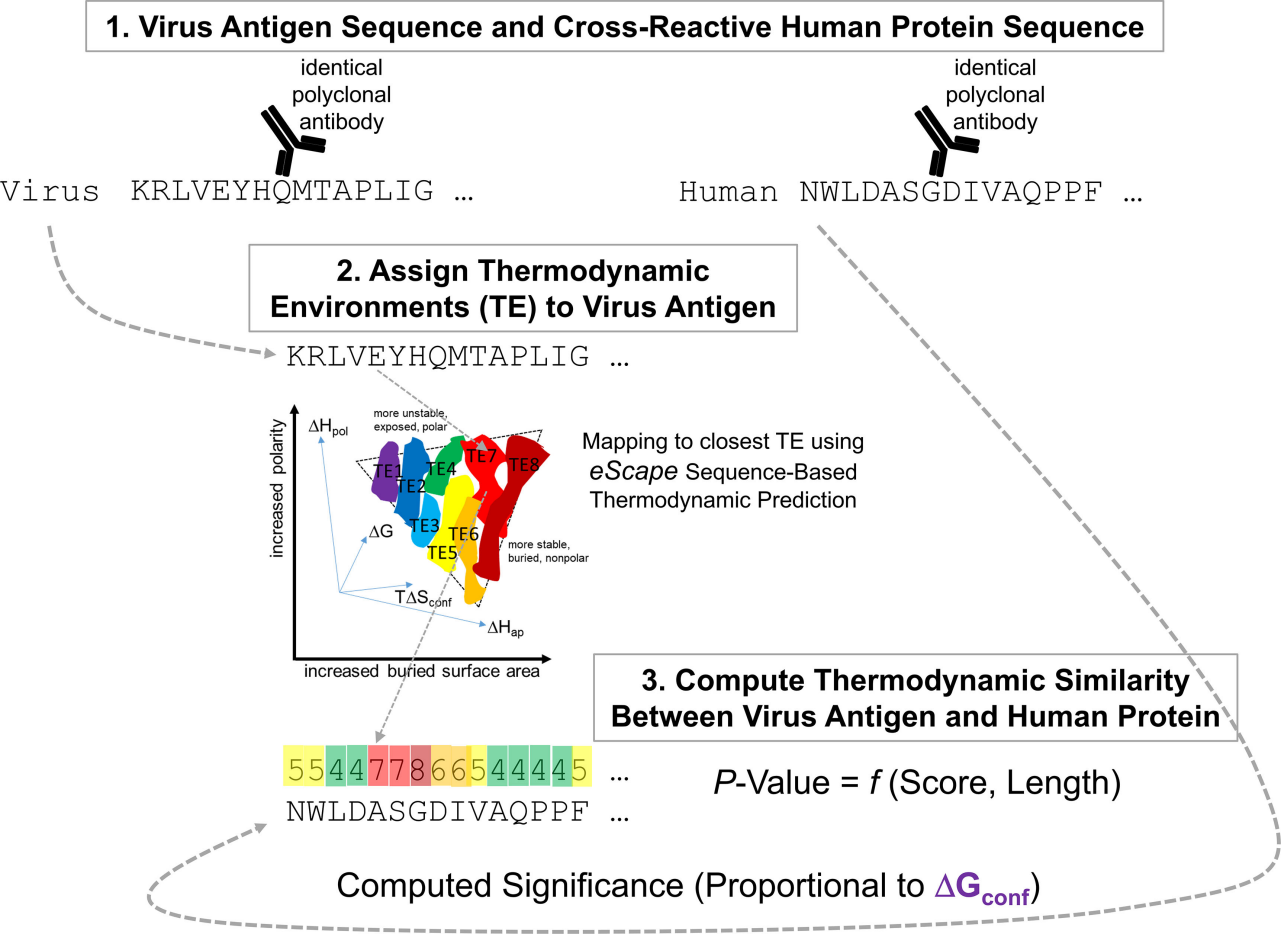
Workflow of Δ*G*_conf_ detection (ensemble molecular mimicry) using *e*TFR thermodynamic similarity. Two sequence-dissimilar epitopes (Box 1) exhibiting cross-reactivity to the same antibody (black cartoon Y) exist. The virus epitope (left) exhibits conformational equilibrium contributing to thermodynamic molecular mimicry with the human epitope, as depicted in **Figure 1A**. Fluctuations are encoded by thermodynamic environments (TE, Box 2), as described in Section 5.3. Briefly, the sequence-based prediction software, *eScape*, locates each residue of the full-length protein within a TE space spanning the local free energy, enthalpy, entropy, amino acid size, and polarity observed in globular proteins. TEs are assigned as an eight-letter numerical “alphabet” (colored boxes) permitting standard methods of alignment score optimization to a human host protein, subject to a TE-amino acid substitution matrix; this process is termed *eScape* Thermodynamic fold recognition (*e*TFR). Calibration of optimal score against a parameterized null model results in a significance value (*p*-value), which is taken as an estimation of Δ*G*_conf_, or thermodynamic molecular mimicry (Box 3). Thus, *e*TFR aligns two proteins with no explicit computation of amino acid sequence or structure similarity, instead using thermodynamic information, making it a suitable method for detection of low-sequence identity epitope similarities.

The workflow for *e*TFR matching is shown in **Figure 2**. Briefly, a thermodynamic profile of the virus antigen corresponding to a cognate polyclonal antibody (**Figure 2**, Box 1, left) was computed with *eScape* from its amino acid sequence. As alluded to above, *eScape* is a machine-learning algorithm that in the simplest case generates a four-dimensional vector of thermodynamic descriptors {Δ*G*, Δ*H*_ap_, Δ*H*_pol_, *T*Δ*S*_conf_} from each amino acid in a sequence, corresponding to the stability of the native (folded) state of the protein. Thermodynamic descriptors are mapped to thermodynamic environments (i.e., the closest cluster center of descriptors) observed in a large database of diverse proteins (**Figure 2** and Box 2). This reduces the four-dimensional thermodynamic descriptor space to a one-dimensional thermodynamic profile (**Figure 2** and Box 3, left, colored rectangles), for computational efficiency. Using standard dynamic programming, the thermodynamic profile can be optimally aligned to an arbitrary amino acid sequence (**Figure 2** and Box 3, left) and a significance can then be computed (**Figure 2** and Box 3, right). As mentioned above, in this work, the significance is considered a proxy for the Δ*G*_conf_ between the virus and human proteins used in the calculation (**Figure 2**, bottom).

### 2.3 Correlation between eTFR significance and chip fluorescence suggests association between ΔG_conf_ and ΔG_total_

**Figure 3:**
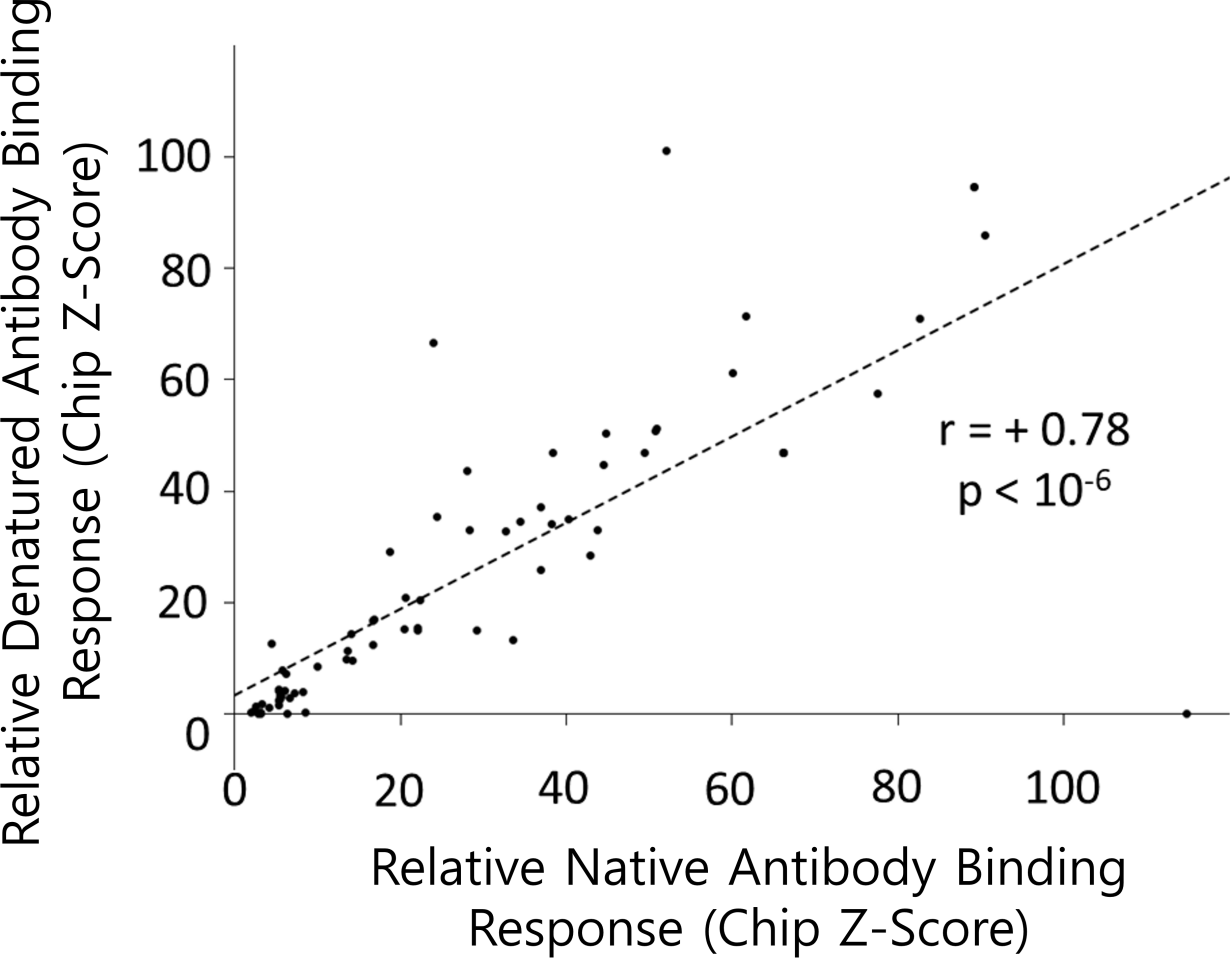
Cross-reactive epitopes are likely to be unstructured and continuous. Each point in the plot represents the fluorescence signal of one anti-virus antibody binding to the same human protein, measured under native conditions (*x*-axis) or denatured (8 M urea) conditions (*y*-axis). The strong correlation suggests that structured epitopes were not a general feature of any of the human proteins studied. There is one exception, namely, human NMD3 protein binding anti-SARS-Cov-2 nsp13 polyclonal, which suggests a signal for structured epitope(s). Data for these figures, and the proteins involved, are listed in **Supplementary Table S1**.

A panel of seven commercial polyclonal primary antibodies, raised against seven SARS-CoV-2 full-length protein antigens, were passed over separate human proteome chips, under both native and denaturing conditions (Sections 4.1 and 4.2). These antibodies were chosen from a preliminary scan of the thermodynamic profiles of the SARS-CoV-2 proteome against the amino acid sequences of all proteins in the Protein Data Bank (2) (Section 4.4, **Supplementary Figure S1A**). For each human protein spot on the chip, secondary antibody fluorescence proportional to primary antibody affinity was reported as a *Z*-score relative to all proteins on the chip, and the sum of native and denatured *Z*-scores for each human protein was calculated (sums were taken because fluorescence signals were found to be relatively independent of native or denatured conditions, cf. **Figure 3**).

The 10 strongest-binding human proteins against each of the seven anti-virus antibodies of the panel were prioritized as part of CDI Labs’ analysis report, so that 7 × 10 = 70 protein pairs of virus antigen and human protein were reported as cross-reactive in the company’s analysis (**Supplementary Table S1**). The *e*TFR significances computed for each virus antigen’s thermodynamic profile aligned to its paired human amino acid sequence was compared to the experimental binding affinity for the same pair. To explore the relationship between *e*TFR significance and binding affinity, the binding data from all seven chips were pooled, and the linear correlation between affinity and significance was directly calculated.

**Figure 4:**
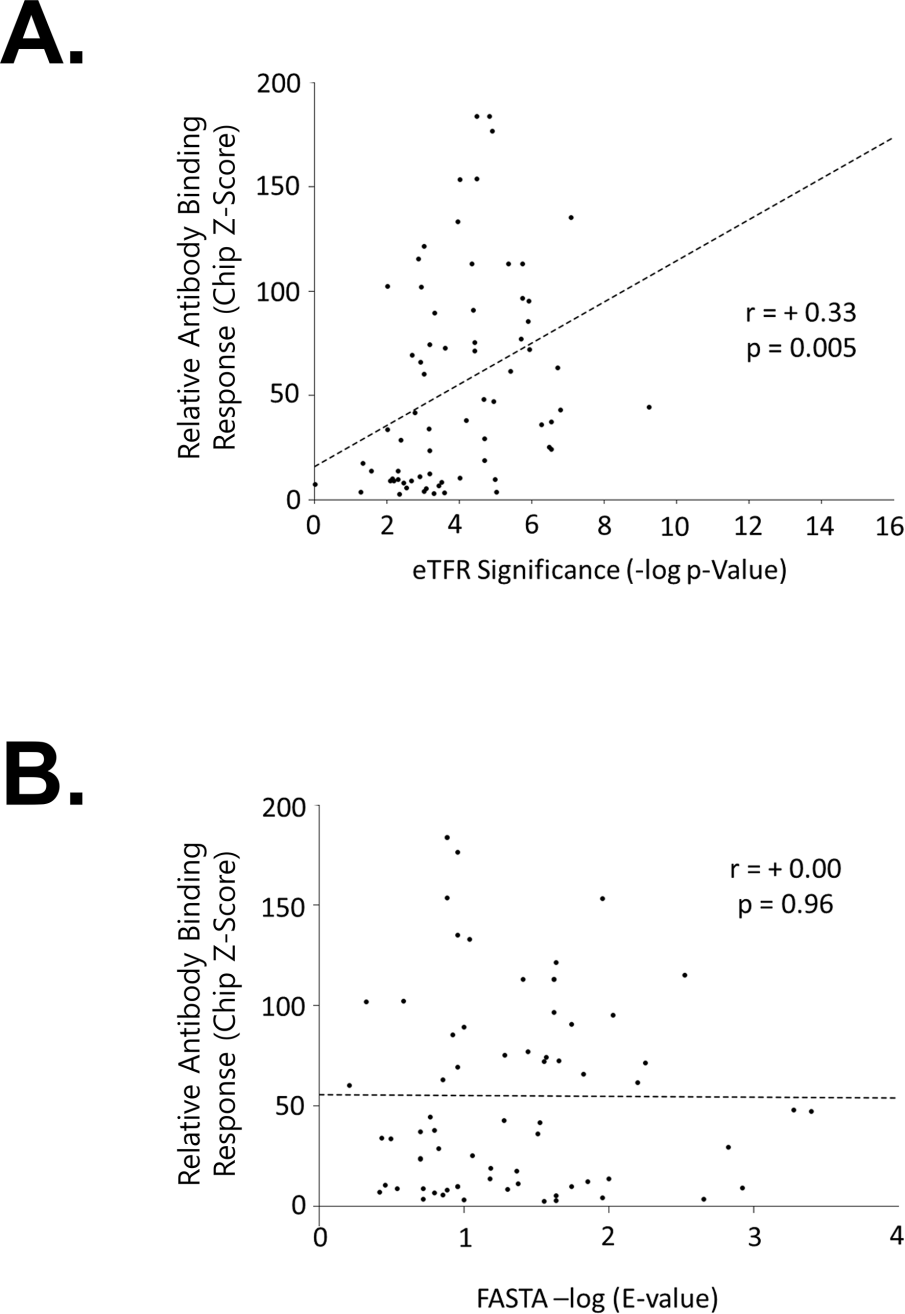
Sequence similarity does not contribute to Δ*G*_total_, but Δ*G*_conf_ does. **(A)** Significant relationship between chip *Z*-score and *e*TFR significance suggests that Δ*G*_conf_ contributes to Δ*G*_total_. Each point in the scatterplot represents one human protein compared to one virus protein. The *x*-axis denotes the computed thermodynamic similarity between the two proteins, which is related to Δ*G*_conf_, as described in the text (Section 2.2). The *y*-axis denotes the experimental fluorescence measured as the relative binding of the human protein to the anti-virus protein antibody, which is related to Δ*G*_total_, as described in the text (Section 2.1). A significant correlation suggests that Δ*G*_conf_ is associated with Δ*G*_total_. **(B)** Δ*G*_conf_ contributes to Δ*G*_total_ despite the absence of sequence similarity. Each point in the scatterplot represents one human protein compared to one virus protein. The *x*-axis plots the computed optimal local sequence similarity between the two proteins. The *y*-axis plots the experimental fluorescence measured as the human protein binds to the anti-virus protein antibody, which is related to Δ*G*_total_, as described in the text (Section 2.1). Lack of correlation suggests that sequence similarity is not associated with Δ*G*_total_. Data for these figures, and the proteins involved, are listed in **Supplementary Table S1**.

It might be expected that no relationship would exist between binding *Z*-score and *e*TFR significance—after all, the protein ensemble model *COREX*/*eScape* and the TFR/*e*TFR methods were not parameterized with any energetic information from protein–protein binding, nor were they trained with the structures of any antibody–antigen complexes—but indeed, a significant relationship (*p* = 5 × 10^−3^) was observed (**Figure 4A**). Insofar as *Z*-score and *e*TFR significance are related to Δ*G*_total_ and Δ*G*_conf_, respectively, this finding suggests that (1) Δ*G*_conf_ contributes to Δ*G*_total_, and (2) the magnitude of the contribution (i.e., the *R*^2^ value) is modest, approximately 10% for these cross-reactive protein pairs.

To rule out amino acid sequence identity as the driver for the correlation in **Figure 4A**, sequence similarity was computed for the 70 virus–human protein pairs by optimally aligning their full-length sequences using *FASTA36* (33) (Section 5.3). This local alignment algorithm is a stringent test for possible conserved sequence motifs as only the shortest, best match of highest sequence identity is reported, and random alignments are not considered. As no correlation was observed between *Z*-score and sequence similarity (**Figure 4B**), the possibility that amino acid identity represents the source of the correlation is not supported by these studies, which further suggests that the compatibility of a human protein sequence with a viral protein fold (i.e., a viral protein ensemble) is contributing to the overall binding affinity, Δ*G*_total._

## 3 Discussion

### 3.1 Thermodynamic similarity between full-length proteins and relation to epitopes

The importance of the finding that conformational compatibility between full-length sequences can contribute to antibody cross-reactivity cannot be overstated. Yet, identifying the underlying determinants in each case and establishing causality is challenging due to the nature of the information involved. To highlight these challenges, we first make a distinction between our previous findings and what we report here. In earlier work, we demonstrated that proteins, rather than being classified in static structural terms, could be represented in thermodynamic terms, which reflect the relative stabilities of the different parts of the protein in the functional ensemble (19). Importantly, we showed that globular proteins appear to share common patterns of amino acid composition within this thermodynamic framework, providing the basis for a statistical free energy calculation. This allowed us to evaluate the compatibility of any sequence with the thermodynamic environments of any other protein structure, regardless of sequence or structural similarity. Furthermore, the fact that (1) the score of a sequence for a fold was correlated with the empirical stability of that sequence in that fold (19), and (2) sequence mutations that increased the score for a different fold caused the sequence to switch folds (if the score were high enough) (19), directly demonstrates that the previously reported TFR scores provide a measure of thermodynamic compatibility (*i.e*., Δ*G*_conf_) of the two ensembles—in effect validating the notion that a new type of EMM is real and, to at least some degree, predictable in the context of full-length protein sequences.

The work presented here, on the other hand, investigates whether that same EMM plays a role in the well-known phenomenon of antibody cross-reactivity. We reasoned that for binding reactions wherein the Δ*G*_int_ term for antibody binding (cf. **Figure 1A**) is expected to be weak (as would be the case for the binding of proteins to antibodies raised against an entirely different protein), tight binding, if it were to be found at all, would be more likely to rely on a more favorable Δ*G*_conf_ term. Thus, challenging the human proteome with antibodies raised against seven viral proteins, which have no human homologs, provides a means for both identifying cases where cross-reactivity occurs, and determining the thermodynamic compatibility of the human sequence for the viral protein ensemble.

**Figure 5:**
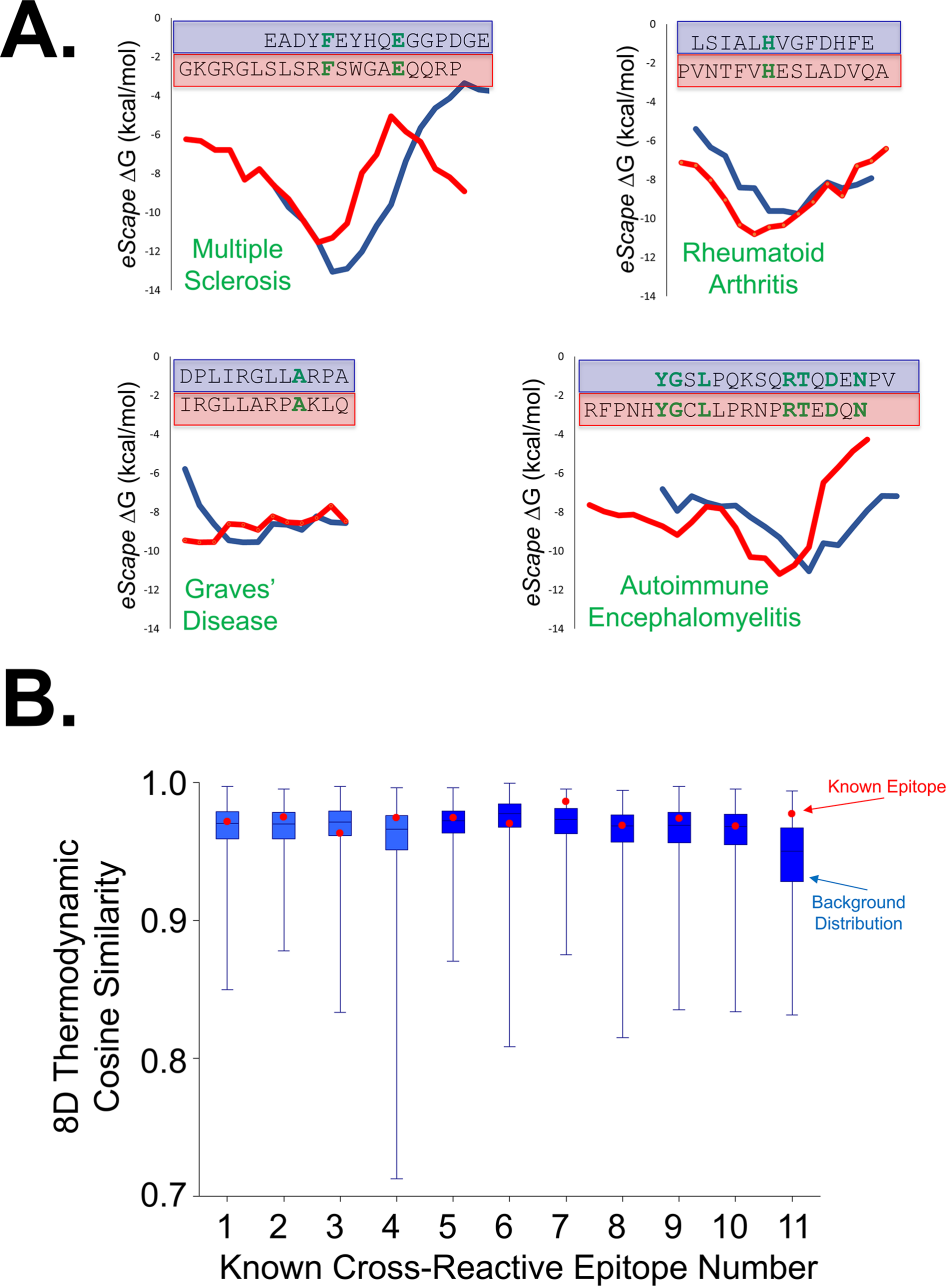
Known examples of medically relevant cross-reactive epitopes anecdotally share thermodynamic similarity in the absence of sequence similarity. **(A)** Four cases of known cross-reactive epitopes implicated in auto-immune disease molecular mimicry. For each example, an intriguing correspondence between the predicted *eScape* local stabilities of the published aligned epitopes are observed. Amino acid identities are highlighted in bold green font, emphasizing the lack of homology between the epitopes as the identities range from only 8% to 33%. Multiple sclerosis (38) (upper left): virus protein Epstein–Barr Virus Nuclear Antigen-1, residues 411–426 (blue), and human Myelin Basic Protein, residues 205–224 (red); Graves’ disease (37) (lower left): human thyroid peroxidase, residues 536–547 (blue), and human thyroid peroxidase, residues 539–550 (red); auto-immune encephalomyelitis (11) (lower right): human Myelin Basic Protein, residues 68–95 (blue), and Chlamydia cysteine-rich outer membrane protein peptide (CRP, red); rheumatoid arthritis (11) (upper right): Glucose-6-phosphate isomerase, residues 282–294 (blue), and Bovine RNase, residues 42–46 (red). **(B)***FVC* program indicates that known cross-reactive epitopes exhibit higher-than-average thermodynamic similarity. Box-and-whiskers plots were made using cosine similarity output from FVC, as described in Section 4.6. Error bars encompass the maximum and minimum cosine similarity scores of the background, the blue box encloses one-half of the background data, and the horizontal line within denotes the average. Cosine similarities for known epitopes (red points) are consistently higher than expected by the background distributions for the full-length proteins from which they are derived (blue). The significance of 9 out of 11 epitopes scoring higher than average is estimated to be *p* < 0.05. Numbers on the *x*-axis correspond to the following epitopes: (1) Multiple Sclerosis PLP and MHV proteins (11), (2) Multiple Sclerosis PLP and *H. influenzae* proteins (11), (3) Multiple Sclerosis MHV and *H. influenzae* proteins (11), (4) Encephalomyelitis MBP and *C. pneumoniae* proteins (11), (5) Myocarditis Myosin and CRP proteins (11), (6) Irritable Bowel Syndrome Mouse Hsp60 and Mycobacterium GroEL (11), (7) Rheumatoid Arthritis GPI and RNase (11), (8) Multiple Sclerosis Human MBP and Epstein–Barr Virus protein (11), (9) Liver autoimmunity Mouse FAH and MHV protein (46), (10) Multi-inflammatory syndrome in children Human SNX8 and SARS-CoV-2 orf9 (47), and (11) Multiple Sclerosis EBNA-1 and Human MBP (38). Numbers 4, 7, and 11 are also explicitly shown in Figure **(A)**.

Unfortunately, the total binding energy (i.e., Δ*G*_total_) for the cross-reacting antibodies consists of both Δ*G*_conf_, which is computed, and the individual Δ*G*_int_ terms, which will likely vary on a case-by-case basis and may involve other mechanisms. Thus, although the absence of sequence and structural similarity precludes other types of reported mimicry mechanisms playing a role [see (34) and discussion below], we cannot conclude that all of the cross-reactivity is due to the ensemble similarity. However, the fact that a modest but statistically significant (*p* = 0.005) correlation is observed (**Figure 4A**), even in the absence of recognizable sequence identity (**Figure 4B**), strongly supports the hypothesis that conformational free energy (Δ*G*_conf_) plays a role in antibody cross-reactivity across the proteome. The fact that the approach further reveals high thermodynamic scores for known cross-reactive epitopes (cf. **Figure 5** and discussion below), even though none of the proteins involved (or any antibodies) were included in the algorithmic development, demonstrates this point and highlights what we can conclude from these results and what we cannot. Succinctly put, the approach identifies what other protein sequences can sample the conformational ensemble of the antigenic protein, but it does not propose a specific mechanism for antibody cross-reactivity for each case. Instead, the results demonstrate that the full-length protein ensemble compatibility (i.e., mimicry) reported previously (19) and embodied in the Δ*G*_conf_ term in **Figure 1A** appears to contribute meaningfully to Δ*G*_total_, when viewed across the proteome, being especially evident when probing specifically for cross-reactivity. Perhaps the most important result is that this approach can provide investigators with the ability to determine which protein sequences have a significant compatibility with the ensemble of another protein (and thus can potentially cross-react with antibodies raised against that protein). This capability could be especially useful in detecting thermodynamic compatibility where no recognizable sequence or structural similarity exists.

Given the correlation between binding and conformational contribution reported here, and the caveats noted above, the questions, nonetheless, rightly turn to identifying the range of biophysical mechanisms for the unexpected cross-reactivity, and the location of the epitope(s). Is there a correlation between the epitope and the calculated high-scoring residues? Should there be? We note that unlike comparing experimental and calculated energies arising from the binding interface (35, 36), where the contribution of individual positions is explicitly computed, there is no obligatory position-specific correspondence; sequence determinants that result in high agreement of one sequence for an ensemble need not correspond to the amino acids that serve as the epitope for the actual binding part of the reaction (cf. **Figure 1A**). This point is made clear by considering that amino acids in stable, internal environments will contribute positively to high TFR scores, but epitopes are usually located primarily in less stable, surface-exposed sites (1, 15, 16). Consistent with this reasoning, it is noteworthy that our computational methods consider the full-length sequence of the antigen and cross-reacting proteins, and make no predictions about where the binding interfaces between antibody and antigen might be, as they are in principle unrelated. The situation is complicated further by the use of polyclonal antibodies, as, for example, the following two alternatives cannot be differentiated given an empirical binding affinity: (a) That many antibodies bind weakly to many epitopes on a given human protein, and the experimental signal is an integration of this heterogeneous response by the protein averaged over the ensemble, or (b) that few antibodies bind strongly to a single epitope on a given human protein, leading to the same experimental signal as (a).

The observed correlation between conformational equilibrium and relative antibody binding is also formally consistent with alternative models. For example, two or more distinct antibody populations within the polyclonal response could each bind unrelated epitopes on the virus and human proteins. In such a scenario, the association with ensemble similarity would still hold, but there would be no correspondence between the epitope and the scoring. These possibilities notwithstanding, identifying the location(s) of epitopes would help identify the range of scenarios and would further our understanding of this phenomenon. Distinguishing between these several possibilities positions techniques like epitope-mapping as the most straightforward way to address these questions, and studies to resolve these issues on a case-by-case basis are currently underway.

Given the challenges noted above, several lines of evidence support the notion that the computational methods may, nonetheless, provide clues about the nature and location of the epitopes in some cases. For example, fluorescence *Z*-scores obtained under denaturing vs. native conditions (**Figure 3**) suggest that the binding affinity is essentially independent of whether the human protein binder is folded or unfolded, implying that most epitopes are likely to be continuous. We further reasoned that modifying the full-length thermodynamic matching to a more localized thermodynamic matching (e.g., global or local sequence alignments in *FASTA* or *BLAST*) may provide candidates for continuous epitopes. Support for this hypothesis was found by anecdotal inspection of *eScape* Δ*G* values for several published sequence-dissimilar cross-reactive auto-immune epitopes (**Figure 5A**), which suggested that the local thermodynamic stabilities over 10- to 15-residue (i.e., epitope-sized) sequence fragments could exhibit similarity to the naked eye (although this similarity by itself does not rise to a level of statistical significance).

Armed with these observations, we developed an algorithm to extract optimal local matches from two *eScape* thermodynamic profiles, using only sequence information as input. The resultant *Fragment Vector Comparison* (*FVC*) algorithm calculates the *8xL*-dimensional cosine similarity between all pairwise sets of thermodynamic profiles of a specified window size, *L*, between two proteins (Section 4.6). Thus, a perfect correlation between the thermodynamics of two epitopes would yield an *FVC* score of 1.0, with lesser values indicating poorer matches. The resulting cosine similarities are then sorted, and the best local matches are retrieved.

(The *FVC* package, including *eScape*, is released along with this manuscript, and is freely available at the following site: https://github.com/jBeale23/FragmentVectorComparison).

**Figure 6:**
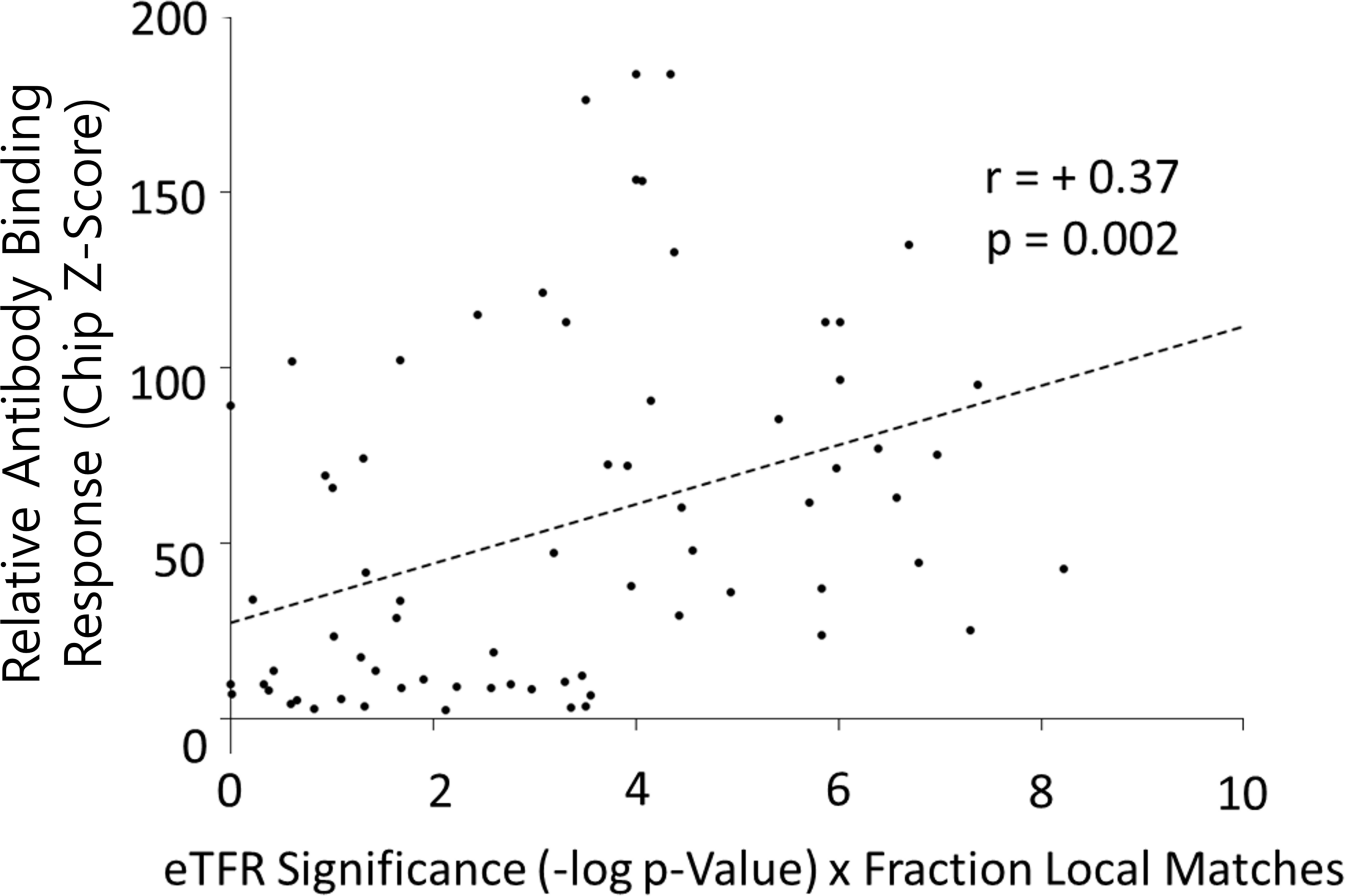
Simple weighting of *e*TFR significance by fraction of optimal local similarity improves correlation between Δ*G*_total_ and Δ*G*_conf_. Each point in the scatterplot represents one human protein compared to one virus protein. The *x*-axis plots the computed full-length thermodynamic similarity between the two proteins, which is related to Δ*G*_conf_, as described in the text (Section 2.2), weighted by the fraction of FVC matches greater than 0.99 contained within the full-length similarity. The *y*-axis plots the experimental fluorescence measured as the human protein binds to the anti-virus protein antibody, which is related to Δ*G*_total_, as described in the text (Section 2.1). A significant correlation suggests that Δ*G*_conf_ is associated with Δ*G*_total_. Because this correlation coefficient is improved over **Figure 4A**, this suggests that epitope-sized regions of strongest thermodynamic similarity are important contributors to the correlation (compare with **Figure 4A**).

Of note, when this approach was implemented, it was discovered that a majority of published cross-reactive epitopes from medical literature exhibited higher-than-average thermodynamic similarities (**Figure 5B**), a statistically significant result (*p* < 0.05). Furthermore, weighting each *e*TFR significance in **Figure 4A** by the fraction of the sequence that scores greater than 0.95 on *FVC* when matched with the viral protein measurably improved the correlation (**Figure 6**), as well as its significance (*p* = 2 × 10^−3^). This suggests that optimal local (epitope-sized) thermodynamic similarities, which may be subsumed within the optimal full-length similarities, may play an important role in the relationship between Δ*G*_conf_ and Δ*G*_total_, perhaps suggestive of a connection between Δ*G*_conf_ and Δ*G*_int_. Of course, the ultimate validity of this approach awaits additional experiments to determine whether *FVC* can actually predict the locations of the continuous cross-reactive epitopes responsible for **Figures 3**, **4A**. To support such experiments, a list of candidate *FVC* matches for each protein pair in **Figure 4** has been tabulated (**Supplementary Table S3**). It would truly be noteworthy if the determinants of the compatibility of a sequence for an ensemble overlapped with the epitope, but this is an open question, and one of the many possibilities requiring future studies.

### 3.2 Thermodynamic molecular mimicry: source of cross-reactivity?

One common property shared among all documented cross-reactive epitopes in **Figure 5** is that these medically relevant sequence fragments have undetectable pairwise identity with each other. How could one antibody bind to two epitopes in the absence of sequence similarity? Does a single subpopulation of the polyclonal antibody bind both the virus and human protein, or are there differences in the subpopulations that bind each protein? To explain this conundrum, the well-known phenomenon of molecular mimicry (34) has been proposed. Many examples of immunologic molecular mimicry have been documented (11, 37, 38), with Rojas et al. succinctly listing four distinct types spanning a continuum (34), ranging from complete structural similarity and sequence identity (Type 1) to structural similarity in the absence of sequence identity (Type 4). The type of molecular mimicry observed here is difficult to classify within this framework, because the common thermodynamic property is neither sequence nor structural in nature. These results suggest that the previously developed TFR scores in conjunction with the newly developed *FVC* matches provide a useful means of investigating the relationship between a more local Δ*G*_conf_ and the Δ*G*_int_ associated with the cross-reactive epitopes. Progress on these questions await epitope mapping studies on numerous systems.

**Figure 7:**
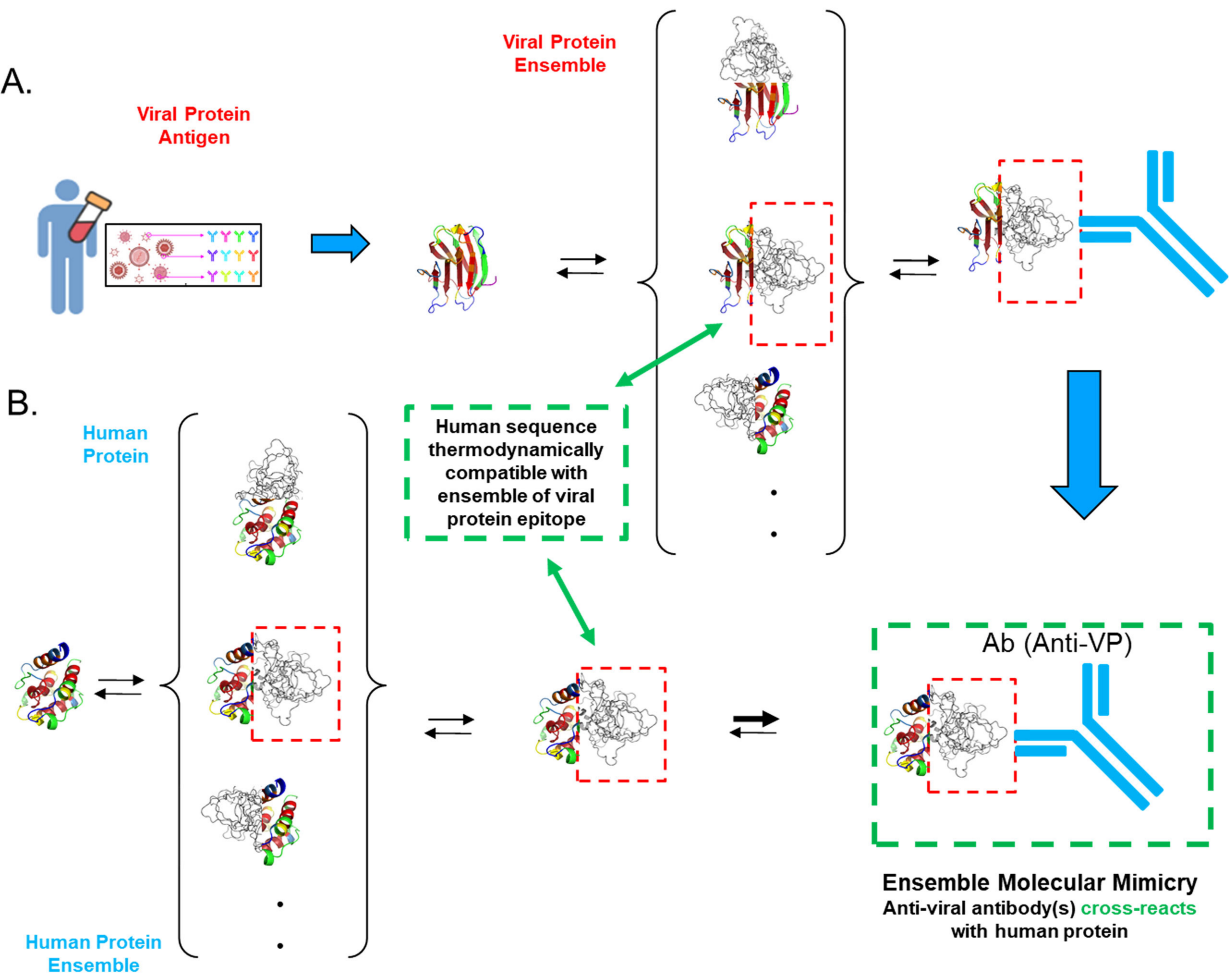
Ensemble molecular mimicry. **(A)** A viral protein elicits a polyclonal immune response wherein the antibodies recognize specific antigenic sequence elements in the viral protein (red box, dashed border). **(B)** For any given protein (in this case human), there exists a conformational ensemble involving, among other states, those conformations that the antigenic viral sequence adopts (red box, dashed border). Human sequences that are compatible with the viral protein fold (green box, dashed border) will have increased probability of cross-reacting with the corresponding anti-viral antibody.

The most general physical interpretation of EMM is that antibodies do not necessarily bind to a unique state, but instead to a subset of thermodynamically similar conformational states within an ensemble (**Figure 1A**). Pairs of sequences can, to different extents, populate states that are thermodynamically similar to each other; thus, our hypothesis is that in such cases, antibodies raised to only one sequence can nonetheless bind both, as shown in the schematic model of EMM depicted in **Figure 7**. Importantly, even in the absence of detectable structural similarity in the aligned regions, chemical groups such as hydrophobic side chains or hydrogen bond donors/acceptors would be geometrically placed to permit the same constellation of antibody(ies) to bind both antigens (37). This is the same principle that allows us to evaluate the preference of an amino acid sequence to adopt one conformation over another simply based on the scoring of amino acids in the corresponding environments of the new conformation without explicit consideration of structural context or interactions (19).

Finally, with regard to medical relevance, the phenomenon of EMM identified here may play a role in unexplained aspects of auto-immunity, such as promiscuous antibodies (34, 39, 40) or the breaking of immune tolerance in the absence of sequence similarity between self and non-self (41, 42). However, almost all extant cases of mimicry have been discovered individually in resource-intensive experiments (11, 37, 38, 43), motivating the need for cross-reactivity-prediction tools that are less reliant on amino acid sequence identity. It is our hope that the observations reported here, as well as the tools accompanying this manuscript, will provide a new avenue for addressing this need and identifying possible molecular mimicry within a proteome.

## 4 Methods

### 4.1 Human proteome-on-a-chip experiments

Rabbit polyclonal IgG primary antibodies raised against purified complete SARS-CoV-2 proteins were purchased off-the-shelf from commercial sources, as described below. Primary antibodies listed below, and in **Supplementary Table S1**, were sent to CDI Laboratories (Baltimore, MD and Mayaguez, PR) and passed over separate *HuProt* chips containing essentially complete representatives of each protein in the human proteome (*HuProt v4.0*, 21,215 distinct amino acid sequences). Binding of antibody to individual proteins was assessed by fluorescence (Alexa 555) of secondary anti-rabbit IgG according to the established procedure of CDI Laboratories. Binding was quantified for each human protein with a *Z*-score, representing the number of standard deviations above or below the average fluorescence signal over the entire chip. Objective analysis reports were received from CDI Laboratories listing the top 10 highest binding human proteins on each chip (**Supplementary Table S1**).

With the exception of the anti-SARS-CoV2-orf10 antibody, two chips were run for each antibody: a “native” chip, where the proteins on the chip were never exposed to denaturant, and a “denatured” chip, where the proteins on the chip were exposed to 8 M urea before incubation with the primary antibody. Native and denatured chips were compared to assess the possibility of structured epitopes, and it was found that there was generally a high correlation between native and denatured binding measurements (**Figure 3**). This suggested the lack of widespread structured epitopes, indicating largely redundant information between native and denatured (**Supplementary Table S1**). A technical limitation resulted in the unavailability of denatured binding data for the orf10 experiment. Because of the high correlation between native and denatured observed for the other samples, orf10 native state values were substituted where necessary, without loss of information. However, it is emphasized that this substitution introduces a small but non-negligible source of uncertainty.

Although *HuProt* assays are reported to be reasonably reproducible, we explicitly tested the reproducibility of a separate antibody sample on chips from different batches, 1 year apart. This was polyclonal anti-human CD53 (Bioss Antibodies bs-13625R). The results indicated high correlation between the binding affinities of the two chips under native conditions (**Supplementary Figure S2**). Therefore, the measurements and conclusions are believed to be robust.

### 4.2 Polyclonal antibodies used

All primary antibodies used here were polyclonal Rabbit IgG from commercial sources, at stock concentrations of 0.1–1.0 mg/mL and generally raised against the full-length protein. Manufacturers included anti-SARS-CoV-2-orf9 (Novus Biologicals NBP3-00510), anti-SARS-CoV-2-nsp13 (Life Technologies/Invitrogen PA5120711), anti-SARS-CoV-2-nsp16 (Life Technologies/Invitrogen PA5120701), anti-SARS-CoV-2-Spike (Life Technologies/Invitrogen PA5116916), anti-SARS-CoV-2-orf6 (Life Technologies/Invitrogen PA5120715), anti-SARS-CoV-2-orf8 (Antibodies-Online ABIN7383791), and anti-SARS-CoV-2-orf10 (Antibodies-Online ABIN6952939). These antibodies were chosen based on a preliminary *e*TFR scan of the SARS-CoV-2 proteome against the amino acid sequences of the Protein Data Bank (2) described below and in **Supplementary Figure S1A**.

### 4.3 Computational methods: eTFR and FASTA alignments

The *e*TFR thermodynamic alignment algorithm was executed as described in previous publications, without modification. In detail, the process was as follows: One full-length virus protein sequence was fed into the *eScape* algorithm as input (included within the *FVC* code released with this manuscript, web app at *best.bio.jhu.edu/eScape*) (22, 23). The output, two sets of four-dimensional vectors {Δ*G*, Δ*H*_ap_, Δ*H*_pol_, *T*Δ*S*_conf_} corresponding to the native state and denatured state thermodynamic descriptors for each residue of the protein, were mapped to the eight native state and eight denatured state thermodynamic environments, as previously described (44). This information constitutes the complete query thermodynamic profile, and the process was repeated for each virus protein (i.e., the antigen against which each polyclonal was raised).

The workflow proceeded as individual pairwise gapless alignments of each query with the 10 amino acid sequences prioritized by CDI Labs, comparing the shorter of the pair in all possible registers with the longer sequence (32). Each register was scored by consulting a thermodynamic substitution matrix for each aligned amino acid–environment pair and summing the total over the entire alignment (32). The total score was converted into a significance (*p*-value) according to the null model and length-dependent equation, as described previously (32). Separate scores and significances were computed for the native state and the denatured state, and the summed negative logs of the native and denatured significances were taken as the total score for that register (19). Finally, the maximum over all registers was taken as the optimal alignment and significance for that thermodynamic query–amino acid sequence pair. (These values are plotted on the *x*-axis of **Figure 4** and are listed in **Supplementary Table S1**.) Thus, for the seven proteins in the SARS-CoV-2 proteome considered, the *e*TFR procedure resulted in two outputs (1): a pairwise alignment between the virus amino acid sequence and the human sequence (**Supplementary Table S2**), and (2) a significance estimation for that alignment (**Supplementary Table S1**).

Amino acid sequence alignments were performed using the FASTA36 package (33) and BLOSUM62 substitution matrix (45), with all default settings. The most significant *E*-value was always taken as the single optimal result between two pairs of proteins; these values are plotted on the *x*-axis of **Figure 4B**.

### 4.4 Preliminary eTFR scan of the Protein Data Bank

Thermodynamic profiles of the Wuhan-Hu-1 SARS-CoV-2 proteome were computed using *eScape* from the 28 amino acid sequences translated from the NCBI Genomes accession NC_045512.2 (ncbi.nlm.nih.gov). Each of the 28 proteins in the SARS-CoV-2 proteome was used separately as query to exhaustively match all 594,420 amino acid sequences in the Protein Data Bank [download version date 06/14/21, *rcsb.org* (2)]. In detail, the process was as follows: One virus protein was fed into the *eScape* algorithm as input (included with the *FVC* code released with this manuscript, or web app at *best.bio.jhu.edu/eScape*). The output, two sets of four-dimensional vectors {Δ*G*, Δ*H*_ap_, Δ*H*_pol_, *T*Δ*S*_conf_} corresponding to the native state and denatured state thermodynamic descriptors for each residue of the protein, were mapped to the eight native state and eight denatured state thermodynamic environments, as previously described (44). This information constitutes the complete query thermodynamic profile, and the process was repeated for each virus protein.

The query was then used to exhaustively search a database of all amino acid sequences of known structure, as contained in the Protein Data Bank. The search proceeded as individual pairwise gapless alignments of the query with one amino acid sequence, comparing the shorter of the pair in all possible registers with the longer one (32). Each register was scored by consulting a thermodynamic substitution matrix for each aligned amino acid–environment pair and summing the total over the entire alignment (32). The total score was converted into a significance (*p*-value) according to the null model and length-dependent equation, as described previously (32). Separate scores and significances were computed for the native state and the denatured state, and the summed negative logs of the native and denatured-state significances were taken as the total score for that register (19). Finally, the maximum over all registers was taken as the optimal alignment and significance for that thermodynamic query–amino acid sequence pair. These significance values are plotted on the *y*-axis of **Supplementary Figure S1A**.

The highest-scoring human proteins against each viral protein thermodynamic profile were tabulated by manual inspection (these proteins are indicated by red bars in **Supplementary Figure S1A**). Seven virus–human matches were subjectively chosen on the basis of commercial availability of polyclonal antibody raised against the full-length viral protein and commercial availability of purified full-length human protein for Western blot analysis, described in the following. These seven matches chosen for testing are circled and numbered in **Supplementary Figure S1A**. The seven polyclonal antibodies comprised the panel of reagents used for the proteome-on-a-chip experiments shown in **Figure 4** and **Supplementary Table S1**.

### 4.5 Western blot experiments confirming cross-reactivity

Purified protein was obtained from commercial sources, as described below, at concentrations of approximately 0.1–1.0 mg/mL. Generally, 10–20 μL of this protein was loaded into 12%–20% SDS-polyacrylamide Laemmli reducing slab gels to completion at ambient temperature (1 h, 200 V). Electrophoretic transfer to nitrocellulose membrane from the gel was performed under Tris–glycine–methanol pH 8.3 buffer conditions to completion at 4 °C on ice (2 h, 400 A constant current). The membrane was blocked with Blocking Buffer (137 mM NaCl, 20 mM Tris, 0.1% Tween-20, and 5% dry milk, pH 7.6) for 24–48 h at 4°C. Then, the membrane was incubated with the primary antibody from the list above for at least 1 h at room temperature, washed 3× with TBST (137 mM NaCl, 20 mM Tris, and 0.1% Tween-20, pH 7.6), incubated at least 30 min at room temperature with anti-rabbit IgG secondary antibody linked to horseradish peroxidase, and washed 3× with TBST. Chemo-luminescence was generated with no more than 24-h-old SignalFire reagents (Cell Signaling Technology, Danvers, MA), according to the manufacturer’s directions. Imaging was performed with CL-XPosure film (ThermoFisher Scientific, Baltimore, MD) at a short exposure of 30 s, and again at a long exposure of 2 h. Imaging was also performed in separate experiments with fluorescence scanning after incubation with DyLite 800 PEG 4× conjugate secondary antibody, according to the manufacturer’s directions (Cell Signaling Technology, Danvers, MA). Images are displayed in **Supplementary Figure S1B**. All cross-reactivity results shown in **Supplementary Figure S1B** were reproducible at least three times.

Primary antibody solutions were generally diluted 1:1,000 in Blocking Buffer, assuming an antibody concentration of approximately 0.1 mg/mL. Secondary antibodies were generally diluted 1:1,000 in Blocking Buffer, assuming an antibody concentration of approximately 0.1 mg/mL. Primary and secondary antibodies were reused for periods of up to 2 weeks, with storage at 4 °C and added 0.1% final concentration sodium azide during the intervals.

All proteins used were commercially available at stock concentrations of 0.1–1.0 mg/mL and were full length (or the longest length available, subject to coverage of the region of computational prediction). We purchased expected antigens as well as predicted cross-reactive proteins to test the efficacy of the polyclonal antibodies: all polyclonals were judged effective by Western blot analysis (data not shown). Manufacturers from which antigens and proteins were obtained were as follows: SARS-CoV-2-orf9 (R&D Systems/Bio-techne 11033-CV-100), SARS-CoV-2-nsp13 (ProSci 10-427), SARS-CoV-2-nsp16 (ProSci 20-243, N-term uncleaved GST fusion), SARS-CoV-2-Spike (Life Technologies/Invitrogen RP87671, aa16-1213), SARS-CoV-2-orf6 (ProSci 20-193, N-term uncleaved MBP), SARS-CoV-2-orf8 (Life Technologies/Invitrogen RP87666, aa16-121), SARS-CoV-2-orf10 (ProSci 20-189, N-term uncleaved MBP), human-IL11 (Life Technologies/Gibco PHC0115), human-IGLL5 (MyBioSource MBS1029820), human-IL6 (Life Technologies/Invitrogen RP8619), human-SCN4B (OriGene TP323951), human-KDR/VEGFR2 (OriGene TP710248, aa20-764), human-CXCR4 (Abnova H00007852-G01), and human-CD53 (Abnova H00000963-G01).

### 4.6 Fragment vector comparison

The python implementation of the *FVC* program was used with all default settings, except that similarity cutoff was set to zero to record all local pairwise comparisons of fragment length *L* between two amino acid sequences. *L* was set to values between 9 and 25 residues long, depending on the length of the published alignment between one pair of cross-reactive epitopes. For each pair of cross-reactive epitopes, all cosine similarity scores computed for length *L* were used as the background distribution to make the box-and-whiskers plots in **Figure 5B**. The single score corresponding to the fragment of length *L* in the register of the published alignment (i.e., only the documented epitope) was used to plot the red dots in **Figure 5B**. The source code of *FVC*, which includes the *eScape* algorithm, is released as part of this manuscript, including a C implementation of the cosine similarity calculation that allows for higher throughput on supported device architectures. The code is freely and publicly available at the following site: https://github.com/jBeale23/FragmentVectorComparison.

For **Figure 6**, *FVC* was run separately on each of the 70 virus antigen–human protein pairs with window size set to 20 and similarity cutoff set to zero. Then, for each virus–human pair, only *FVC* scores corresponding to the overlapping windows of the register found in the full-length thermodynamic *e*TFR alignment were retained. Of these *N* scores, a weight *f* was computed from *f* = *M*/*N*, where *M* was the number of scores with cosine similarity greater than 0.95. The *x*-axis of **Figure 6** was thus computed from *S* × *f*, where *S* is the *e*TFR significance (−log *p*-value) from **Figure 4A**. Values for *f*, *M*, and *N* obtained are listed in **Supplementary Table S1**. This procedure merely weighted the full-length significance by the fraction of “very similar” local thermodynamic matches within the full-length alignment.

To facilitate future experiments, a list of the three highest matches of 20 residues found by *FVC* in the context of the full-length *e*TFR alignment between each pair of proteins in **Figure 4** is given in **Supplementary Table S3**. Note that these data may also be used to independently check the proper working of a locally installed version of *FVC*.

## Data Availability

The datasets presented in this study can be found in the **Supplementary Material**.
