# Supplementary material for: Ensemble molecular mimicry correlates with antibody cross-reactivity in proteome-wide studies
Source: Front Immunol. 2026 Feb 10;17:1749369. doi: 10.3389/fimmu.2026.1749369 (PMC12929536; doi:10.3389/fimmu.2026.1749369)
Supplement: Supplementary file 1 [file DataSheet1.pdf]

Supplementary Figures and Table Legends

Figure S1. Selection and preliminary testing of cross-reactive proteins and antibodies. A. An *e*TFR scan of the SARS-CoV-2 proteome against the amino acid sequences contained in the Protein Data Bank prioritizes experimentally cross-reactive pairs of proteins and antibodies. The x-axis displays the sequential proteome of the SARS-CoV-2 virus, enumerated as nucleotide position in the genome. Individual proteins are labeled (*e.g.* nsp1, nsp2, etc.). The y-axis displays the *e*TFR significance of the match between the thermodynamic profile of the virus protein and the amino acid sequence of every protein contained in the Protein Data Bank (expressed as –log p-value, thus a higher y-value indicates a better match). Matches are shown as horizontal bars in shades of gray, according to the thermodynamically aligned regions of the two proteins. Blue bars at the top of the plot are known SARS-CoV-2 proteins or homologs (*e.g*. other SARS or MERS proteins), which suggests that *e*TFR is effective at detecting expected protein similarities. Red bars with moderately high *e*TFR matches in the range of 8-12 indicate the highest-scoring human proteins contained within the Protein Data Bank for each of the SARS-CoV-2 proteins. These scores are "moderately good" matches compared to empirical values for random *e*TFR matches, or matches with substantial secondary structure similarity but low tertiary structure similarity (labeled dotted lines, calibrated as described within White, *et al.* 2021 *Biochemistry* **60**:1647). Circled and numbered human proteins indicate matches taken forward for experimental testing on the basis of commercial reagent availability, as described in Methods Section 4.2 and 4.5. These human proteins are: 1. 6qb6L (IGLC2/IGLL5), 2. 1p9mB (IL6), 3. 7dtdB (SCN4B), 4. 2m59A (VEGFR2), 5. 3oe9A (CXCR4), 6. 4mhlA (IL11), 7. 6wvgA (CD53). **B. Western Blots confirm that at least three high-scoring *e*TFR matches are cross-reactive.** For cases 1, 3, and 7 described in Figure S1A, polyclonal antibodies raised against the virus protein react with both the virus protein and the high-scoring human protein discovered in the *e*TFR scan.

Figure S2. Significant correlation between binding data from two different chips probed by the same antibody. The anti-CD53 antibody, as detailed in Main Text Methods Section 4.1, was used to probe different human proteome chips from different production lots, one year apart. Strong correlation suggests results of binding under native conditions is reproducible.

Table S1. Top Ten Native and Denatured Chip Z-Scores for each polyclonal antibody, and *e*TFR values for the virus:human optimal thermodynamic alignments. Data used for scatterplots in Main Text Figures 3, 4, and 6. Protein names and chip identifiers used by CDI Laboratories are displayed in columns 4 and 5.

Table S2. Optimal Thermodynamic Alignments from Supplementary Table S1, expressed as pairwise sequence alignments. The aligned regions of each pair of proteins are displayed in one box. Index corresponds to Supplementary Table S1. Protein names and chip identifiers used by CDI Laboratories are appended after the C-terminus of each aligned region.

Table S3. Optimal Thermodynamic Alignments from Supplementary Table S1, expressed as pairwise sequence alignments, with three highest non-overlapping FVC matches labeled. Highlighted colors correspond to the first (red), second (yellow), and third (blue) best FVC matches, corresponding to locations of potential cross-reactivity with the strongest conformational equilibrium contributions, in the context of the *e*TFR alignment between the two full-length proteins. The aligned regions of each pair of

proteins are displayed in one box, like Supplementary Table S2. Index corresponds to Supplementary Table S1. Protein names and chip identifiers used by CDI Laboratories are appended after the C-terminus of each aligned region. Numbers in braces represent the best, second, and third highest cosine similarities returned by the FVC program, corresponding to red, yellow, and blue matches respectively. (These numbers could be used to check if the FVC program works for a specific local installation – the command used to generate each FVC result is, *e.g.* for the Index 1 example ' FVC –similarityThreshold=0 –windowSize=20 orf9.fasta NMD3.fasta', where orf9.fasta and NMD3.fasta contain the amino acid sequences listed in the Index 1 row.) Because of its short sequence length, SARS-CoV-2 protein orf10 cannot accommodate more than one non-overlapping 20 –residue region.

Supplementary Figure S1.

A.

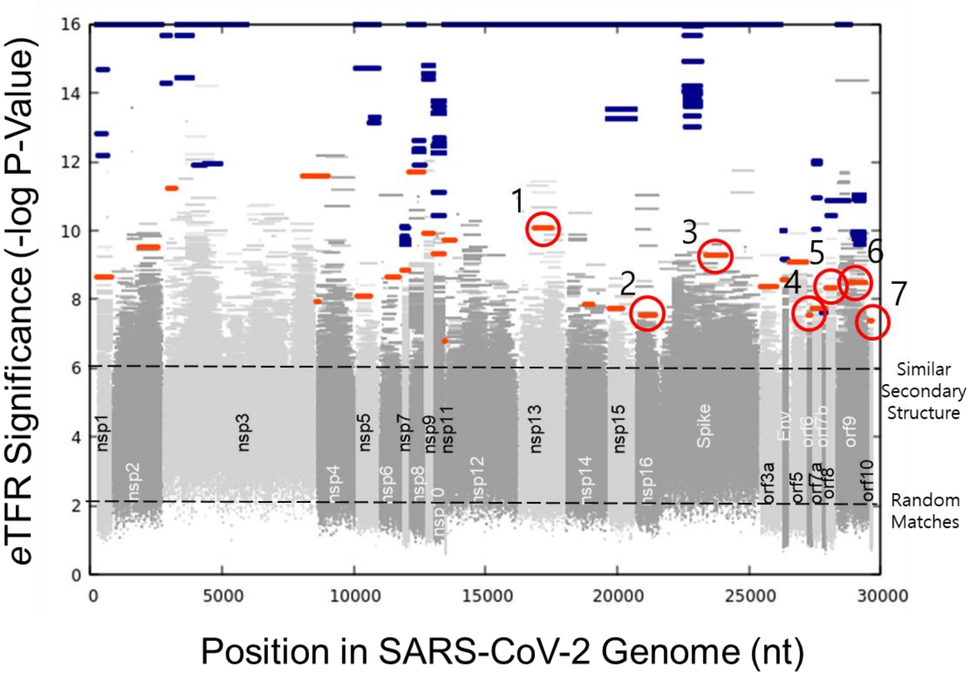

B.

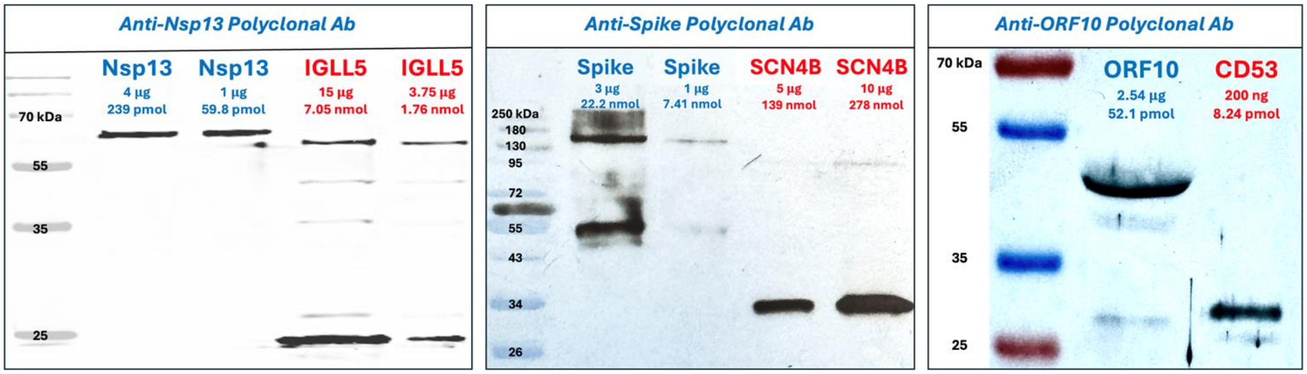

Supplementary Figure S2.

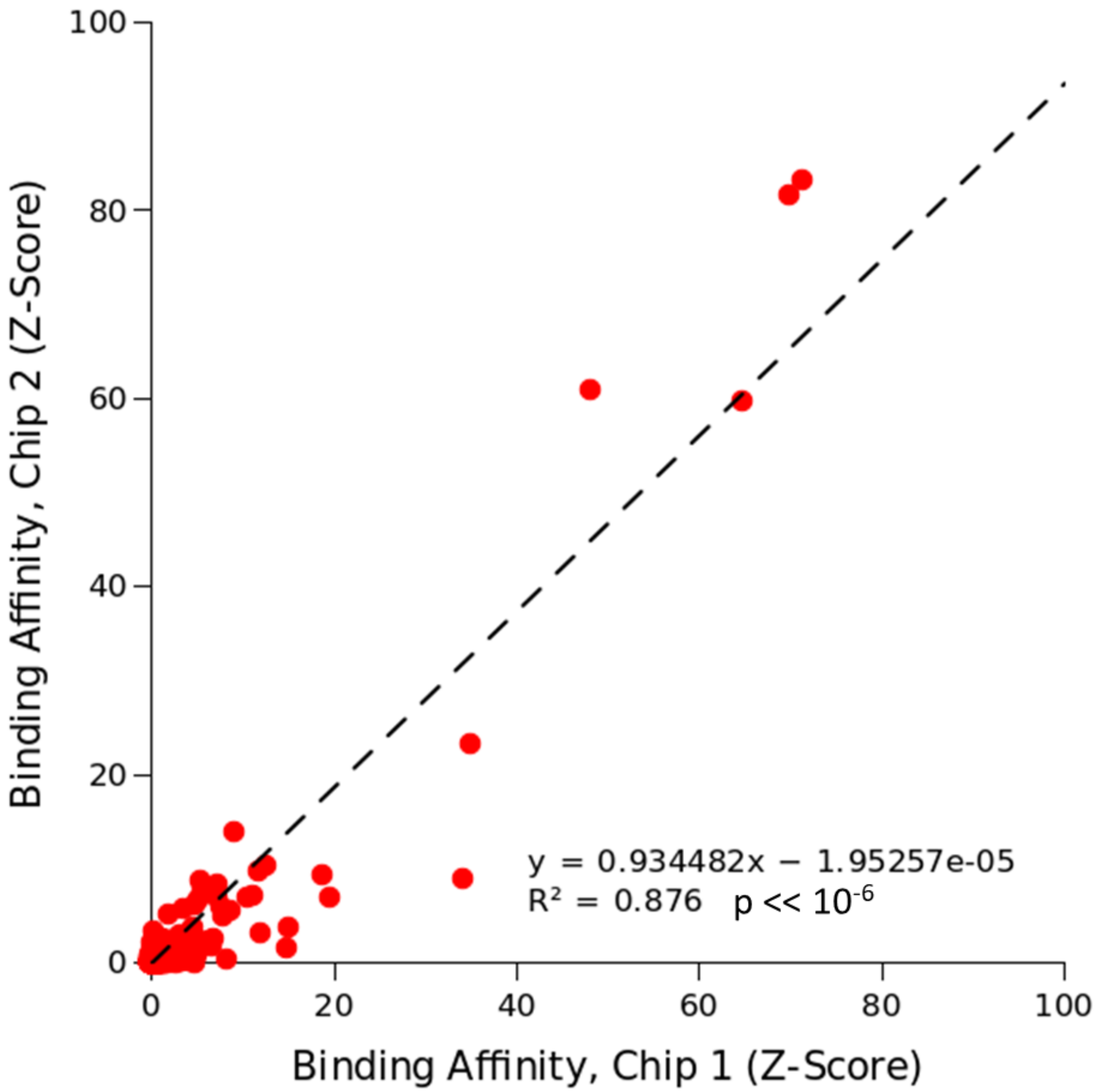

Supplementary Table S1.

Index	Rank (Native Chip)	Polyclonal Antibody	Human Protein	*HuProt* ID	Native Z-Score	Denatured Z-Score	Sum of Z-Scores	*e*TFR -log P value	*M*	*N*	*f*
1	1	Anti-orf9	NMD3	JHU03424.B15C9R78	114.951	0.069	115.020	2.869	328	410	0.80
2	2	Anti-orf9	AAMP	JHU13499.B9C17R32	61.691	71.158	132.849	3.959	345	410	0.84
3	3	Anti-orf9	ZFYVE19	JHU04797.B1C8R76	52.18	100.931	153.111	4.020	341	386	0.88
4	4	Anti-orf9	YIF1B	JHU13458.B11C2R32	24.521	35.388	59.909	3.032	101	132	0.77
5	5	Anti-orf9	DACT3	JHU03094.B3C12R50	24.001	66.453	90.454	4.381	138	171	0.81
6	6	Anti-orf9	TJP2	JHU18226.B15C2R44	22.114	14.815	36.929	6.538	268	410	0.70
7	7	Anti-orf9	PDCD4	JHU03330.B4C25R50	16.808	12.288	29.096	4.706	353	410	0.86
8	8	Anti-orf9	TJP2	JHU18226.B13C8R74	14.253	9.508	23.761	6.538	288	410	0.70
9	9	Anti-orf9	CCDC40	JHU10191.B5C24R70	13.721	11.166	24.887	6.480	349	410	0.84
10	10	Anti-orf9	KCNAB1	JHU13741.B9C28R34	8.623	0.203	8.826	2.091	333	398	0.84
11	1	Anti-nsp13	DAB1	JHU09621.B14C10R68	66.262	46.692	112.954	5.355	508	543	0.94
12	2	Anti-nsp13	DAB1	JHU10095.B6C3R66	66.262	46.692	112.954	5.758	413	459	0.90
13	3	Anti-nsp13	ABLIM1	JHU09408.B6C25R56	66.262	46.692	112.954	4.356	332	391	0.85
14	4	Anti-nsp13	DAB1	JHU10095.B5C27R68	49.575	46.692	96.267	5.758	413	459	0.90
15	5	Anti-nsp13	ANKRD49	JHU07970.B13C16R72	33.711	13.195	46.906	4.944	212	229	0.93
16	6	Anti-nsp13	CTNNA3	JHU16269.B12C19R74	29.271	14.966	44.237	9.244	486	506	0.96
17	7	Anti-nsp13	GNAS	JHU28981.B17C22R6	22.402	20.253	42.655	6.800	488	592	0.82
18	8	Anti-nsp13	NUMB_frag	JHU05240.B3C24R84	22.126	15.443	37.569	4.189	108	125	0.86
19	9	Anti-nsp13	DIS3L	JHU09250.B5C15R56	20.513	15.198	35.711	6.271	563	592	0.95
20	10	Anti-nsp13	PSMC2	JHU00355.B4C28R6	18.754	29.016	47.770	4.686	395	423	0.93
21	1	Anti-nsp16	DCX	JHU04060.B1C16R62	89.285	94.366	183.651	4.491	261	289	0.90
22	2	Anti-nsp16	DCX	JHU03009.B1C28R44	89.285	94.366	183.651	4.837	261	289	0.90
23	3	Anti-nsp16	DCX	JHU04060.B18C8R14	82.689	70.763	153.452	4.491	261	289	0.90
24	4	Anti-nsp16	DHX32	JHU10488.B8C9R74	10.144	8.481	18.625	4.695	257	289	0.89
25	5	Anti-nsp16	KIAA1191	JHU07922.B7C6R36	8.357	3.861	12.218	3.195	232	289	0.80
26	6	Anti-nsp16	IGHG4	JHU03305.B2C25R54	6.493	-0.007	6.486	3.447	254	289	0.88
27	7	Anti-nsp16	SSBP3	JHU09872.B6C6R62	4.604	12.55	17.154	1.333	206	289	0.71
28	8	Anti-nsp16	KLHDC9	JHU15417.B12C9R64	3.19	0.049	3.239	1.282	249	289	0.86
29	9	Anti-nsp16	KCNAB1	JHU13741.B9C28R34	2.969	-0.014	2.955	3.601	252	289	0.87
30	10	Anti-nsp16	OVOL2	JHU04191.B1C31R66	2.088	0.134	2.222	2.352	225	265	0.85
31	1	Anti-Spike	AZIN1	JHU00873.B1C14R14	77.633	57.351	134.984	7.082	380	438	0.87
32	2	Anti-Spike	WWP2	JHU04987.B3C7R76	44.858	50.195	95.053	5.931	274	325	0.84
33	3	Anti-Spike	BC031259.1_frag	JHU15432.B12C27R66	43.872	43.872	32.893	5.710	168	184	0.91
34	4	Anti-Spike	LARP6	JHU10982.B8C1R80	42.937	28.28	71.217	4.435	83	83	1.00
35	5	Anti-Spike	CPTP_frag	JHU02353.B2C12R42	40.305	34.807	75.112	4.433	185	211	0.88
36	6	Anti-Spike	NFYC	JHU04956.B3C10R76	38.425	46.859	85.284	5.909	278	325	0.86
37	7	Anti-Spike	ALKBH7	JHU04132.B3C31R64	38.268	33.995	72.263	3.607	182	211	0.86
38	8	Anti-Spike	GLB1	JHU21299.B13C4R86	37.06	25.823	62.883	6.711	629	667	0.94
39	9	Anti-Spike	PDPN	JHU14405.B10C6R46	28.354	32.937	61.291	5.412	107	108	0.99
40	10	Anti-Spike	GIMAP4	JHU04931.B3C28R74	28.136	43.616	71.752	5.951	310	319	0.97
41	1	Anti-orf6	AHNAK2	JHU07968.B5C13R32	7.253	3.654	10.907	2.919	50	52	0.96
42	2	Anti-orf6	TEX33	JHU20055.B17C9R4	6.356	7.144	13.500	1.571	50	52	0.96
43	3	Anti-orf6	FTH1	JHU15414.B10C8R64	5.799	7.796	13.595	2.321	42	52	0.81
44	4	Anti-orf6	DPY30	JHU03411.B1C10R50	5.673	2.94	8.613	2.196	50	52	0.96
45	5	Anti-orf6	NME1	JHU00057.B13C16R76	5.527	3.166	8.693	2.684	52	52	1.00
46	6	Anti-orf6	PYCR2	JHU04471.B1C6R70	5.494	2.644	8.138	3.521	50	52	0.96
47	7	Anti-orf6	LOR	JHU15534.B9C1R64	5.484	1.468	6.952	0.020	25	52	0.48
48	8	Anti-orf6	CCDC102B_frag	JHU15882.B9C4R72	5.435	5.435	4.038	2.309	45	52	0.87
49	9	Anti-orf6	TPD52L2	JHU04498.B4C11R72	5.39	4.371	9.761	2.164	48	52	0.92
50	10	Anti-orf6	MRFAP1L1	JHU02929.B2C1R46	5.354	2.35	7.704	2.476	51	52	0.98
51	1	Anti-orf8	CTNNB1	JHU07025.B7C17R22	90.589	85.761	176.350	4.918	112	112	1.00
52	2	Anti-orf8	PSMC3	JHU13762.B10C7R32	60.15	61.114	121.264	3.033	105	112	0.94
53	3	Anti-orf8	HNRNPK	JHU02733.B1C23R44	13.632	9.704	23.336	3.189	99	112	0.88
54	4	Anti-orf8	FBXL8	JHU19904.B14C20R8	6.735	2.859	9.594	4.997	109	112	0.97
55	5	Anti-orf8	FIGNL2	JHU17712.B13C10R58	6.113	4.104	10.217	4.024	94	112	0.84
56	6	Anti-orf8	ZNF662	JHU29296.B18C32R24	4.3	0.975	5.275	2.556	108	112	0.96
57	7	Anti-orf8	ZSCAN22	JHU19974.B15C26R10	3.412	1.599	5.011	3.087	74	112	0.66
58	8	Anti-orf8	ZSCAN30	JHU29179.B18C8R22	3.24	0.114	3.354	5.038	96	112	0.86
59	9	Anti-orf8	ZNF621	JHU11225.B7C23R90	2.612	1.248	3.860	3.041	111	112	0.99
60	10	Anti-orf8	ZNF620	JHU24458.B20C8R2	2.286	0.355	2.641	3.298	97	112	0.87
61	1	Anti-orf10	RGS10	JHU11967.B9C15R10	51.047	51.047	102.094	2.030	28	29	0.97
62	2	Anti-orf10	CYTH1	JHU05740.B1C28R90	50.771	50.771	101.542	2.962	29	29	1.00
63	3	Anti-orf10	OBFC1	JHU09466.B8C24R56	44.589	44.589	89.178	3.331	29	29	1.00
64	4	Anti-orf10	FAM13A	JHU15982.B12C21R72	36.974	36.974	73.948	3.182	29	29	1.00
65	5	Anti-orf10	OPHN1	JHU13650.B9C6R34	34.511	34.511	69.022	2.698	29	29	1.00
66	6	Anti-orf10	ADD1	JHU13212.B10C1R28	32.752	32.752	65.504	2.933	28	29	0.97
67	7	Anti-orf10	DUSP15	JHU12281.B11C20R14	20.665	20.665	41.330	2.781	29	29	1.00

68	8	Anti-orf10	TMEM173	JHU05940.B7C29R6	16.845	16.845	33.690	3.173	29	29	1.00
69	9	Anti-orf10	RGS10	JHU08427.B5C31R38	16.719	16.719	33.438	2.030	28	29	0.97
70	10	Anti-orf10	C14orf119	JHU04523.B3C30R72	14.206	14.206	28.412	2.382	19	29	0.66

Supplementary Table S2.

Index	Thermodynamically Aligned Region of Protein 1 (Virus Protein)	Thermodynamically Aligned Region of Protein 2 (Human Protein)
1	MSDNGPQNQRNAPRITFGGPSDSTGSNQNG<br>ERSGARSKQRRPQGLPNNTASWFTALTQHG<br>KEDLKFPRGQGVPINTNSSPDDQIGYYRRA<br>TRRIRGGDGKMKDLSPRWYFYYLGTGPEAG<br>LPYGANKDGIIWVATEGALNTPKDHIGTRN<br>PANNAAIVLQLPQGTTLPKGFYAEGSRGGS<br>QASSRSSSRSRNSSRNSTPGSSRGTSPARM<br>AGNGGDAALALLLLDRLNQLESKMSGKGQQ<br>QQGQTVTKKSAAEASKKPRQKRTATKAYNV<br>TQAFGRRGPEQTQGNFGDQELIRQGTDYKH<br>WPQIAQFAPSASAFFGMSRIGMEVTPSGTW<br>LTYTGAIKLDDKDPNFKDQVILLNKHIDAY<br>KTFPPTEPKKDKKKKADETQALPQRQKKQQ<br>TVTLLPAADLDDFSKQLQQSMSSADSTQA<br>orf9	SPGHILCCECGVPISPNPANICVACLRSKV<br>DISQGIPKQVSISFCKQCQRYFQPPGTWIQ<br>CALESRELLALCLKKIKAPLSKVRLVDAGF<br>VWTEPHSKRLKVKLTIQKEVMNGAILQQVF<br>VVDYVVQSQMCGDCHRVEAKDFWKAVIQVR<br>QKTLHKKTFYYLEQLILKYGMHQNTLRIKE<br>IHDGLDFYYSSKQHAQKMVEFLQCTVPCRY<br>KASQRLISQDIHSNTYNYKSTFSVEIVPIC<br>KDNVVCLSPKLAQSLGNMNQICVCIRVTSA<br>IHLIDPNTLQVADIDGSTFWSHPFNSLCHP<br>KQLEEFIVMECSIVQDIKRAAGAGMISKKH<br>TLGEVWVQKTSEMNTDKQYFCRTHLGHLLN<br>PGDLVLGFDLANCNLNDEHVNKMNSDRVPD<br>VVLIKKSYDRTKRQRRRNWKLKELARERE<br>NMD3 JHU03424
2	MSDNGPQNQRNAPRITFGGPSDSTGSNQNG<br>ERSGARSKQRRPQGLPNNTASWFTALTQHG<br>KEDLKFPRGQGVPINTNSSPDDQIGYYRRA<br>TRRIRGGDGKMKDLSPRWYFYYLGTGPEAG<br>LPYGANKDGIIWVATEGALNTPKDHIGTRN<br>PANNAAIVLQLPQGTTLPKGFYAEGSRGGS<br>QASSRSSSRSRNSSRNSTPGSSRGTSPARM<br>AGNGGDAALALLLLDRLNQLESKMSGKGQQ<br>QQGQTVTKKSAAEASKKPRQKRTATKAYNV<br>TQAFGRRGPEQTQGNFGDQELIRQGTDYKH<br>WPQIAQFAPSASAFFGMSRIGMEVTPSGTW<br>LTYTGAIKLDDKDPNFKDQVILLNKHIDAY<br>KTFPPTEPKKDKKKKADETQALPQRQKKQQ<br>TVTLLPAADLDDFSKQLQQSMSSADSTQA<br>orf9	TPPLETLSFHGDEEIIEVVELDPGPPDPDD<br>LAQEMEDVDFEEEEEEEGNEEGWVLEPQEG<br>VVGSMEGPDDSEVTFALHSASVFCVSLDPK<br>TNTLAVTGGEDDKAFVWRLSDGELLFECAG<br>HKDSVTCAGFSHDSTLVATGDMSGLLKVWQ<br>VDTKEEVWSFEAGDLEWMEWHPRAPVLLAG<br>TADGNTWMWKVPNGDCKTFQGPNCPATCGR<br>VLPDGKRAVVGYEDGTIRIWDLKQGSPIHV<br>LKGTEGHQGPLTCVAANQDGSLILTGSVDC<br>QAKLVSATTGKVVGVFRPETVASQPSLGEG<br>EESESNSVESLGFCSVMPLAAVGYLDGTLA<br>IYDLATQTLRHQCQHQSGIVQLLWEAGTAV<br>VYTCSLDGIVRLWDARTGRLLTDYRGHTAE<br>ILDFALSKDASLVVTTSGDHKAKVFCVQR<br>AAMP JHU13499
3	DNGPQNQRNAPRITFGGPSDSTGSNQNGER<br>SGARSKQRRPQGLPNNTASWFTALTQHGKE<br>DLKFPRGQGVPINTNSSPDDQIGYYRRATR<br>RIRGGDGKMKDLSPRWYFYYLGTGPEAGLP<br>YGANKDGIIWVATEGALNTPKDHIGTRNPA<br>NNAAIVLQLPQGTTLPKGFYAEGSRGGSQA<br>SSRSSSRSRNSSRNSTPGSSRGTSPARMAG<br>NGGDAALALLLLDRLNQLESKMSGKGQQQQ<br>GQTVTKKSAAEASKKPRQKRTATKAYNVTQ<br>AFGRRGPEQTQGNFGDQELIRQGTDYKHWP<br>QIAQFAPSASAFFGMSRIGMEVTPSGTWLT<br>YTGAIKLDDKDPNFKDQVILLNKHIDAYKT<br>FPPTEPKKDKKKKADETQALPQRQKKQQTV<br>TLLPA orf9	MESRCYGCAVKFTLFKKEYGCKNCGRAFCS<br>GCLSFSAAVPRTGNTQQKVCKQCHEVLTRG<br>SSANASKWSPPQNYKKRVAALEAKQKPSTS<br>QSQGLTRQDQMIAERLARLRQENKPKLVPS<br>QAEIEARLAALKDERQGSIPSTQEMEARLA<br>ALQGRVLPSQTPQPAHHTPDTRTQAQQTQD<br>LLTQLAAEVAIDESWKGGGPAASLQNDLNQ<br>GGPGSTNSKRQANWSLEEEKSRLLAEAALE<br>LREENTRQERILALAKRLAMLRGQDPERVT<br>LQDYRLPDSDDDEDEETAIQRVLQQLTEEA<br>ALDEASGFNIPAEQASRPWTQPRGAEPEAQ<br>DVDPRPEAEEEELPWCCICNEDATLRCAGC<br>DGDLFCARCFREGHDAFELKEHQTSAYSPP<br>RAGQE ZFYVE19 JHU04797
4	SSRSSSRSRNSSRNSTPGSSRGTSPARMAG<br>NGGDAALALLLLDRLNQLESKMSGKGQQQQ<br>GQTVTKKSAAEASKKPRQKRTATKAYNVTQ<br>AFGRRGPEQTQGNFGDQELIRQGTDYKHWP<br>QIAQFAPSASAFFGMSRIGME orf9	MLSCCLEPAAWDCGAEGRDGREEMVLGWKR<br>DRGTTPRTLPASPSAGTPLWGAGHVLGDAG<br>ESPLPSHPCPNRWSCCWSRKSCGVLAGWRC<br>GSEAAPLTQPPEVGGLRQDPGVRGNPSLPA

		PPQPVMRRLTFGPGPAPASDR YIF1B JHU13458
5	GGSQASSRSSSRSRNSSRNSTPGSSRGTSPARMAGNGGDAALALLLLDRLNQLESKMSGKGQQQQGQTVTKKSAAEASKKPRQKRTATKAYNVTQAFGRRGPEQTQGNFGDQELIRQGTDYKHWPQIAQFAPSASAFFGMSRIGMEVTPSGTWLTYTGAIKLDDKDPNFKDQVILLNKHI orf9	MIRAFSFPVSPERGRLRGWLEGSLAGLCELHWLRERQEYRVQQALRLAQPGMGGAEAEDEEDADEDEDAAAARRAAAALEEQLEALPGLVWDLGQQLGDLSLESGGLEQESGRSSGFYEDPSSTGGPDSPPSTFCGDSGFSGSSSYGRLGPSEPRGIYASERPKSLGKVGEGGALGPGKG DACT3 JHU03094
6	MSDNGPQNQRNAPRITFGGPSDSTGSNQNGERSGARSKQRRPQGLPNNTASWFTALTQHGKEDLKFPRGQGVPINTNSSPDDQIGYYRRATRRIRGGDGKMKDLSPRWYFYYLGTGPEAGLPYGANKDGIIWVATEGALNTPKDHIGTRNPANNAAIVLQLPQGTTLPKGFYAEGSRGGSQASSRSSSRSRNSSRNSTPGSSRGTSPARMAGNGGDAALALLLLDRLNQLESKMSGKGQQQQGQTVTKKSAAEASKKPRQKRTATKAYNVTQAFGRRGPEQTQGNFGDQELIRQGTDYKHWPQIAQFAPSASAFFGMSRIGMEVTPSGTWLTYTGAIKLDDKDPNFKDQVILLNKHIDAYKTFPPTEPKKDKKKKADETQALPQRQKKQQTVTLLPAADLDDFSKQLQQSMSSADSTQA orf9	AKTEPKDAGSEKSTGVVRLNTVRQIIEQDKHALLDVTPKAVDLLNYTQWFPIVIFFNPDSRQGVKTMRQRLNPTSNKSSRKLFDQANKLKKTCAHLFTATINLNSANDSWFGSLKDTIQHQQGEAVWVSEGKMEGMDDDPEDRMSYLTAMGADYLSCDSRLISDFEDTDGEGGAYTDNELDEPAEEPLVSSITRSSEPVQHEESIRKPSPEPRAQMRRAASSDQLRDNSPPPAFKPEPPKAKTQNKEESYDFSKSYEYKSNPSAVAGNETPGASTKGYPPPVAAKPTFGRSILKPSTPIPPQEGEEVGESSEEQDNAPKSVLGKVKIFEKMDHKARLQRMQELQEAQNARIEIAQKHPDIYAVPIKTHKPDPGTPQHTSSRPPEPQKAPSRPYQDTRGSYGSDAEEEEYRQQLSEHSKR TJP2 JHU18226
7	MSDNGPQNQRNAPRITFGGPSDSTGSNQNGERSGARSKQRRPQGLPNNTASWFTALTQHGKEDLKFPRGQGVPINTNSSPDDQIGYYRRATRRIRGGDGKMKDLSPRWYFYYLGTGPEAGLPYGANKDGIIWVATEGALNTPKDHIGTRNPANNAAIVLQLPQGTTLPKGFYAEGSRGGSQASSRSSSRSRNSSRNSTPGSSRGTSPARMAGNGGDAALALLLLDRLNQLESKMSGKGQQQQGQTVTKKSAAEASKKPRQKRTATKAYNVTQAFGRRGPEQTQGNFGDQELIRQGTDYKHWPQIAQFAPSASAFFGMSRIGMEVTPSGTWLTYTGAIKLDDKDPNFKDQVILLNKHIDAYKTFPPTEPKKDKKKKADETQALPQRQKKQQTVTLLPAADLDDFSKQLQQSMSSADSTQA orf9	SGDEENAGTEEVKNEINGNWISAYSINEARINAKAKRRLRKNSSRDSGRGDSVSDSGSDALRSGLTVPTSPKGRLLDRRSRSGKGRGLPKKGGAGGKGVWGTPGQVYDVEEVDVKDPNYDDDQENCVYETVVLPLDERAFEKTLTPIIQEYFEHGDTNEVAEMLRDLNLGEMKSGVPVLAVSLALEGKASHREMTSKLLSDLCGTVMSTTDVEKSFDKLLKDLPELALDTPRAPQLVGQFIARAVGDGILCNTYIDSYKGTVDCVQARAALDKATVLLSMSKGGKRKDSVWGSGGGQQSVNHLVKEIDMLLKEYLLSGDISEAEHCLKELEVPHFHHELVYEAIIMVLESTGESTFKMILDLLKSLWKSSTITVDQMKRGYERIYNEIPDINLDVPHSYSVLERFVEECFQAGIISKQL PDCD4 JHU03330
8	MSDNGPQNQRNAPRITFGGPSDSTGSNQNGERSGARSKQRRPQGLPNNTASWFTALTQHGKEDLKFPRGQGVPINTNSSPDDQIGYYRRATRRIRGGDGKMKDLSPRWYFYYLGTGPEAGLPYGANKDGIIWVATEGALNTPKDHIGTRNPANNAAIVLQLPQGTTLPKGFYAEGSRGGSQASSRSSSRSRNSSRNSTPGSSRGTSPARMAGNGGDAALALLLLDRLNQLESKMSGKGQQQQGQTVTKKSAAEASKKPRQKRTATKAYNVTQAFGRRGPEQTQGNFGDQELIRQGTDYKHWPQIAQFAPSASAFFGMSRIGMEVTPSGTWLTYTGAIKLDDKDPNFKDQVILLNKHIDAYKTFPPTEPKKDKKKKADETQALPQRQKKQQTVTLLPAADLDDFSKQLQQSMSSADSTQA orf9	AKTEPKDAGSEKSTGVVRLNTVRQIIEQDKHALLDVTPKAVDLLNYTQWFPIVIFFNPDSRQGVKTMRQRLNPTSNKSSRKLFDQANKLKKTCAHLFTATINLNSANDSWFGSLKDTIQHQQGEAVWVSEGKMEGMDDDPEDRMSYLTAMGADYLSCDSRLISDFEDTDGEGGAYTDNELDEPAEEPLVSSITRSSEPVQHEESIRKPSPEPRAQMRRAASSDQLRDNSPPPAFKPEPPKAKTQNKEESYDFSKSYEYKSNPSAVAGNETPGASTKGYPPPVAAKPTFGRSILKPSTPIPPQEGEEVGESSEEQDNAPKSVLGKVKIFEKMDHKARLQRMQELQEAQNARIEIAQKHPDIYAVPIKTHKPDPGTPQHTSSRPPEPQKAPSRPYQDTRGSYGSDAEEEEYRQQLSEHSKR TJP2 JHU18226

9	MSDNGPQNQRNAPRITFGGPSDSTGSNQNG<br>ERSGARSKQRRPQGLPNNTASWFTALTQHG<br>KEDLKFPRGQGVPINTNSSPDDQIGYYRRA<br>TRRIRGGDGKMKDLSPRWYFYYLGTGPEAG<br>LPYGANKDGIIWVATEGALNTPKDHIGTRN<br>PANNAAIVLQLPQGTTLPKGFYAEGSRGGS<br>QASSRSSSRSRNSSRNSTPGSSRGTSPARM<br>AGNGGDAALALLLLDRLNQLESKMSGKGQQ<br>QQGQTVTKKSAAEASKKPRQKRTATKAYNV<br>TQAFGRRGPEQTQGNFGDQELIRQGTDYKH<br>WPQIAQFAPSASAFFGMSRIGMEVTPSGTW<br>LTYTGAIKLDDKDPNFKDQVILLNKHIDAY<br>KTFPPTEPKKDKKKKADETQALPQRQKKQQ<br>TVTLLPAADLDDFSKQLQQSMSSADSTQA<br>orf9	EGEKEGNNESHMVSPPEKDDGQKGEEAVGS<br>TEHPEEVTTQAEAAIEEGEVETEGEAAVEG<br>EEEAVSYGDAESEEEYYYTETSSPEGQISA<br>ADTTYPYFSPPQELPGEEAYDSVSGEAGLQ<br>GFQQEATGPPESRERRVTSPEPSHGVLGPS<br>EQMGQVTSGPAVGRLTGSTEEPQGQVLPMG<br>VQHRFRLSHGSDIESSDLEEFVSQEPVIPP<br>GVPDAHPREGDLPVFQDQIQQPSTEEGAMA<br>ERVESEGSDEEAEDEGSQLVVLDPDHPLMV<br>RFQAALKNYLNRQIEKLKLDLQELVVATKQ<br>SRAQRQELGVNLYEVQQHLVHLQKLLEKSH<br>DRHAMASSERRQKEEELQAARALYTKTCAA<br>ANEERKKLAALQTEMENLALHLFYMQNIDQ<br>DMRDDIRVMTQVVKKAETERIRAEIEKKK<br>CCDC40 JHU10191
10	RNAPRITFGGPSDSTGSNQNGERSGARSKQ<br>RRPQGLPNNTASWFTALTQHGKEDLKFPRG<br>QGVPINTNSSPDDQIGYYRRATRRIRGGDG<br>KMKDLSPRWYFYYLGTGPEAGLPYGANKDG<br>IIWVATEGALNTPKDHIGTRNPANNAAIVL<br>QLPQGTTLPKGFYAEGSRGGSQASSRSSSR<br>SRNSSRNSTPGSSRGTSPARMAGNGGDAAL<br>ALLLLDRLNQLESKMSGKGQQQQGQTVTKK<br>SAAEASKKPRQKRTATKAYNVTQAFGRRGP<br>EQTQGNFGDQELIRQGTDYKHWPQIAQFAP<br>SASAFFGMSRIGMEVTPSGTWLTYTGAIKL<br>DDKDPNFKDQVILLNKHIDAYKTFPPTEPK<br>KDKKKKADETQALPQRQKKQQTVTLLPAAD<br>LDDFSKQLQQSMSSADS orf9	MHLYKPACADIPSPKLGLPKSSESALKCRW<br>HLAVTKTQPQAACKPVRPSGAAEQKYVEKF<br>LRVHGISLQETTRAETGMAYRNLGKSGLRV<br>SCLGLGTWVTFGGQISDEVAERLMTIAYES<br>GVNLFDTAEVYAAGKAEVILGSIIKKKGWR<br>RSSLVITTKLYWGGKAETERGLSRKHIIEG<br>LKGSLQRLQLEYVDVVFANRPDSNTPMEEI<br>VRAMTHVINQGMAMYWGTSRWSAMEIMEAY<br>SVARQFNMIPPVCEQAEYHLFQREKVEVQL<br>PELYHKIGVGAMTWSPLACGIISGKYGNGV<br>PESSRASLKCYQWLKERIVSEEGRKQQNKL<br>KDLSPIAERLGCTLPQLAVAWCLRNEGVSS<br>VLLGSSTPEQLIENLGAIQVLPKMTSHVVN<br>EIDNILRNKPYSKKDYR KCNAB1<br>JHU13741
11	YDHVISTSHKLVLSVNPYVCNAPGCDVTDV<br>TQLYLGGMSYYCKSHKPPISFPLCANGQVF<br>GLYKNTCVGSDNVTDFNAIATCDWTNAGDY<br>ILANTCTERLKLFAAETLKATEETFKLSYG<br>IATVREVLSDRELHLSWEVGKPRPPLNRNY<br>VFTGYRVTKNSKVQIGEYTFEKGDYGDAVV<br>YRGTTTYKLNVGDYFVLTSHTVMPLSAPTL<br>VPQEHYVRITGLYPTLNISDEFSSNVANYQ<br>KVGMQKYSTLQGPPGTGKSHFAIGLALYYP<br>SARIVYTACSHAAVDALCEKALKYLPIDKC<br>SRIIPARARVECFDKFKVNSTLEQYVFCTV<br>NALPETTADIVVFDEISMATNYDLSVVNAR<br>LRAKHYVYIGDPAQLPAPRTLLTKGTLEPE<br>YFNSVCRLMKTIGPDMFLGTCRRCPAEIVD<br>TVSALVYDNKLKAHKDKSAQCFKMFYKGVI<br>THDVSSAINRPQIGVVREFLTRNPAWRKAV<br>FISPYNSQNAVASKILGLPTQTVDSSQGSE<br>YDYVIFTQTTETAHSCNVNRFNVAITRAKV<br>GILCIMSDRDLY nsp13	MSTETELQVAVKTSAKKDSRKKGQDRSEAT<br>LIKRFKGEGVRYKAKLIGIDEVSAARGDKL<br>CQDSMMKLKGVVAGARSKGEHKQKIFLTIS<br>FGGIKIFDEKTGALQHHHAVHEISYIAKDI<br>TDHRAFGYVCGKEGNHRFVAIKTAQAAEPV<br>ILDLRDLFQLIYELKQREELEKKAQKDKQC<br>EQAVYQVPTSQKKEGVYDVPKSQPVSNGYS<br>FEDFEERFAAATPNRNLPTDFDEIFEATKA<br>VTQLELFGDMSTPPDITSPPTPATPGDAFI<br>PSSSQTLPASADVFSSVPFGTAAVPSGYVA<br>MGAVLPSFWGQQPLVQQQMVMGAQPPVAQV<br>MPGAQPIAWGQPGLFPATQQPWPTVAGQFP<br>PAAFMPTQTVMPLPAAMFQGPLTPLATVPG<br>TSDSTRSSPQTDKPRQKMGKETFKDFQMAQ<br>PPPVPSRKPDQPSLTCTSEAFSSYFNKVGV<br>AQDTDDCDDFDISQLNLTPVTSTTPSTNSP<br>PTPAPRQSSPSKSSASHASDPTTDDIFEEG<br>FESPSKSEEQEAPDGSQASSNSDPFGEPSG<br>EPSGDNISPQAG DAB1 JHU09621
12	WTNAGDYILANTCTERLKLFAAETLKATEE<br>TFKLSYGIATVREVLSDRELHLSWEVGKPR<br>PPLNRNYVFTGYRVTKNSKVQIGEYTFEKG<br>DYGDAVVYRGTTTYKLNVGDYFVLTSHTVM<br>PLSAPTLVPQEHYVRITGLYPTLNISDEFS	MSTETELQVAVKTSAKKDSRKKGQDRSEAT<br>LIKRFKGEGVRYKAKLIGIDEVSAARGDML<br>CQDSMMKLKGVVAGARSKGEHKQKIFLTIS<br>FGGIKIFDEKTGVPTSQKKEGVYDVPKSQP<br>VSNGYSFEDFEERFAAATPNRNLPTDFDEI

	SNVANYQKVGMQKYSTLQGPPGTGKSHFAI GLALYYPSARIVYTACSHAAVDALCEKALK YLPIDKCSRIIPARARVECFDKFKVNSTLE QYVFCTVNALPETTADIVVFDEISMATNYD LSVVNARLRAKHYVYIGDPAQLPAPRTLLT KGTLEPEYFNSVCRLMKTIGPDMFLGTCRR CPAEIVDTVSALVYDNKLKAHKDKSAQCFK MFYKGVITHDVSSAINRPQIGVVREFLTRN PAWRKAVFISPYNSQNAVASKILGLPTQTV DSSQGSEYDYVIFTQTTETAHSCNVNRFNV AITRAKVGILCIMSDRDL nsp13	FEATKAVTQLELFGDMSTPPDITSPSTPAT PGDAFIPSSSQTLPASADVFSSVPFGTAAV PSGYVAMGAVLPSFWGQQPLVQQQMVMGAQ PPVAQVMPGAQPIAWGQPGLFPATQQPWPT VAGQFPPAAFMPTQTVMPLPAAMFQGPLTP LATVPGTSDSTRSSPQTDKPRQKMGKETFK DFQMAQPPPVPSRKPDQPSLTCTSEAFSSY FNKVGVAQDTDDCDDFDISQLNLTPVTSTT PSTNSPPTPAPRQSSPSKSSASHASDPTTD DIFEEGFESPSKSEEQEAPDGSQASSNSDP FGEPSGEPSGDNISPQAG DAB1 JHU10095
13	CTERLKLFAAETLKATEETFKLSYGIATVR EVLSDRELHLSWEVGKPRPPLNRNYVFTGY RVTKNSKVQIGEYTFEKGDYGDAVVYRGTT TYKLNVGDYFVLTSHTVMPLSAPTLVPQEH YVRITGLYPTLNISDEFSSNVANYQKVGMQ KYSTLQGPPGTGKSHFAIGLALYYPSARIV YTACSHAAVDALCEKALKYLPIDKCSRIIP ARARVECFDKFKVNSTLEQYVFCTVNALPE TTADIVVFDEISMATNYDLSVVNARLRAKH YVYIGDPAQLPAPRTLLTKGTLEPEYFNSV CRLMKTIGPDMFLGTCRRCPAEIVDTVSAL VYDNKLKAHKDKSAQCFKMFYKGVITHDVS SAINRPQIGVVREFLTRNPAWRKAVFISPY NSQNAVASKI nsp13	MFTEGEEMYLQGSTVWHPDCKQSTKTEEKL RAKVDNEILDYKDLAAIPKVKAIYDIERPD LITYEPFYTSGYDDKQERQSLGESPRTLSP TPSAEGYQDVRDRMIHRSTSQGSINSPVYS RHSYTPTTSRSPQHFHRPDQGINIYRKPPI YKQHAALAAQSKSSEDIIKFSKFPAAQAPD PSETPKIETDHWPGPPSFAVVGPDMKRRSS GREEDDEELLRRRQLQEEQLMKLNSGLGQL ILKEEMEKESRERSSLLASRYDSPINSASH IPSSKTASLPGYGRNGLHRPVSTDFAQYNS YGDVSGGVRDYQTLPDGHMPAMRMDRGVSM PNMLEPKIFPYEMLMVTNRGRNKILREVDR TRLERHLAPEVFREIFGMSIQEFDRLPLWR RNDMKKKAKL ABLIM1 JHU09408
14	WTNAGDYILANTCTERLKLFAAETLKATEE TFKLSYGIATVREVLSDRELHLSWEVGKPR PPLNRNYVFTGYRVTKNSKVQIGEYTFEKG DYGDAVVYRGTTTYKLNVGDYFVLTSHTVM PLSAPTLVPQEHYVRITGLYPTLNISDEFS SNVANYQKVGMQKYSTLQGPPGTGKSHFAI GLALYYPSARIVYTACSHAAVDALCEKALK YLPIDKCSRIIPARARVECFDKFKVNSTLE QYVFCTVNALPETTADIVVFDEISMATNYD LSVVNARLRAKHYVYIGDPAQLPAPRTLLT KGTLEPEYFNSVCRLMKTIGPDMFLGTCRR CPAEIVDTVSALVYDNKLKAHKDKSAQCFK MFYKGVITHDVSSAINRPQIGVVREFLTRN PAWRKAVFISPYNSQNAVASKILGLPTQTV DSSQGSEYDYVIFTQTTETAHSCNVNRFNV AITRAKVGILCIMSDRDL nsp13	MSTETELQVAVKTSAKKDSRKKGQDRSEAT LIKRFKGEGVRYKAKLIGIDEVSAARGDML CQDSMMKLKGVVAGARSKGEHKQKIFLTIS FGGIKIFDEKTGVPTSQKKEGVYDVPKSQP VSNGYSFEDFEERFAAATPNRNLPTDFDEI FEATKAVTQLELFGDMSTPPDITSPSTPAT PGDAFIPSSSQTLPASADVFSSVPFGTAAV PSGYVAMGAVLPSFWGQQPLVQQQMVMGAQ PPVAQVMPGAQPIAWGQPGLFPATQQPWPT VAGQFPPAAFMPTQTVMPLPAAMFQGPLTP LATVPGTSDSTRSSPQTDKPRQKMGKETFK DFQMAQPPPVPSRKPDQPSLTCTSEAFSSY FNKVGVAQDTDDCDDFDISQLNLTPVTSTT PSTNSPPTPAPRQSSPSKSSASHASDPTTD DIFEEGFESPSKSEEQEAPDGSQASSNSDP FGEPSGEPSGDNISPQAG DAB1 JHU10095
15	PGCDVTDVTQLYLGGMSYYCKSHKPPISFP LCANGQVFGLYKNTCVGSDNVTDFNAIATC DWTNAGDYILANTCTERLKLFAAETLKATE ETFKLSYGIATVREVLSDRELHLSWEVGKP RPPLNRNYVFTGYRVTKNSKVQIGEYTFEK GDYGDAVVYRGTTTYKLNVGDYFVLTSHTV MPLSAPTLVPQEHYVRITGLYPTLNISDEF SSNVANYQKVGMQKYSTLQGPPGTGKSH nsp13	MEKEKGNDDGIPDQENSLDFSEHFNQLELL ETHGHLIPTGTQSLWVGNSDEDEEQDDKNE EWYRLQEKKMEKDPSRLLLWAAEKNRLTTV RRLLSEKATHVNTRDEDEYTPLHRAAYSGH LDIVQELIAQGADVHAVTVDGWTPLHSACK WNNTRVASFLLQHDADINAQTKGLLTPLHL AAGNRDSKDTLELLLMNRYVKPGLKNNLEE TAFDIARRTSIYHYLFEIVEGCTNSSPQ ANKRD49 JHU07970
16	APGCDVTDVTQLYLGGMSYYCKSHKPPISF PLCANGQVFGLYKNTCVGSDNVTDFNAIAT	MSAETPITLNIDPQDLQVQTFTVEKLLEPL IIQVTTLVNCPQNPSSRKKGRSKRASVLLA

	CDWTNAGDYILANTCTERLKLFAAETLKAT<br>EETFKLSYGIATVREVLSDRELHLSWEVGK<br>PRPPLNRNYVFTGYRVTKNSKVQIGEYTFE<br>KGDYGDAVVYRGTTTYKLNVGDYFVLTSHT<br>VMPLSAPTLVPQEHYVRITGLYPTLNISDE<br>FSSNVANYQKVGMQKYSTLQGPPGTGKSHF<br>AIGLALYYPSARIVYTACSHAAVDALCEKA<br>LKYLPIDKCSRIIPARARVECFDKFKVNST<br>LEQYVFCTVNALPETTADIVVFDEISMATN<br>YDLSVVNARLRAKHYVYIGDPAQLPAPRTL<br>LTKGTLEPEYFNSVCRLMKTIGPDMFLGTC<br>RRCPAEIVDTVSALVYDNKLKAHKDKSAQC<br>FKMFYKGVITHDVSSAINRPQIGVVREFLT<br>RNPAWRKAVFISPYNSQNAVASKILGLPTQ<br>TVDSSQGSEYDYVIFTQTTETAHSCNVNRF<br>NVAIT nsp13	SVEEATWNLLDKGEKIAQEATVLKDELTAS<br>LEEVRKESEALKVSAERFTDDPCFLPKREA<br>VVQAARALLAAVTRLLILADMIDVMCLLQH<br>VSAFQRTFESLKNVANKSDLQKTYQKLGKE<br>LENLDYLAFKRQQDLKSPNQRDEIAGARAS<br>LKENSPLLHSICSACLEHSDVASLKASKDT<br>VCEEIQNALNVISNASQGIQNMTTPPEPQA<br>ATLGSALDELENLIVLNPLTVTEEEIRPSL<br>EKRLEAIISGAALLADSSCTRDLHRERIIA<br>ECNAIRQALQDLLSEYMNNAGKKERSNTLN<br>IALDNMCKKTRDLRRQLRKAIIDHVSDSFL<br>DTTVPLLVLIEAAKNGREKEIKEYAAIFHE<br>HTSRLVEVANLACSMSTNEDGIKIVKIAAN<br>HLETLCPQIINAALALAARPKSQAVKNTME<br>MYKRTWENHIHVLTEAVDDITSIDDFLAVS<br>GMFLF CTNNA3 JHU16269
17	AVGACVLCNSQTSLRCGACIRRPFLCCKCC<br>YDHVISTSHKLVLSVNPYVCNAPGCDVTDV<br>TQLYLGGMSYYCKSHKPPISFPLCANGQVF<br>GLYKNTCVGSDNVTDFNAIATCDWTNAGDY<br>ILANTCTERLKLFAAETLKATEETFKLSYG<br>IATVREVLSDRELHLSWEVGKPRPPLNRNY<br>VFTGYRVTKNSKVQIGEYTFEKGDYGDAVV<br>YRGTTTYKLNVGDYFVLTSHTVMPLSAPTL<br>VPQEHYVRITGLYPTLNISDEFSSNVANYQ<br>KVGMQKYSTLQGPPGTGKSHFAIGLALYYP<br>SARIVYTACSHAAVDALCEKALKYLPIDKC<br>SRIIPARARVECFDKFKVNSTLEQYVFCTV<br>NALPETTADIVVFDEISMATNYDLSVVNAR<br>LRAKHYVYIGDPAQLPAPRTLLTKGTLEPE<br>YFNSVCRLMKTIGPDMFLGTCRRCPAEIVD<br>TVSALVYDNKLKAHKDKSAQCFKMFYKGVI<br>THDVSSAINRPQIGVVREFLTRNPAWRKAV<br>FISPYNSQNAVASKILGLPTQTVDSSQGSE<br>YDYVIFTQTTETAHSCNVNRFNVAITRAKV<br>GILCIMSDRDLYDKLQFTSLEIPRRNVATL<br>Q nsp13	PALMEPGAFSGARPGLGGYSPPPEEAMPFE<br>FDQPAQRGCSQLLLQVPDLAPGGPGAAGVP<br>GAPPEEPQALRPAKAGSRGGYSPPPEETMP<br>FELDGEGFGDDSPPPGLSRVIAQVDGSSQF<br>AAVAASSAVRLTPAANAPPLWVPGAIGSPS<br>QEAVRPPSNFTGSSPWMEISGPPFEIGSAP<br>AGVDDTPVNMDSPPIALDGPPIKVSGAPDK<br>RERAERPPVEEEAAEMEGAADAAEGGKVPS<br>PGYGSPAAGAASADTAARAAPAAPADPDSG<br>ATPEDPDSGTAPADPDSGAFAADPDSGAAP<br>AAPADPDSGAAPDAPADPDSGAAPDAPADP<br>DAGAAPEAPAAPAAAETRAAHVAPAAPDAG<br>APTAPAASATRAAQVRRAASAAPASGARRK<br>IHLRPPSPEIQAADPPTPRPTRASAWRGKS<br>ESSRGRRVYYDEGVASSDDDSSGDESDDGT<br>SGCLRWFQHRRNRRRRKPQRNLLRNFLVQA<br>FGGCFGRSESPQPKASRSLKVKKVPLAEKR<br>RQMRKEALEKRAQKRAEKKRSKLIDKQLQD<br>EKMGYMCTHRLLLLGAGESGKSTIVKQMRI<br>LHVNGFNGEGGEEDPQAARSNSDGEKATKV<br>Q GNAS JHU28981
18	EYTFEKGDYGDAVVYRGTTTYKLNVGDYFV<br>LTSHTVMPLSAPTLVPQEHYVRITGLYPTL<br>NISDEFSSNVANYQKVGMQKYSTLQGPPGT<br>GKSHFAIGLALYYPSARIVYTACSHAAVDA<br>LCEKALKYLPIDKC nsp13	MPYPAPNVPVVGITPSQMVANVFGTAGHPQ<br>AAHPHQSPSLVRQQTFPHYEASSATTSPFF<br>KPPAQHLNGSAAFNGVDDGRLASADRHTEV<br>PTGTCPVDPFEAQWAALENKSKQRTNPSPT<br>NPFSSDLQKTFEIE NUMB_frag<br>JHU05240
19	AVGACVLCNSQTSLRCGACIRRPFLCCKCC<br>YDHVISTSHKLVLSVNPYVCNAPGCDVTDV<br>TQLYLGGMSYYCKSHKPPISFPLCANGQVF<br>GLYKNTCVGSDNVTDFNAIATCDWTNAGDY<br>ILANTCTERLKLFAAETLKATEETFKLSYG<br>IATVREVLSDRELHLSWEVGKPRPPLNRNY<br>VFTGYRVTKNSKVQIGEYTFEKGDYGDAVV<br>YRGTTTYKLNVGDYFVLTSHTVMPLSAPTL<br>VPQEHYVRITGLYPTLNISDEFSSNVANYQ<br>KVGMQKYSTLQGPPGTGKSHFAIGLALYYP<br>SARIVYTACSHAAVDALCEKALKYLPIDKC	ESHGKEYPEHLPLEVLEAGIKSGRYIQGIL<br>NVNKHRAQIEAFVRLQGASSKDSDLVSDIL<br>IHGMKARNRSIHGDVVVVELLPKNEWKGRT<br>VALCENDCDDKASGESPSEPMPTGRVVGIL<br>QKNWRDYVVTFPSKEEVQSQGKNAQKILVT<br>PWDYRIPKIRISTQQAETLQDFRVVVRIDS<br>WESTSVYPNGHFVRVLGRIGDLEGEIATIL<br>VENSISVIPFSEAQMCEMPVNTPESPWKVS<br>PEEEQKRKDLRKSHLVFSIDPKGCEDVDDT<br>LSVRTLNNGNLELGVHIADVTHFVAPNSYI<br>DIEARTRATTYYLADRRYDMLPSVLSADLC

	SRIIPARARVECFDKFKVNSTLEQYVFCTV<br>NALPETTADIVVFDEISMATNYDLSVVNAR<br>LRAKHYVYIGDPAQLPAPRTLLTKGTLEPE<br>YFNSVCRLMKTIGPDMFLGTCRRCPAEIVD<br>TVSALVYDNKLKAHKDKSAQCFKMFYKGVI<br>THDVSSAINRPQIGVVREFLTRNPAWRKAV<br>FISPYNSQNAVASKILGLPTQTVDSSQGSE<br>YDYVIFTQTTETAHSCNVNRFNVAITRAKV<br>GILCIMSDRDLYDKLQFTSLEIPRRNVATL<br>Q nsp13	SLLGGVDRYAVSIMWELDKASYEIKKVWYG<br>RTIIRSAYKLFYEAAQELLDGNLSVVDDIP<br>EFKDLDEKSRQAKLEELVWAIGKLTDIARH<br>VRAKRDGCGALELEGVEVCVQLDDKKNIHD<br>LIPKQPLEVHETVAECMILANHWVAKKIWE<br>SFPHQALLRQHPPPHQEFFSELRECAKAKG<br>FFIDTRSNKTLADSLDNANDPHDPIVNRLL<br>RSMATQAMSNALYFSTGSCAEEEFHHYGLA<br>LDKYTHFTSPIRRYSDIVVHRLLMAAISKD<br>K DIS3L JHU09250
20	LKLFAAETLKATEETFKLSYGIATVREVLS<br>DRELHLSWEVGKPRPPLNRNYVFTGYRVTK<br>NSKVQIGEYTFEKGDYGDAVVYRGTTTYKL<br>NVGDYFVLTSHTVMPLSAPTLVPQEHYVRI<br>TGLYPTLNISDEFSSNVANYQKVGMQKYST<br>LQGPPGTGKSHFAIGLALYYPSARIVYTAC<br>SHAAVDALCEKALKYLPIDKCSRIIPARAR<br>VECFDKFKVNSTLEQYVFCTVNALPETTAD<br>IVVFDEISMATNYDLSVVNARLRAKHYVYI<br>GDPAQLPAPRTLLTKGTLEPEYFNSVCRLM<br>KTIGPDMFLGTCRRCPAEIVDTVSALVYDN<br>KLKAHKDKSAQCFKMFYKGVITHDVSSAIN<br>RPQIGVVREFLTRNPAWRKAVFISPYNSQN<br>AVASKILGLPTQTVDSSQGSEYDYVIFTQT<br>TETAHSCNVNRF nsp13	MPDYLGADQRKTKEDEKDDKPIRALDEGDI<br>ALLKTYGQSTYSRQIKQVEDDIQQLLKKIN<br>ELTGIKESDTGLAPPALWDLAADKQTLQSE<br>QPLQVARCTKIINADSEDPKYIINVKQFAK<br>FVVDLSDQVAPTDIEEGMRVGVDRNKYQIH<br>IPLPPKIDPTVTMMQVEEKPDVTYSDVGGC<br>KEQIEKLREVVETPLLHPERFVNLGIEPPK<br>GVLLFGPPGTGKTLCARAVANRTDACFIRV<br>IGSELVQKYVGEGARMVRELFEMARTKKAC<br>LIFFDEIDAIGGARFDDGAGGDNEVQRTML<br>ELINQLDGFDPRGNIKVLMATNRPDTLDPA<br>LMRPGRLDRKIEFSLPDLEGRTHIFKIHAR<br>SMSVERDIRFELLARLCPNSTGAEIRSVCT<br>EAGMFAIRARRKIATEKDFLEAVNKVIKSY<br>AKFSATPRYMTY PSMC2 JHU00355
21	SSQAWQPGVAMPNLYKMQRMLLEKCDLQNY<br>GDSATLPKGIMMNVAKYTQLCQYLNTLTLA<br>VPYNMRVIHFGAGSDKGVAPGTAVLRQWLP<br>TGTLLVDSDLNDFVSDADSTLIGDCATVHT<br>ANKWDLIISDMYDPKTKNVTKENDSKEGFF<br>TYICGFIQQKLALGGSVAIKITEHSWNADL<br>YKLMGHFAWWTAFVTNVNASSSEAFLIGCN<br>YLGKPREQIDGYVMHANYIFWRNTNPIQLS<br>SYSLFDMSKFPLKLRGTAVMSLKEGQINDM<br>ILSLLSKGRLIIRENNRVVISSDVLVNN<br>nsp16	RGSRMNGLPSPTHSAHCSFYRTRTLQALSN<br>EKKAKKVRFYRNGDRYFKGIVYAVSSDRFR<br>SFDALLADLTRSLSDNINLPQGVRYIYTID<br>GSRKIGSMDELEEGESYVCSSDNFFKKVEY<br>TKNVNPNWSVNVKTSANMKAPQSLASSNSA<br>QARENKDFVRPKLVTIIRSGVKPRKAVRVL<br>LNKKTAHSFEQVLTDITEAIKLETGVVKKL<br>YTLDGKQVTCLHDFFGDDDVFIACGPEKFR<br>YAQDDFSLDENECRVMKGNPSATAGPKASP<br>TPQKTSAKSPGPMRRSKSPADSGNDQDA<br>DCX JHU04060
22	SSQAWQPGVAMPNLYKMQRMLLEKCDLQNY<br>GDSATLPKGIMMNVAKYTQLCQYLNTLTLA<br>VPYNMRVIHFGAGSDKGVAPGTAVLRQWLP<br>TGTLLVDSDLNDFVSDADSTLIGDCATVHT<br>ANKWDLIISDMYDPKTKNVTKENDSKEGFF<br>TYICGFIQQKLALGGSVAIKITEHSWNADL<br>YKLMGHFAWWTAFVTNVNASSSEAFLIGCN<br>YLGKPREQIDGYVMHANYIFWRNTNPIQLS<br>SYSLFDMSKFPLKLRGTAVMSLKEGQINDM<br>ILSLLSKGRLIIRENNRVVISSDVLVNN<br>nsp16	RGSRMNGLPSPTHSAHCSFYRTRTLQALSN<br>EKKAKKVRFYRNGDRYFKGIVYAVSSDRFR<br>SFDALLADLTRSLSDNINLPQGVRYIYTID<br>GSRKIGSMDELEEGESYVCSSDNFFKKVEY<br>TKNVNPNWSVNVKTSANMKAPQSLASSNSA<br>QARENKDFVRPKLVTIIRSGVKPRKAVRVL<br>LNKKTAHSFEQVLTDITEAIKLETGVVKKL<br>YTLDGKQVTCLHDFFGDDDVFIACGPEKFR<br>YAQDDFSLDENECRVMKGNPSATAGPKASP<br>TPQKTSAKSPGPMRRSKSPADSANGTSS<br>DCX JHU03009
23	SSQAWQPGVAMPNLYKMQRMLLEKCDLQNY<br>GDSATLPKGIMMNVAKYTQLCQYLNTLTLA<br>VPYNMRVIHFGAGSDKGVAPGTAVLRQWLP<br>TGTLLVDSDLNDFVSDADSTLIGDCATVHT<br>ANKWDLIISDMYDPKTKNVTKENDSKEGFF<br>TYICGFIQQKLALGGSVAIKITEHSWNADL<br>YKLMGHFAWWTAFVTNVNASSSEAFLIGCN	RGSRMNGLPSPTHSAHCSFYRTRTLQALSN<br>EKKAKKVRFYRNGDRYFKGIVYAVSSDRFR<br>SFDALLADLTRSLSDNINLPQGVRYIYTID<br>GSRKIGSMDELEEGESYVCSSDNFFKKVEY<br>TKNVNPNWSVNVKTSANMKAPQSLASSNSA<br>QARENKDFVRPKLVTIIRSGVKPRKAVRVL<br>LNKKTAHSFEQVLTDITEAIKLETGVVKKL

	YLGKPREQIDGYVMHANYIFWRNTNPIQLS<br>SYSLFDMSKFPLKLRGTAVMSLKEGQINDM<br>ILSLLSKGRLIIRENNRVVISSDVLVNN<br>nsp16	YTLDGKQVTCLHDFFGDDDVFIACGPEKFR<br>YAQDDFSLDENECRVMKGNPSATAGPKASP<br>TPQKTSAKSPGPMRRSKSPADSGNDQDA<br>DCX JHU04060
24	SSQAWQPGVAMPNLYKMQRMLLEKCDLQNY<br>GDSATLPKGIMMNVAKYTQLCQYLNTLTLA<br>VPYNMRVIHFGAGSDKGVAPGTAVLRQWLP<br>TGTLLVDSDLNDFVSDADSTLIGDCATVHT<br>ANKWDLIISDMYDPKTKNVTKENDSKEGFF<br>TYICGFIQQKLALGGSVAIKITEHSWNADL<br>YKLMGHFAWWTAFVTNVNASSSEAFLIGCN<br>YLGKPREQIDGYVMHANYIFWRNTNPIQLS<br>SYSLFDMSKFPLKLRGTAVMSLKEGQINDM<br>ILSLLSKGRLIIRENNRVVISSDVLVNN<br>nsp16	SQSQAEIRKQILGSSSSGKFFCLYTEEFAS<br>KDMTPLKPAEMQEANLTSMVLFMKRIDIAG<br>LGHCDFMNRPAPESLMQALEDLDYLAALDN<br>DGNLSEFGIIMSEFPLDPQLSKSILASCEF<br>DCVDEVLTIAAMVTAPNCFSHVPHGAEEAA<br>LTCWKTFLHPEGDHFTLISIYKAYQDTTLN<br>SSSEYCVEKWCRDYFLNCSALRMADVIRAE<br>LLEIIKRIELPYAEPAFGSKENTLNIKKAL<br>LSGYFMQIARDVDGSGNYLMLTHKQVAQLH<br>PLSGYSITKKMPEWVLFHKFSISENNYI<br>DHX32 JHU10488
25	SSQAWQPGVAMPNLYKMQRMLLEKCDLQNY<br>GDSATLPKGIMMNVAKYTQLCQYLNTLTLA<br>VPYNMRVIHFGAGSDKGVAPGTAVLRQWLP<br>TGTLLVDSDLNDFVSDADSTLIGDCATVHT<br>ANKWDLIISDMYDPKTKNVTKENDSKEGFF<br>TYICGFIQQKLALGGSVAIKITEHSWNADL<br>YKLMGHFAWWTAFVTNVNASSSEAFLIGCN<br>YLGKPREQIDGYVMHANYIFWRNTNPIQLS<br>SYSLFDMSKFPLKLRGTAVMSLKEGQINDM<br>ILSLLSKGRLIIRENNRVVISSDVLVNN<br>nsp16	MASRQPEVPALEASAPLGKMSLPIGIYRRA<br>VSYDDTLEDPAPMTPPPSDMGSVPWKPVIP<br>ERKYQHLAKVEEGEASLPSPAMTLSSAIDS<br>VDKVPVVKAKATHVIMNSLITKQTQESIQH<br>FERQAGLRDAGYTPHKGLTTEETKYLRVAE<br>ALHKLKLQSGEVTKEERQPASAQSTPSTTP<br>HSSPKQRPRGWFTSGSSTALPGPNPSTMDS<br>GSGDKDRNLSDKWSLFGPRSLQKYDSGSFA<br>TQAYRGAQKPSPLELIRAQANRMAEDPAAL<br>KPPKMDIPVMEGKKQPPRAHNLKPRDLN<br>KIAA1191 JHU07922
26	SSQAWQPGVAMPNLYKMQRMLLEKCDLQNY<br>GDSATLPKGIMMNVAKYTQLCQYLNTLTLA<br>VPYNMRVIHFGAGSDKGVAPGTAVLRQWLP<br>TGTLLVDSDLNDFVSDADSTLIGDCATVHT<br>ANKWDLIISDMYDPKTKNVTKENDSKEGFF<br>TYICGFIQQKLALGGSVAIKITEHSWNADL<br>YKLMGHFAWWTAFVTNVNASSSEAFLIGCN<br>YLGKPREQIDGYVMHANYIFWRNTNPIQLS<br>SYSLFDMSKFPLKLRGTAVMSLKEGQINDM<br>ILSLLSKGRLIIRENNRVVISSDVLVNN<br>nsp16	SADMSENSFYLKLDSVTAADTAVYYCAAGH<br>LVMGFGAHWGQGKLVSVSPASTKGPSVFPL<br>APCSRSTSESTAALGCLVKDYFPEPVTVSW<br>NSGALTSGVHTFPAVLQSSGLYSLSSVVTV<br>PSSSLGTKTYTCNVDHKPSNTKVDKRVESK<br>YGPPCPSCPAPEFLGGPSVFLFPPKPKDTL<br>MISRTPEVTCVVVDVSQEDPEVQFNWYVDG<br>VEVHNAKTKPREEQFNSTYRVVSVLTVLHQ<br>DWLNGKEYKCKVSNKGLPSSIEKTISKAKG<br>QPREPQVYTLPPSQEEMTKNQVSLTCLV<br>IGHG4 JHU03305
27	SSQAWQPGVAMPNLYKMQRMLLEKCDLQNY<br>GDSATLPKGIMMNVAKYTQLCQYLNTLTLA<br>VPYNMRVIHFGAGSDKGVAPGTAVLRQWLP<br>TGTLLVDSDLNDFVSDADSTLIGDCATVHT<br>ANKWDLIISDMYDPKTKNVTKENDSKEGFF<br>TYICGFIQQKLALGGSVAIKITEHSWNADL<br>YKLMGHFAWWTAFVTNVNASSSEAFLIGCN<br>YLGKPREQIDGYVMHANYIFWRNTNPIQLS<br>SYSLFDMSKFPLKLRGTAVMSLKEGQINDM<br>ILSLLSKGRLIIRENNRVVISSDVLVNN<br>nsp16	PSDGQAREKLALYVYEYLLHVGAQKSAQTF<br>LSEIRWEKNITLGEPPGFLHSWWCVFWDLY<br>CAAPERRDTCEHSSEAKAFHDYSAAAAPSP<br>VLGNIPPNDGMPGGPIPPGFFQGPPGSQPS<br>PHAQPPPHNPSSMMGPHSQPFMSPRYAGGP<br>RPPIRMGNQPPGGVPGTQPLLPNSMDPTRQ<br>QGHPNMGGSMQRMNPPRGMGPMGPGPQNYG<br>SGMRPPPNSLGPAMPGINMGPGAGRPWPNP<br>NSANSIPYSSSSPGTYVGPPGGGGPPGTPI<br>MPSPADSTNSSDNIYTMINPVPPGGSRS<br>SSBP3 JHU09872
28	SSQAWQPGVAMPNLYKMQRMLLEKCDLQNY<br>GDSATLPKGIMMNVAKYTQLCQYLNTLTLA<br>VPYNMRVIHFGAGSDKGVAPGTAVLRQWLP<br>TGTLLVDSDLNDFVSDADSTLIGDCATVHT<br>ANKWDLIISDMYDPKTKNVTKENDSKEGFF<br>TYICGFIQQKLALGGSVAIKITEHSWNADL	VPPGRAAGSGWAWRPVARDALLARAFHSCT<br>ELRGRFYLVGGLLAGGAREPSSDTVVFDPA<br>RGQAVRLGARGSPPRSHHDAAPVDGRWLCV<br>VGGWDGSRRLATVTALDTERGVWEAWTGTP<br>GDCPPAGLSSHTCTRISDRELQVAGREGGI<br>HTQRRYGSIYTLRLDPSARTYCYKQEGCHT

	YKLMGHFAWWTAFVTNVNASSSEAFLIGCN YLGKPREQIDGYVMHANYIFWRNTNPIQLS SYSLFDMSKFPLKLRGTAVMSLKEGQINDM ILSLLSKGRLIIRENNRVVISSDVLVNN nsp16	ASRSGHCAALLQTPGPHPGHQLLLFGGCNL AEPEVAGHWSHGKIKEEPPVAPHLMEQLAR LVSSGQGSQKGPHGLRHHSCSVVGPFAVLF GGETLTRTRDTICNDLYIYDTRTSPPLW KLHDC9 JHU15417
29	SSQAWQPGVAMPNLYKMQRMLLEKCDLQNY GDSATLPKGIMMNVAKYTQLCQYLNTLTLA VPYNMRVIHFGAGSDKGVAPGTAVLRQWLP TGTLLVDSDLNDFVSDADSTLIGDCATVHT ANKWDLIISDMYDPKTKNVTKENDSKEGFF TYICGFIQQKLALGGSVAIKITEHSWNADL YKLMGHFAWWTAFVTNVNASSSEAFLIGCN YLGKPREQIDGYVMHANYIFWRNTNPIQLS SYSLFDMSKFPLKLRGTAVMSLKEGQINDM ILSLLSKGRLIIRENNRVVISSDVLVNN nsp16	PSPKLGLPKSSESALKCRWHLAVTKTQPQA ACKPVRPSGAAEQKYVEKFLRVHGISLQET TRAETGMAYRNLGKSGLRVSCLGLGTWVTF GGQISDEVAERLMTIAYESGVNLFDTAEVY AAGKAEVILGSIIKKKGWRRSSLVITTKLY WGGKAETERGLSRKHIIEGLKGSLQRLQLE YVDVVFANRPDSNTPMEEIVRAMTHVINQG MAMYWGTSRWSAMEIMEAYSVARQFNMIPP VCEQAEYHLFQREKVEVQLPELYHKIGVGA MTWSPLACGIISGKYGNGVPESSRASLK KCNAB1 JHU13741
30	SQAWQPGVAMPNLYKMQRMLLEKCDLQNYG DSATLPKGIMMNVAKYTQLCQYLNTLTLAV PYNMRVIHFGAGSDKGVAPGTAVLRQWLPT GTLLVDSDLNDFVSDADSTLIGDCATVHTA NKWDLIISDMYDPKTKNVTKENDSKEGFFT YICGFIQQKLALGGSVAIKITEHSWNADLY KLMGHFAWWTAFVTNVNASSSEAFLIGCNY LGKPREQIDGYVMHANYIFWRNTNPIQLSS YSLFDMSKFPLKLRGTAVMSLKEGQINDMI LSLL nsp16	MPKVFLVKRRSLGVSVRSWDELPDEKRADT YIPVGLGRLLHDPPEDCRSDGGSSSGSGSS SAGEPGGAESSSSPHAPESETPEPGDAEGP DGHLATKQRPVARSKIKFTTGTCSDSVVHS CDLCGKGFRLQRMLNRHLKCHNQVKRHLCT FCGKGFNDTFDLKRHVRTHTGIRPYKCNVC NKAFTQRCSLESHLKKIHGVQQQYAYKQRR DKLYVCEDCGYTGPTQEDLYLHVNSAHPGS SFLKKTSKKLAALLQGKLTSAHQENTSLSE EEER OVOL2 JHU04191
31	SNCVADYSVLYNSASFSTFKCYGVSPTKLN DLCFTNVYADSFVIRGDEVRQIAPGQTGKI ADYNYKLPDDFTGCVIAWNSNNLDSKVGGN YNYLYRLFRKSNLKPFERDISTEIYQAGST PCNGVEGFNCYFPLQSYGFQPTNGVGYQPY RVVVLSFELLHAPATVCGPKKSTNLVKNKC VNFNFNGLTGTGVLTESNKKFLPFQQFGRD IADTTDAVRDPQTLEILDITPCSFGGVSVI TPGTNTSNQVAVLYQDVNCTEVPVAIHADQ LTPTWRVYSTGSNVFQTRAGCLIGAEHVNN SYECDIPIGAGICASYQTQTNSPRRARSVA SQSIIAYTMSLGAENSVAYSNNSIAIPTNF TISVTTEILPVSMTKTSVDCTMYICGDSTE CSNLLLQYGSFCTQLNRALTGIAVEQDKNT QEVFAQVKQIYKTPPIKDFGGFNFSQI Spike	MKGFIDDANYSVGLLDEGTNLGNVIDNYVY EHTLTGKNAFFVGDLGKIVKKHSQWQNVVA QIKPFYTVKCNSAPAVLEILAALGTGFACS SKNEMALVQELGVPPENIIYISPCKQVSQI KYAAKVGVNILTCDNEIELKKIARNHPNAK VLLHIATEDNIGGEEGNMKFGTTLKNCRHL LECAKELDVQIIGVKFHVSSACKESQVYVH ALSDARCVFDMAGEIGFTMNMLDIGGGFTG TEFQLEEVNHVISPLLDIYFPEGSGVKIIS EPGSYYVSSAFTLAVNIIAKKVVENDKFPS GVEKTGSDEPAFMYYMNDGVYGSFASKLSE DLNTIPEVHKKYKEDEPLFTSSLWGPSCDE LDQIVESCLLPELNVGDWLIFDNMGADSFH EPSAFNDFQRPAIYYMMSFSDWYEMQDAGI TSDSMMKNFFFVPSCIQLSQEDSFSAE AZIN1 JHU00873
32	TGVLTESNKKFLPFQQFGRDIADTTDAVRD PQTLEILDITPCSFGGVSVITPGTNTSNQV AVLYQDVNCTEVPVAIHADQLTPTWRVYST GSNVFQTRAGCLIGAEHVNNSYECDIPIGA GICASYQTQTNSPRRARSVASQSIIAYTMS LGAENSVAYSNNSIAIPTNFTISVTTEILP VSMTKTSVDCTMYICGDSTECSNLLLQYGS FCTQLNRALTGIAVEQDKNTQEVFAQVKQI YKTPPIKDFGGFNFSQILPDPSKPSKRSFI EDLLFNKVTLADAGFIKQYGDCLGDIAARD LICAQKFNGLTVLPPLLTDEMIAQYTSALL AGTI Spike	MASASSSRAGVALPFEKSQLTLKVVSAKPK VHNRQPRINSYVEVAVDGLPSETKKTGKRI GSSELLWNEIIILNVTAQSHLDLKVWSCHT LRNELLGTASVNLSNVLKNNGGKMENMQLT LNLQTENKGSVVSGGELTIFLDGPTVDLGN VPNGSALTDGSQLPSRDSSGTAVAPENRHQ PPSTNCFGGRSRTHRHSGASARTTPATGEQ SPGARSRHRQPVKNSGHSGLANGTVNDEPT TATDPEEPSVVGVTSPPAAPLSVTPNPNTT SLPAPATPAEGEEPSTSGTQQLPAAAQAPD ALPAGWEQRELPNGRVYYVDHNTKTTTWER PLPP WWP2 JHU04987

33	YQDVNCTEVPVAIHADQLTPTWRVYSTGSN VFQTRAGCLIGAEHVNNSYECDIPIGAGIC ASYQTQTNSPRRARSVASQSIIAYTMSLGA ENSVAYSNNSIAIPTNFTISVTTEILPVSM TKTSVDCTMYICGDSTECSNLLLQYGSFCT QLNRALTGIAVEQDKNTQEVFAQVKQIYKT PPIKDFGGFNFSQ Spike	MISRTPEVTCVVVDVSHEDPEVKFNWYVDG VEVHNAKTKPWEEQYNSTYHVVSVLTVVHQ NWLNGKEYKCKVSNKGLQAPIEKTISKTKG QPREPQVYTLPPSQKMTKNQVTLTCLVKGF YPSDIAVEWESNGQPENNYKTTPPMLDSNG SFFLYSKLTVDKSRWQQGNVFSCSVMHEGL QNHYTQKSLSLSP BC031259.1_frag JHU15432
34	DLCFTNVYADSFVIRGDEVRQIAPGQTGKI ADYNYKLPDDFTGCVIAWNSNNLDSKVGGN YNYLYRLFRKSNLKPFERDISTEIYQAGST PC Spike	MAQSGGEARPGPKTAVQIRVAIQEAEDVDE LEDEEEGAETRGAGDPARYLSPGWGSASEE EPSRGHRNRSSVNSRTMLASFIVSSAPSTA PS LARP6 JHU10982
35	QGFSALEPLVDLPIGINITRFQTLLALHRS YLTPGDSSSGWTAGAAAYYVGYLQPRTFLL KYNENGTITDAVDCALDPLSETKCTLKSFT VEKGIYQTSNFRVQPTESIVRFPNITNLCP FGEVFNATRFASVYAWNRKRISNCVADYSV LYNSASFSTFKCYGVSPTKLNDLCFTNVYA DSFVIRGDEVRQIAPGQTGKIADYNYKLPD DFTGCVIAWN Spike	MCPHLRPVPGTPCLPRWAALSPGDSVPSMP WLAETLNSSLGTVGLSPPLLPCLPLSVPGR LGAGQPEAETKAPHRRAQEVPALGTASSVA PARHQFPEGRSRPPPAAAVGLHSPAQPKAP RSWDSATPSEMLCPFSPVPLDAPSPQCSGD PWGTEQEGLDPVAQPKATRQPQPAQPPSSA WGLATGSSQWTPAPRGGRGTAPTAQQTCEL PECNLCDVFQ CPTP_frag JHU02353
36	SSSGWTAGAAAYYVGYLQPRTFLLKYNENG TITDAVDCALDPLSETKCTLKSFTVEKGIY QTSNFRVQPTESIVRFPNITNLCPFGEVFN ATRFASVYAWNRKRISNCVADYSVLYNSAS FSTFKCYGVSPTKLNDLCFTNVYADSFVIR GDEVRQIAPGQTGKIADYNYKLPDDFTGCV IAWNSNNLDSKVGGNYNYLYRLFRKSNLKP FERDISTEIYQAGSTPCNGVEGFNCYFPLQ SYGFQPTNGVGYQPYRVVVLSFELLHAPAT VCGPKKSTNLVKNKCVNFNFNGLTGTGVLT ESNKKFLPFQQFGRDIADTTDAVRDPQTLE ILDI Spike	MSTEGGFGGTSSSDAQQSLQSFWPRVMEEI RNLTVKDFRVQELPLARIKKIMKLDEDVKM ISAEAPVLFAKAAQIFITELTLRAWIHTED NKRRTLQRNDIAMAITKFDQFDFLIDIVPR DELKPPKRQEEVRQSVTPAEPVQYYFTLAQ QPTAVQVQGQQQGQQTTSSTTTIQPGQIII AQPQQGQTTPVTMQVGEGQQVQIVQAQPQG QAQQAQSGTGQTMQVMQQIITNTGEIQQIP VQLNAGQLQYIRLAQPVSGTQVVQGQIQTL ATNAQQITQTEVQQGQQQFSQFTDGQQLYQ IQQVTMPAGQDLAQPMFIQSANQPSDGQAP QVTG NFYC JHU04956
37	SPRRARSVASQSIIAYTMSLGAENSVAYSN NSIAIPTNFTISVTTEILPVSMTKTSVDCT MYICGDSTECSNLLLQYGSFCTQLNRALTG IAVEQDKNTQEVFAQVKQIYKTPPIKDFGG FNFSQILPDPSKPSKRSFIEDLLFNKVTLA DAGFIKQYGDCLGDIAARDLICAQKFNGLT VLPPLLTDEMIAQYTSALLAGTITSGWTFG AGAALQIPFA Spike	MAGTGLLALRTLPGPSWVRGSGPSVLSRLQ DAAVVRPGFLSTAEEETLSRELEPELRRRR YEYDHWDAAIHGFRETEKSRWSEASRAILQ RVQAAAFGPGQTLLSSVHVLDLEARGYIKP HVDSIKFCGATIAGLSLLSPSVMRLVHTQE PGEWLELLLEPGSLYILRGSARYDFSHEIL RDEESFFGERRIPRGRRISVICRSLPEGMG PGESGQPPPA ALKBH7 JHU04132
38	GFSALEPLVDLPIGINITRFQTLLALHRSY LTPGDSSSGWTAGAAAYYVGYLQPRTFLLK YNENGTITDAVDCALDPLSETKCTLKSFTV EKGIYQTSNFRVQPTESIVRFPNITNLCPF GEVFNATRFASVYAWNRKRISNCVADYSVL YNSASFSTFKCYGVSPTKLNDLCFTNVYAD SFVIRGDEVRQIAPGQTGKIADYNYKLPDD FTGCVIAWNSNNLDSKVGGNYNYLYRLFRK SNLKPFERDISTEIYQAGSTPCNGVEGFNC YFPLQSYGFQPTNGVGYQPYRVVVLSFELL HAPATVCGPKKSTNLVKNKCVNFNFNGLTG TGVLTESNKKFLPFQQFGRDIADTTDAVRD PQTLEILDITPCSFGGVSVITPGTNTSNQV AVLYQDVNCTEVPVAIHADQLTPTWRVYST	MPGFLVRILLLLLVLLLLGPTRGLRNATQR MFEIDYSRDSFLKDGQPFRYISGSIHYSRV PRFYWKDRLLKMKMAGLNAIQTYVPWNFHE PWPGQYQFSEDHDVEYFLRLAHELGLLVIL RPGPYICAEWEMGGLPAWLLEKESILLRSS DPDYLAAVDKWLGVLLPKMKPLLYQNGGPV ITVQVENEYGSYFACDFDYLRFLQKRFRHH LGDDVVLFTTDGAHKTFLKCGALQGLYTTV DFGTGSNITDAFLSQRKCEPKGPLINSEFY TGWLDHWGQPHSTIKTEAVASSLYDILARG ASVNLYMFIGGTNFAYWNGANSPYAAQPTS YDYDAPLSEAGDLTEKYFALRNIIQKFEKV PEGPIPPSTPKFAYGKVTLEKLKTVGAALD ILCPSGPIKSLYPLTFIQVKQHYGFVLYRT

	GSNVFQTRAGCLIGAEHVNNSYECDIPIGAGICASYQTQTNSPRRARSVASQSIIAYTMSLGAENSVAYSNNSIAIPTNFTISVTTEILPVSMTKTSVDCTMYICGDSTECSNLLLQYGSFCTQLNRALTGIAVEQDKNTQEVFAQVKQIYKTPPIKDFGGFNFSQILPDPSKPSKRSFIEDLLFNKVTLADAGFIKQYGDCLGDIAARDLICAQKFNGLTVLPPLLTDEMIAQYTSALLAGTITSGWTFGAGAAL Spike	TLPQDCSNPAPLSSPLNGVHDRAYVAVDGIPQGVLERNNVITLNITGKAGATLDLLVENMGRVNYGAYINDFKGLVSNLTLSSNILTDWTIFPLDTEDAVRSHLGGWGHRDSGHHDEAWAHNSSNYTLPAFYMGNFSIPSGIPDLPQDTFIQFPGWTKGQVWINGFNLGRYWPARGPQLTLFVPQHILMTSAPNTITVLELEWAPCSSDDPELCAVTFVDRPVIGSSVTYDHPSKPVEKRLMPPPPQKNKDSWLDH GLB1 JHU21299
39	QLSSNFGAISSVLNDILSRLDKVEAEVQIDRLITGRLQSLQTYVTQQLIRAAEIRASANLAATKMSECVLGQSKRVDFCGKGYHLMSFPQSAPHGVVFLHVTYVPAQEKNFTTAPAI Spike	MPGAEDDVVTPGTSEDRYKSGLTTLVATSVNSVTGIRIEDLPTSESTVHAQEQSPSATASNVATSHSTEKVDGDTQTTVEKDGLSTVTLVGIIVGVLLAIGFIGAIIVVVMRKMSGR PDPN JHU14405
40	LLLQYGSFCTQLNRALTGIAVEQDKNTQEVFAQVKQIYKTPPIKDFGGFNFSQILPDPSKPSKRSFIEDLLFNKVTLADAGFIKQYGDCLGDIAARDLICAQKFNGLTVLPPLLTDEMIAQYTSALLAGTITSGWTFGAGAALQIPFAMQMAYRFNGIGVTQNVLYENQKLIANQFNSAIGKIQDSLSSTASALGKLQDVVNQNAQALNTLVKQLSSNFGAISSVLNDILSRLDKVEAEVQIDRLITGRLQSLQTYVTQQLIRAAEIRASANLAATKMSECVLGQSKRVDFCGKGYHLMSFPQSAPHGVVFLHVTYVPAQEKNFTTAP Spike	MAAQYGSMSFNPSTPGASYGPGRQEPRNSQLRIVLVGKTGAGKSATGNSILGRKVFHSGTAAKSITKKCEKRSSSWKETELVVVDTPGIFDTEVPNAETSKEIIRCILLTSPGPHALLLVVPLGRYTEEEHKATEKILKMFGERARSFMILIFTRKDDLGDTNLHDYLREAPEDIQDLMDIFGDRYCALNNKATGAEQEAQRAQLLGLIQRVVRENKEGCYTNRMYQRAEEEIQKQTQAMQELHRVELEREKARIREEYEEKIRKLEDKVEQEKRKKQMEKKLAEQEAHYAVRQQRARTEVESKDGILELIMTALQIASFILLRLFAE GIMAP4 JHU04931
41	MFHLVDFQVTIAEILLIIMRTFKVSIWNLDYIINLIIKNLSKSLTENKYSQLDEEQPMEID orf6	DQLWEDSVLTVKFPKLMVPRFSFAAPSSEDDVFIPTVREVQCPEANIDTALCKESPGLWGA AHNAK2 JHU07968
42	MFHLVDFQVTIAEILLIIMRTFKVSIWNLDYIINLIIKNLSKSLTENKYSQLDEEQPMEID orf6	VFSDYYDLGYNMRSNLFRGAAEETKSLMKASYTPEVIEKSVRDLEHWHGRKTDDLGRWHQK TEX33 JHU20055
43	MFHLVDFQVTIAEILLIIMRTFKVSIWNLDYIINLIIKNLSKSLTENKYSQLDEEQPMEID orf6	DVALKNFAKYFLHQSHEEREHAEKLMKLQNQRGGRIFLQDIKEPDCDDWESGQNAMECALH FTH1 JHU15414
44	MFHLVDFQVTIAEILLIIMRTFKVSIWNLDYIINLIIKNLSKSLTENKYSQLDEEQPMEID orf6	AEKSSKQKVDLQSLPTRAYLDQTVVPILLQGLAVLAKERPPNPIEFLASYLLKNKAQFEDR DPY30 JHU03411
45	MFHLVDFQVTIAEILLIIMRTFKVSIWNLDYIINLIIKNLSKSLTENKYSQLDEEQPMEID orf6	MQASEDLLKEHYVDLKDRPFFAGLVKYMHSGPVVAMVWEGLNVVKTGRVMLGETNPADSKP NME1 JHU00057
46	MFHLVDFQVTIAEILLIIMRTFKVSIWNLDYIINLIIKNLSKSLTENKYSQLDEEQPMEID orf6	VKHSDVLFLAVKPHIIPFILDEIGADVQARHIVVSCAAGVTISSVEKKLMAFQPAPKVIRC PYCR2 JHU04471
47	MFHLVDFQVTIAEILLIIMRTFKVSIWNLDYIINLIIKNLSKSLTENKYSQLDEEQPMEID orf6	GSGCFSSGGGGFSGQAVQCQSYGGVSSGGSSGGGSGCFSSGGGGGSVCGYSGGGSGGGSGC LOR JHU15534
48	MFHLVDFQVTIAEILLIIMRTFKVSIWNLDYIINLIIKNLSKSLTENKYSQLDEEQPMEID orf6	EFQKILWKEREMRTALEKEIERLESALSLWKWKYEELKESKPKNVKEFDILLGQHNDEMQE CCDC102B_frag JHU15882
49	MFHLVDFQVTIAEILLIIMRTFKVSIWNLDYIINLIIKNLSKSLTENKYSQLDEEQPMEID orf6	AEEEELRAELTKVEEEIVTLRQVLAAKERHCGELKRRLGLSTLGELKQNLSRSWHDVQVSS TPD52L2 JHU04498

50	MFHLVDFQVTIAEILLIIMRTFKVSIWNLDYIINLIIKNLSKSLTENKYSQLDEEQPMEID orf6	VLEPEEDFEQFLLPVINEMREDIASLIREHGRAYLRTRSKLWEMDNMLIQIKTQVEASEES MRFAP1L1 JHU02929
51	MKFLVFLGIITTVAAFHQECSLQSCTQHQPYVVDDPCPIHFYSKWYIRVGARKSAPLIELCVDEAGSKSPIQYIDIGNYTVSCLPFTINCQEPKLGSLVVRCSFYEDFLEYHDVRVVLDFI orf8	QFVEGVRMEEIVEGCTGALHILARDVHNRIVIRGLNTIPLFVQLLYSPIENIQRVAAGVLCELAQDKEAAEAIEAEGATAPLTELLHSRNEGVATYAAAVLFRMSEDKPQDYKKRLSVELT CTNNB1 JHU07025
52	MKFLVFLGIITTVAAFHQECSLQSCTQHQPYVVDDPCPIHFYSKWYIRVGARKSAPLIELCVDEAGSKSPIQYIDIGNYTVSCLPFTINCQEPKLGSLVVRCSFYEDFLEYHDVRVVLDFI orf8	EKMATVWDEAEQDGIGEEVLKMSTEEIIQRTRLLDSEIKIMKSEVLRVTHELQAMKDKIKENSEKIKVNKTLPYLVSNVIELLDVDPNDQEEDGANIDLDSQRKGKCAVIKTSTRQTYFLP PSMC3 JHU13762
53	MKFLVFLGIITTVAAFHQECSLQSCTQHQPYVVDDPCPIHFYSKWYIRVGARKSAPLIELCVDEAGSKSPIQYIDIGNYTVSCLPFTINCQEPKLGSLVVRCSFYEDFLEYHDVRVVLDFI orf8	LPLESDAVECLNYQHYKGSDFDCELRLLIHQSLAGGIIGVKGAKIKELRENTQTTIKLFQECCPHSTDRVVLIGGKPDRVVECIKIILDLISESPIKGRAQPYDPNFYDETYDYGGFTMMF HNRNPK JHU02733
54	MKFLVFLGIITTVAAFHQECSLQSCTQHQPYVVDDPCPIHFYSKWYIRVGARKSAPLIELCVDEAGSKSPIQYIDIGNYTVSCLPFTINCQEPKLGSLVVRCSFYEDFLEYHDVRVVLDFI orf8	GLHLASLSHAILEALAAPDRAPFALLALRCACPEDARASPLPNEAWVALRRRHPGLAVELELEPALPAESVTRVLQPAVPVAALRLNLSGDTVGPVRFAAHHYAATLCALEVRAAASAELN FBXL8 JHU19904
55	MKFLVFLGIITTVAAFHQECSLQSCTQHQPYVVDDPCPIHFYSKWYIRVGARKSAPLIELCVDEAGSKSPIQYIDIGNYTVSCLPFTINCQEPKLGSLVVRCSFYEDFLEYHDVRVVLDFI orf8	ATQLGATLLRLRGATLAAPGAAEGARLLQAAFAAARCRPPSVLLISELEALLPARDDGAAAGGALQVPLLACLDGGCGAGADGVLVVGTTSRPAALDEATRRRFSLRFYVALPDSPARGQI FIGNL2 JHU17712
56	MKFLVFLGIITTVAAFHQECSLQSCTQHQPYVVDDPCPIHFYSKWYIRVGARKSAPLIELCVDEAGSKSPIQYIDIGNYTVSCLPFTINCQEPKLGSLVVRCSFYEDFLEYHDVRVVLDFI orf8	LIRHQRIHTGEKPFECKECGKGFSQNTSLTQHQRIHTGEKPYTCKECGKSFTRNPALLRHQRMHTGEKPYECKDCGKGFMWNSDLSQHQRVHTGDKPHECTDCGKSFFCKAHLIRHQRIHT ZNF662 JHU29296
57	MKFLVFLGIITTVAAFHQECSLQSCTQHQPYVVDDPCPIHFYSKWYIRVGARKSAPLIELCVDEAGSKSPIQYIDIGNYTVSCLPFTINCQEPKLGSLVVRCSFYEDFLEYHDVRVVLDFI orf8	LTQHQRVHTGERPYECDACGKAFSQSTHLTQHQRIHTGEKPYKCDACGRAFSDCSALIRHLRIHSGEKPYQCKVCPKAFAQSSSLIEHQRIHTGEKPYKCSDCGKAFSRSSALMVHLRIHI ZSCAN22 JHU19974
58	MKFLVFLGIITTVAAFHQECSLQSCTQHQPYVVDDPCPIHFYSKWYIRVGARKSAPLIELCVDEAGSKSPIQYIDIGNYTVSCLPFTINCQEPKLGSLVVRCSFYEDFLEYHDVRVVLDFI orf8	ALKSLSLNSPVQPLENQCKTETQESQAFQERDGRMVAGKVLMAKQEIVECVASAAMISPGKLPGETHSQRIAEEALGGLDNSKKQKGNAAGNKISQLPSQDRHFSLATFNRRIPTEHSVLE ZSCAN30 JHU29179
59	MKFLVFLGIITTVAAFHQECSLQSCTQHQPYVVDDPCPIHFYSKWYIRVGARKSAPLIELCVDEAGSKSPIQYIDIGNYTVSCLPFTINCQEPKLGSLVVRCSFYEDFLEYHDVRVVLDFI orf8	LTQHQRIHTGEKPYECKECGKAFRRSAAYLQHQRLHTGEKLYKCKECWKAFGCRSLFIVHQRIHTGEKPYQCKECGKAFTQKIASIQHQRVHTGEKPYECKVCGKAFKWYGSFVQHQKLHP ZNF621 JHU11225
60	MKFLVFLGIITTVAAFHQECSLQSCTQHQPYVVDDPCPIHFYSKWYIRVGARKSAPLIELCVDEAGSKSPIQYIDIGNYTVSCLPFTINCQEPKLGSLVVRCSFYEDFLEYHDVRVVLDFI orf8	LIQHQRIHTGEKPYECKECGKAFSSSSVFLQHQRFHTGEKLYECNECWKTFSCSSSFTVHQRMHTGEKPYECKECGKRLSSNTALTQHQRIHTGEKPFECKECGKAFNQKITLIQHQRVHT ZNF620 JHU24458

61	MGYINVFAFPFTIYSLLLCRMNSRNYIAQV DVVNFNLT orf10	KMQDKTQMQEKAKEIYMTFLSSKASSQVNV EGQSRLNE RGS10 JHU11967
62	MGYINVFAFPFTIYSLLLCRMNSRNYIAQV DVVNFNLT orf10	DLNLVQALRQFLWSFRLPGEAQKIDRMMEA FAQRYCQC CYTH1 JHU05740
63	MGYINVFAFPFTIYSLLLCRMNSRNYIAQV DVVNFNLT orf10	EKGCHFLHILACARLSIRPGLSEAVLQQVL ELLEDQSD OBFC1 JHU09466
64	MGYINVFAFPFTIYSLLLCRMNSRNYIAQV DVVNFNLT orf10	KVALQKALLYYESIHGRPVTKNERQVMKPL YDRYRLVK FAM13A JHU15982
65	MGYINVFAFPFTIYSLLLCRMNSRNYIAQV DVVNFNLT orf10	IKTITSSLKFYLRNLSEPVMTYRLHKELVS AAKSDNLD OPHN1 JHU13650
66	MGYINVFAFPFTIYSLLLCRMNSRNYIAQV DVVNFNLT orf10	QAGFTLHSAIYAARPDVKCVVHIHTPAGAA VSAMKCGL ADD1 JHU13212
67	MGYINVFAFPFTIYSLLLCRMNSRNYIAQV DVVNFNLT orf10	SPQPLLQDITYLRIPVADTPEVPIKKHFKE CINFIHCC DUSP15 JHU12281
68	MGYINVFAFPFTIYSLLLCRMNSRNYIAQV DVVNFNLT orf10	NVAHGLAWSYYIGYLRLILPELQARIRTYN QHYNNLLR TMEM173 JHU05940
69	MGYINVFAFPFTIYSLLLCRMNSRNYIAQV DVVNFNLT orf10	KMQDKTQMQEKAKEIYMTFLSSKASSQVNV EGQSRLNE RGS10 JHU08427
70	MGYINVFAFPFTIYSLLLCRMNSRNYIAQV DVVNFNLT orf10	ESSSSMPLSFPSLLPSVPHNTNPSPPLMSY ITSQEMKC C14orf119 JHU04523

Supplementary Table S3.

Index	Thermodynamically Aligned Region of Protein 1 (Virus Protein)	Thermodynamically Aligned Region of Protein 2 (Human Protein)
1	MSDNGPQNQRNAPRITFGGPSDSTGSNQNG<br>ERSGARSKQRRPQGLPNNTASWFTALTQHG<br>KEDLKFPRGQGVPINTNSSPDDQIGYYRRA<br>TRRIRGGDGKMKDLSPRWYFYYLGTGPEAG<br>LPYGANKDGIIWVATEGALNTPKDHIGTRN<br>PANNAAIVLQLPQGTTLPKGFYAEGSRGGS<br>QASSRSSSRSRNSSRNSTPGSSRGTSPARM<br>AGNGGDAALALLLLDRLNQLESKMSGKGQQ<br>QQGQTVTKKSAAEASKKPRQKRTATKAYNV<br>TQAFGRRGPEQTQGNFGDQELIRQGTDYKH<br>WPQIAQFAPSASAFFGMSRIGMEVTPSGTW<br>LTYTGAIKLDDKDPNFKDQVILLNKHIDAY<br>KTFPPTEPKKDKKKKADETQALPQRQKKQQ<br>TVTLLPAADLDDFSKQLQQSMSSADSTQA<br>orf9	SPGHILCCECGVPISPNPANICVACLRSKV<br>DISQGIPKQVSISFCKQCQRYFQPPGTWIQ<br>CALESRELLALCLKKIKAPLSKVRLVDAGF<br>VWTEPHSKRLKVKLTIQKEVMNGAILQQVF<br>VVDYVVQSQMCGDCHRVEAKDFWKAVIQVR<br>QKTLHKKTFYYLEQLILKYGMHQNTLRIKE<br>IHDGLDFYYSSKQHAQKMVEFLQCTVPCRY<br>KASQRLISQDIHSNTYNYKSTFSVEIVPIC<br>KDNVVCLSPKLAQSLGNMNQICVCIRVTSA<br>IHLIDPNTLQVADIDGSTFWSHPFNSLCHP<br>KQLEEFIVMECSIVQDIKRAAGAGMISKKH<br>TLGEVWVQKTSEMNTDKQYFCRTHLGHLLN<br>PGDLVLGFDLANCNLNDEHVNKMNSDRVPD<br>VVLIKKSYDRTKRQRRRNWKLKELARERE<br>NMD3 JHU03424 { 0.989, 0.985, 0.981 }
2	MSDNGPQNQRNAPRITFGGPSDSTGSNQNG<br>ERSGARSKQRRPQGLPNNTASWFTALTQHG<br>KEDLKFPRGQGVPINTNSSPDDQIGYYRRA<br>TRRIRGGDGKMKDLSPRWYFYYLGTGPEAG<br>LPYGANKDGIIWVATEGALNTPKDHIGTRN<br>PANNAAIVLQLPQGTTLPKGFYAEGSRGGS<br>QASSRSSSRSRNSSRNSTPGSSRGTSPARM<br>AGNGGDAALALLLLDRLNQLESKMSGKGQQ<br>QQGQTVTKKSAAEASKKPRQKRTATKAYNV<br>TQAFGRRGPEQTQGNFGDQELIRQGTDYKH<br>WPQIAQFAPSASAFFGMSRIGMEVTPSGTW<br>LTYTGAIKLDDKDPNFKDQVILLNKHIDAY<br>KTFPPTEPKKDKKKKADETQALPQRQKKQQ<br>TVTLLPAADLDDFSKQLQQSMSSADSTQA<br>orf9	TPPLETLSFHGDEEIIEVVELDPGPPDPDD<br>LAQEMEDVDFEEEEEEGNEEGWVLEPQEG<br>VVGSMEGPDDSEVTFALHSASVFCVSLDPK<br>TNTLAVTGGEDDKAFVWRLSDGELLFECAG<br>HKDSVTCAGFSHDSTLVATGDMSGLLKVWQ<br>VDTKEEVWSFEAGDLEWMEWHPRAPVLLAG<br>TADGNTWMWKVPNGDCKTFQGPNCPATCGR<br>VLPDGKRAVVGYEDGTIRIWDLKQGSPIHV<br>LKGTEGHQGPLTCVAANQDGSLILTGSVDC<br>QAKLVSATTGKVVGVFRPETVASQPSLGEG<br>EESESNSVESLGFCSVMPLAAVGYLDGTLA<br>IYDLATQTLRHQCQHQSGIVQLLWEAGTAV<br>VYTCSLDGIVRLWDARTGRLLTDYRGHTAE<br>ILDFALSKDASLVVTTSGDHKAKVFCVQR<br>AAMP JHU13499 { 0.988, 0.987, 0.987 }
3	DNGPQNQRNAPRITFGGPSDSTGSNQNGER<br>SGARSKQRRPQGLPNNTASWFTALTQHGKE<br>DLKFPRGQGVPINTNSSPDDQIGYYRRATR<br>RIRGGDGKMKDLSPRWYFYYLGTGPEAGLP<br>YGANKDGIIWVATEGALNTPKDHIGTRNPA<br>NNAAIVLQLPQGTTLPKGFYAEGSRGGSQA<br>SSRSSSRSRNSSRNSTPGSSRGTSPARMAG<br>NGGDAALALLLLDRLNQLESKMSGKGQQQQ<br>GQTVTKKSAAEASKKPRQKRTATKAYNVTQ<br>AFGRRGPEQTQGNFGDQELIRQGTDYKHWP<br>QIAQFAPSASAFFGMSRIGMEVTPSGTWLT<br>YTGAIKLDDKDPNFKDQVILLNKHIDAYKT<br>FPPTEPKKDKKKKADETQALPQRQKKQQTV<br>TLLPA orf9	MESRCYGCAVKFTLFKKEYGCKNCGRAFCS<br>GCLSFSAAVPRTGNTQQKVCKQCHEVLTRG<br>SSANASKWSPPQNYKKRVAALEAKQKPSTS<br>QSQGLTRQDQMIAERLARLRQENKPKLVPS<br>QAEIEARLAALKDERQGSIPSTQEMEARLA<br>ALQGRVLPSQTPQPAHHTPDTRTQAQQTQD<br>LLTQLAAEVAIDESWKGGGPAASLQNDLNQ<br>GGPGSTNSKRQANWSLEEEKSRLLAEAALE<br>LREENTRQERILALAKRLAMLRGQDPERVT<br>LQDYRLPDSDDDEDEETAIQRVLQQLTEEA<br>ALDEASGFNIPAEQASRPWTQPRGAEPEAQ<br>DVDPRPEAEEEELPWCCICNEDATLRCAGC<br>DGDLFCARCFREGHDAFELKEHQTSAYSPP<br>RAGQE ZFYVE19 JHU04797 { 0.990, 0.984, 0.981 }
4	SSRSSSRSRNSSRNSTPGSSRGTSPARMAG<br>NGGDAALALLLLDRLNQLESKMSGKGQQQQ	MLSCCLEPAAWDCGAEGRDGREEMVLGWKR<br>DRGTTPRTLPASPSAGTPLWGAGHVLGDAG

	GQTVTKKSAAEASKKPRQKRTATKAYNVTQ AFGRRGPEQTQGNFGDQELIRQGTDYKHWP QIAQFAPSASAFFGMSRIGME orf9	ESPLPSHPCPNRWSCCWSRKSCGVLAGWRC GSEAAPLTQPPEVGGLRQDPGVRGNPSLPA PPQPVMRRLTFGPGPAPASDR YIF1B JHU13458 { 0.983, 0.979, 0.976 }
5	GGSQASSRSSSRSRNSSRNSTPGSSRGTSP ARMAGNGGDAALALLLLDRLNQLESKMSGK GQQQQGQTVTKKSAAEASKKPRQKRTATKA YNVTQAFGRRGPEQTQGNFGDQELIRQGTD YKHWPQIAQFAPSASAFFGMSRIGMEVTPS GTWLTYTGAIKLDDKDPNFKDQVILLNKHI orf9	MIRAFSFPVSPERGRLRGWLEGSLAGLCEL HWLRERQEYRVQQALRLAQPGMGGAEAEDE EDADEDEDAAAARRAAALEEQLEALPGLV WDLGQQLGDLSLESGGLEQESGRSSGFYED PSSTGGPDSPPSTFCGDSGFSGSSSYGRLG PSEPRGIYASERPKSLGKVGEGGALGPGKG DACT3 JHU03094 { 0.989, 0.981, 0.975 }
6	MSDNGPQNQRNAPRITFGGPSDSTGSNQNG ERSGARSKQRRPQGLPNNTASWFTALTQHG KEDLKFPRGQGVPINTNSSPDDQIGYYRRA TRRIRGGDGKMKDLSPRWYFYYLGTGPEAG LPYGANKDGIIWVATEGALNTPKDHIGTRN PANNAAIVLQLPQGTTLPKGFYAEGSRGGS QASSRSSSRSRNSSRNSTPGSSRGTSPARM AGNGGDAALALLLLDRLNQLESKMSGKGQQ QQGQTVTKKSAAEASKKPRQKRTATKAYNV TQAFGRRGPEQTQGNFGDQELIRQGTDYKH WPQIAQFAPSASAFFGMSRIGMEVTPSGTW LTYTGAIKLDDKDPNFKDQVILLNKHIDAY KTFPPTEPKKDKKKKADETQALPQRQKKQQ TVTLLPAADLDDFSKQLQQSMSSADSTQA orf9	AKTEPKDAGSEKSTGVVRLNTVRQIIEQDK HALLDVTPKAVDLLNYTQWFPIVIFFNPDS RQGVKTMRQRLNPTSNKSSRKLFDQANKLK KTCAHLFTATINLNSANDSWFGSLKDTIQH QQGEAVWVSEGKMEGMDDDPEDRMSYLTAM GADYLSCDSRLISDFEDTDGEGGAYTDNEL DEPAEEPLVSSITRSSEPVQHEESIRKPSP EPRAQMRRAASSDQLRDNSPPPAFKPEPPK AKTQNKEESYDFSKSYEYKSNPSAVAGNET PGASTKGYPPPVAAKPTFGRSILKPSTPIP PQEGEEVGESSEEQDNAPKSVLGKVKIFEK MDHKARLQRMQELQEAQNARIEIAQKHPDI YAVPIKTHKPDPGTPQHTSSRPPEPQKAPS RPYQDTRGSYGSDAEEEEYRQQLSEHSKR TJP2 JHU18226 { 0.987, 0.985, 0.985 }
7	MSDNGPQNQRNAPRITFGGPSDSTGSNQNG ERSGARSKQRRPQGLPNNTASWFTALTQHG KEDLKFPRGQGVPINTNSSPDDQIGYYRRA TRRIRGGDGKMKDLSPRWYFYYLGTGPEAG LPYGANKDGIIWVATEGALNTPKDHIGTRN PANNAAIVLQLPQGTTLPKGFYAEGSRGGS QASSRSSSRSRNSSRNSTPGSSRGTSPARM AGNGGDAALALLLLDRLNQLESKMSGKGQQ QQGQTVTKKSAAEASKKPRQKRTATKAYNV TQAFGRRGPEQTQGNFGDQELIRQGTDYKH WPQIAQFAPSASAFFGMSRIGMEVTPSGTW LTYTGAIKLDDKDPNFKDQVILLNKHIDAY KTFPPTEPKKDKKKKADETQALPQRQKKQQ TVTLLPAADLDDFSKQLQQSMSSADSTQA orf9	SGDEENAGTEEVKNEINGNWISAYSINEAR INAKAKRRLRKNSSRDSGRGDSVSDSGSDA LRSGLTVPTSPKGRLLDRRSRSGKGRGLPK KGGAGGKGVWGTPGQVYDVEEVDVKDPNYD DDQENCVYETVVLPLDERAFEKTLTPIIQE YFEHGDTNEVAEMLRDLNLGEMKSGVPVLA VSLALEGKASHREMTSKLLSDLCGTVMSTT DVEKSFDKLLKDLPELALDTPRAPQLVGQF IARAVGDGILCNTYIDSYKGTVDCVQARAA LDKATVLLSMSKGGKRKDSVWGSGGGQQSV NHLVKEIDMLLKEYLLSGDISEAEHCLKEL EVPHFHHELVYEAIIMVLESTGESTFKMIL DLLKSLWKSSTITVDQMKRGYERIYNEIPD INLDVPHSYSVLERFVEECFQAGIISKQL PDCD4 JHU03330 { 0.989, 0.987, 0.981 }
8	MSDNGPQNQRNAPRITFGGPSDSTGSNQNG ERSGARSKQRRPQGLPNNTASWFTALTQHG KEDLKFPRGQGVPINTNSSPDDQIGYYRRA TRRIRGGDGKMKDLSPRWYFYYLGTGPEAG LPYGANKDGIIWVATEGALNTPKDHIGTRN PANNAAIVLQLPQGTTLPKGFYAEGSRGGS QASSRSSSRSRNSSRNSTPGSSRGTSPARM AGNGGDAALALLLLDRLNQLESKMSGKGQQ QQGQTVTKKSAAEASKKPRQKRTATKAYNV	AKTEPKDAGSEKSTGVVRLNTVRQIIEQDK HALLDVTPKAVDLLNYTQWFPIVIFFNPDS RQGVKTMRQRLNPTSNKSSRKLFDQANKLK KTCAHLFTATINLNSANDSWFGSLKDTIQH QQGEAVWVSEGKMEGMDDDPEDRMSYLTAM GADYLSCDSRLISDFEDTDGEGGAYTDNEL DEPAEEPLVSSITRSSEPVQHEESIRKPSP EPRAQMRRAASSDQLRDNSPPPAFKPEPPK AKTQNKEESYDFSKSYEYKSNPSAVAGNET

	TQAFGRRGPEQTQGNFGDQELIRQGTDYKH<br>WPQIAQFAPSASAFFGMSRIGMEVTPSGTW<br>LTYTGAIKLDDKDPNFKDQVILLNKHIDAY<br>KTFPPTEPKKDKKKKADETQALPQRQKKQQ<br>TVTLLPAADLDDFSKQLQQSMSSADSTQA<br>orf9	PGASTKGYPPPVAAKPTFGRSILKPSTPIP<br>PQEGEEVGESSEEQDNAPKSVLGKVKIFEK<br>MDHKARLQRMQELQEAQNARIEIAQKHPDI<br>YAVPIKTHKPDPGTPQHTSSRPPEPQKAPS<br>RPYQDTRGSYGSDAEEEEYRQQLSEHSKR<br>TJP2 JHU18226 { 0.987, 0.985,<br>0.985 }
9	MSDNGPQNQRNAPRITFGGPSDSTGSNQNG<br>ERSGARSKQRRPQGLPNNTASWFTALTQHG<br>KEDLKFPRGQGVPINTNSSPDDQIGYYRRA<br>TRRIRGGDGKMKDLSPRWYFYYLGTGPEAG<br>LPYGANKDGIIWVATEGALNTPKDHIGTRN<br>PANNAAIVLQLPQGTTLPKGFYAEGSRGGS<br>QASSRSSSRSRNSSRNSTPGSSRGTSPARM<br>AGNGGDAALALLLLDRLNQLESKMSGKGQQ<br>QQGQTVTKKSAAEASKKPRQKRTATKAYNV<br>TQAFGRRGPEQTQGNFGDQELIRQGTDYKH<br>WPQIAQFAPSASAFFGMSRIGMEVTPSGTW<br>LTYTGAIKLDDKDPNFKDQVILLNKHIDAY<br>KTFPPTEPKKDKKKKADETQALPQRQKKQQ<br>TVTLLPAADLDDFSKQLQQSMSSADSTQA<br>orf9	EGEKEGNNESHMVSPPEKDDGQKGEEAVGS<br>TEHPEEVTTQAEAAIEEGEVETEGEAAVEG<br>EEEAVSYGDAESEEEYYYTETSSPEGQISA<br>ADTTYPYFSPPQELPGEEAYDSVSGEAGLQ<br>GFQQEATGPPESRERRVTSPEPSHGVLGPS<br>EQMGQVTSGPAVGRLTGSTEEPQGQVLPMG<br>VQHRFRLSHGSDIESSDLEEFVSQEPVIPP<br>GVPDAHPREGDLPVFQDQIQQPSTEEGAMA<br>ERVESEGSDEEAEDEGSQLVVLDPDHPLMV<br>RFQAALKNYLNRQIEKLKLDLQELVVATKQ<br>SRAQRQELGVNLYEVQQHLVHLQKLLEKSH<br>DRHAMASSERRQKEEELQAARALYTKTCAA<br>ANEERKKLAALQTEMENLALHLFYMQNIDQ<br>DMRDDIRVMTQVVKKAETERIRAEIEKKK<br>CCDC40 JHU10191 { 0.992,<br>0.986, 0.983 }
10	RNAPRITFGGPSDSTGSNQNGERSGARSKQ<br>RRPQGLPNNTASWFTALTQHGKEDLKFPRG<br>QGVPINTNSSPDDQIGYYRRATRRIRGGDG<br>KMKDLSPRWYFYYLGTGPEAGLPYGANKDG<br>IIWVATEGALNTPKDHIGTRNPANNAAIVL<br>QLPQGTTLPKGFYAEGSRGGSQASSRSSSR<br>SRNSSRNSTPGSSRGTSPARMAGNGGDAAL<br>ALLLLDRLNQLESKMSGKGQQQQGQTVTKK<br>SAAEASKKPRQKRTATKAYNVTQAFGRRGP<br>EQTQGNFGDQELIRQGTDYKHWPQIAQFAP<br>SASAFFGMSRIGMEVTPSGTWLTYTGAIKL<br>DDKDPNFKDQVILLNKHIDAYKTFPPTEPK<br>KDKKKKADETQALPQRQKKQQTVTLLPAAD<br>LDDFSKQLQQSMSSADS orf9	MHLYKPACADIPSPKLGLPKSSESALKCRW<br>HLAVTKTQPQAACKPVRPSGAAEQKYVEKF<br>LRVHGISLQETTRAETGMAYRNLGKSGLRV<br>SCLGLGTWVTFGGQISDEVAERLMTIAYES<br>GVNLFDTAEVYAAGKAEVILGSIIKKKGWR<br>RSSLVITTKLYWGGKAETERGLSRKHIIEG<br>LKGSLQRLQLEYVDVVFANRPDSNTPMEEI<br>VRAMTHVINQGMAMYWGTSRWSAMEIMEAY<br>SVARQFNMIPPVCEQAEYHLFQREKVEVQL<br>PELYHKIGVGAMTWSPLACGIISGKYGNGV<br>PESSRASLKCYQWLKERIVSEEGRKQQNKL<br>KDLSPIAERLGCTLPQLAVAWCLRNEGVSS<br>VLLGSSTPEQLIENLGAIQVLPKMTSHVVN<br>EIDNILRNKPYSKKDYR KCNAB1<br>JHU13741 { 0.983, 0.983,<br>0.983 }
11	YDHVISTSHKLVLSVNPYVCNAPGCDVTDV<br>TQLYLGGMSYYCKSHKPPISFPLCANGQVF<br>GLYKNTCVGSDNVTDFNAIATCDWTNAGDY<br>ILANTCTERLKLFAAETLKATEETFKLSYG<br>IATVREVLSDRELHLSWEVGKPRPPLNRNY<br>VFTGYRVTKNSKVQIGEYTFEKGDYGDAVV<br>YRGTTTYKLNVGDYFVLTSHTVMPLSAPTL<br>VPQEHYVRITGLYPTLNISDEFSSNVANYQ<br>KVGMQKYSTLQGPPGTGKSHFAIGLALYYP<br>SARIVYTACSHAAVDALCEKALKYLPIDKC<br>SRIIPARARVECFDKFKVNSTLEQYVFCTV<br>NALPETTADIVVFDEISMATNYDLSVVNAR<br>LRAKHYVYIGDPAQLPAPRTLLTKGTLEPE<br>YFNSVCRLMKTIGPDMFLGTCRRCPAEIVD<br>TVSALVYDNKLKAHKDKSAQCFKMFYKGVI	MSTETELQVAVKTSAKKDSRKKGQDRSEAT<br>LIKRFKGEGVRYKAKLIGIDEVSAARGDKL<br>CQDSMMKLKGVVAGARSKGEHKQKIFLTIS<br>FGGIKIFDEKTGALQHHHAVHEISYIAKDI<br>TDHRAFGYVCGKEGNHRFVAIKTAQAAEPV<br>ILDLRDLFQLIYELKQREELEKKAQKDKQC<br>EQAVYQVPTSQKKEGVYDVPKSQPVSNGYS<br>FEDFEERFAAATPNRNLPTDFDEIFEATKA<br>VTQLELFGDMSTPPDITSPPTPATPGDAFI<br>PSSSQTLPASADVFSSVPFGTAAVPSGYVA<br>MGAVLPSFWGQQPLVQQQMVMGAQPPVAQV<br>MPGAQPIAWGQPGLFPATQQPWPTVAGQFP<br>PAAFMPTQTVMPLAAMFQGPLTPLATVPG<br>TSDSTRSSPQTDKPRQKMGKETFKDFQMAQ<br>PPPVPSRKPDQPSLTCTSEAFSSYFNKVGV

	THDVSSAINRPQIGVVREFLTRNPAWRKAV FISPYNSQNAVASKILGLPTQTVDSSQGSE YDYVIFTQTTETAHSCNVNRFNVAITRAKV GILCIMSDRDLY nsp13	AQDTDDCDDFDISQLNLTPVTSTTPSTNSP PTPAPRQSSPSKSSASHASDPTTDDIFEEG FESPSKSEEQEAPDGSQASSNSDPFGEPSG EPSGDNISPQAG DAB1 JHU09621 { 0.987, 0.987, 0.981 }
12	WTNAGDYILANTCTERLKLFAAETLKATEE TFKLSYGIATVREVLSDRELHLSWEVGKPR PPLNRNYVFTGYRVTKNSKVQIGEYTFEKG DYGDAVVYRGTTTYKLNVGDYFVLTSHTVM PLSAPTLVPQEHYVRITGLYPTLNISDEFS SNVANYQKVGMQKYSTLQGPPGTGKSHFAI GLALYYPSARIVYTACSHAAVDALCEKALK YLPIDKCSRIIPARARVECFDKFKVNSTLE QYVFCTVNALPETTADIVVFDEISMATNYD LSVVNARLRAKHYVYIGDPAQLPAPRTLLT KGTLEPEYFNSVCRLMKTIGPDMFLGTCRR CPAEIVDTVSALVYDNKLKAHKDKSAQCFK MFYKGVITHDVSSAINRPQIGVVREFLTRN PAWRKAVFISPYNSQNAVASKILGLPTQTV DSSQGSEYDYVIFTQTTETAHSCNVNRFNV AITRAKVGILCIMSDRDL nsp13	MSTETELQVAVKTSAKKDSRKKGQDRSEAT LIKRFKGEGVRYKAKLIGIDEVSAARGDML CQDSMMKLKGVVAGARSKGEHKQKIFLTIS FGGIKIFDEKTGVPTSQKKEGVYDVPKSQP VSNGYSFEDFEERFAAATPNRNLPTDFDEI FEATKAVTQLELFGDMSTPPDITSPSTPAT PGDAFIPSSSQTLPASADVFSSVPFGTAAV PSGYVAMGAVLPSFWGQQPLVQQMVMGAQ PPVAQVMPGAQPIAWGQPGLFPATQQPWPT VAGQFPPAAFMPTQTVMPLPAAMFQGPLTP LATVPGTSDSTRSSPQTDKPRQKMGKETFK DFQMAQPPPVPSRKPDQPSLTCTSEAFSSY FNKVGVAQDTDDCDDFDISQLNLTPVTSTT PSTNSPPTPAPRQSSPSKSSASHASDPTTD DIFEEGFESPSKSEEQEAPDGSQASSNSDP FGEPSGEPSGDNISPQAG DAB1 JHU10095 {0.988, 0.986, 0.985 }
13	CTERLKLFAAETLKATEETFKLSYGIATVR EVLSDRELHLSWEVGKPRPPLNRNYVFTGY RVTKNSKVQIGEYTFEKGDYGDAVVYRGTT TYKLNVGDYFVLTSHTVMPLSAPTLVPQEH YVRITGLYPTLNISDEFSSNVANYQKVGMQ KYSTLQGPPGTGKSHFAIGLALYYPSARIV YTACSHAAVDALCEKALKYLPIDKCSRIIP ARARVECFDKFKVNSTLEQYVFCTVNALPE TTADIVVFDEISMATNYDLSVVNARLRAKH YVYIGDPAQLPAPRTLLTKGTLEPEYFNSV CRLMKTIGPDMFLGTCRRCPAEIVDTVSAL VYDNKLKAHKDKSAQCFKMFYKGVITHDVS SAINRPQIGVVREFLTRNPAWRKAVFISPY NSQNAVASKI nsp13	MFTEGEEMYLQGSTVWHPDCKQSTKTEEKL RAKVDNEILDYKDLAAIPKVKAIYDIERPD LITYEPFYTSGYDDKQERQSLGESPRTLSP TPSAEGYQDVRDRMIHRSTSQGSINSPVYS RHSYTPTTSRSPQHFHRPDQGINIYRKPPI YKQHAALAAQSKSSEDIIKFSKFPAAQAPD PSETPKIETDHWPGPPSFAVVGPDMKRRSS GREEDDEELLRRRQLQEEQLMKLNSGLGQL ILKEEMEKESRERSSLLASRYDSPINSASH IPSSKTASLPGYGRNGLHRPVSTDFAQYNS YGDVSGGVRDYQTLPDGHMPAMRMDRGVSM PNMLEPKIFPYEMLMVTNRGRNKILREVDR TRLERHLAPEVFREIFGMSIQEFDRLPLWR RNDMKKKAKL ABLIM1 JHU09408 { 0.991, 0.989, 0.987 }
14	WTNAGDYILANTCTERLKLFAAETLKATEE TFKLSYGIATVREVLSDRELHLSWEVGKPR PPLNRNYVFTGYRVTKNSKVQIGEYTFEKG DYGDAVVYRGTTTYKLNVGDYFVLTSHTVM PLSAPTLVPQEHYVRITGLYPTLNISDEFS SNVANYQKVGMQKYSTLQGPPGTGKSHFAI GLALYYPSARIVYTACSHAAVDALCEKALK YLPIDKCSRIIPARARVECFDKFKVNSTLE QYVFCTVNALPETTADIVVFDEISMATNYD LSVVNARLRAKHYVYIGDPAQLPAPRTLLT KGTLEPEYFNSVCRLMKTIGPDMFLGTCRR CPAEIVDTVSALVYDNKLKAHKDKSAQCFK MFYKGVITHDVSSAINRPQIGVVREFLTRN PAWRKAVFISPYNSQNAVASKILGLPTQTV DSSQGSEYDYVIFTQTTETAHSCNVNRFNV AITRAKVGILCIMSDRDL nsp13	MSTETELQVAVKTSAKKDSRKKGQDRSEAT LIKRFKGEGVRYKAKLIGIDEVSAARGDML CQDSMMKLKGVVAGARSKGEHKQKIFLTIS FGGIKIFDEKTGVPTSQKKEGVYDVPKSQP VSNGYSFEDFEERFAAATPNRNLPTDFDEI FEATKAVTQLELFGDMSTPPDITSPSTPAT PGDAFIPSSSQTLPASADVFSSVPFGTAAV PSGYVAMGAVLPSFWGQQPLVQQMVMGAQ PPVAQVMPGAQPIAWGQPGLFPATQQPWPT VAGQFPPAAFMPTQTVMPLPAAMFQGPLTP LATVPGTSDSTRSSPQTDKPRQKMGKETFK DFQMAQPPPVPSRKPDQPSLTCTSEAFSSY FNKVGVAQDTDDCDDFDISQLNLTPVTSTT PSTNSPPTPAPRQSSPSKSSASHASDPTTD DIFEEGFESPSKSEEQEAPDGSQASSNSDP FGEPSGEPSGDNISPQAG DAB1

		JHU10095 { 0.988, 0.986, 0.985 }
15	PGCDVTDVTQLYLGGMSYYCKSHKPPISFPLCANGQVFGLYKNTCVGSDNVTDFNAIATCDWTNAGDYILANTCTERLKLFAAETLKATEETFKLSYGIATVREVLSDRELHLSWEVGKPRPPLNRNYVFTGYRVTKNSKVQIGEYTFEKGDYGDAVVYRGTTTYKLNVGDYFVLTSHTVMPLSAPTLVPQEHYVRITGLYPTLNISDEFSSNVANYQKVGMQKYSTLQGPPGTGKSH nsp13	MEKEKGNDDGIPDQENSLDFSEHFNQLELLETHGHLIPTGTQSLWVGNSDEDEEQDDKNEEWYRLQEKKMEKDPSRLLLWAAEKNRLTTVRRLLSEKATHVNTRDEDEYTPLHRAAYSGHLDIVQELIAQGADVHAVTVDGWTPLHSACKWNNTRVASFLLQHDADINAQTKGLLTPLHLAAGNRDSKDTLELLLMNRYVKPGLKNNLEETAFDIARRTSIYHYLFEIVEGCTNSSPQ ANKRD49 JHU07970 { 0.990, 0.989, 0.983 }
16	APGCDVTDVTQLYLGGMSYYCKSHKPPISFPLCANGQVFGLYKNTCVGSDNVTDFNAIATCDWTNAGDYILANTCTERLKLFAAETLKATEETFKLSYGIATVREVLSDRELHLSWEVGKPRPPLNRNYVFTGYRVTKNSKVQIGEYTFEKGDYGDAVVYRGTTTYKLNVGDYFVLTSHTVMPLSAPTLVPQEHYVRITGLYPTLNISDEFSSNVANYQKVGMQKYSTLQGPPGTGKSHFAIGLALYYPSARIVYTACSHAAVDALCEKALKYLPIDKCSRIIPARARVECFDKFKVNSTLEQYVFCTVNALPETTADIVVFDEISMATNYDLSVVNARLRAKHYVYIGDPAQLPAPRTLLTKGTLEPEYFNSVCRLMKTIGPDMFLGTCRRCPAEIVDTVSALVYDNKLKAHKDKSAQCFKMFYKGVITHDVSSAINRPQIGVVREFLTRNPAWRKAVFISPYNSQNAVASKILGLPTQTVDSSQGSEYDYVIFTQTTETAHSCNVNRFNVAIT nsp13	MSAETPITLNIDPQDLQVQTFTVEKLLEPLIIQVTTLVNCPQNPSSRKKGRSKRASVLLASVEEATWNLLDKGEKIAQEATVLKDELTASLEEVRKESEALKVSAERFTDDPCFLPKREAVVQAARALLAAVTRLLILADMIDVMCLLQHVSAFQRTFESLKNVANKSDLQKTYQKLGKELENLDYLAFKRQQDLKSPNQRDEIAGARASLKENSPLLHSICSACLEHSDVASLKASKDTVCEEIQNALNVISNASQGIQNMTTPPEPQAATLGSALDELENLIVLNPLTVTEEEIRPSLEKRLEAIISGAALLADSSCTRDLHRERIIAECNAIRQALQDLLSEYMNNAGKKERSNTLNIALDNMCKKTRDLRRQLRKAIIDHVSDSFLDTTVPLLVLIEAAKNGREKEIKEYAAIFHEHTSRLVEVANLACSMSTNEDGIKIVKIAANHLETLCPQIINAALALAARPKSQAVKNTMEMYKRTWENHIHVLTEAVDDITSIDDFLAVSGMFLF CTNNA3 JHU16269 { 0.992, 0.989, 0.988 }
17	AVGACVLCNSQTSLRCGACIRRPFLCCKCCYDHVISTSHKLVLSVNPYVCNAPGCDVTDVTQLYLGGMSYYCKSHKPPISFPLCANGQVFGLYKNTCVGSDNVTDFNAIATCDWTNAGDYILANTCTERLKLFAAETLKATEETFKLSYGIATVREVLSDRELHLSWEVGKPRPPLNRNYVFTGYRVTKNSKVQIGEYTFEKGDYGDAVVYRGTTTYKLNVGDYFVLTSHTVMPLSAPTLVPQEHYVRITGLYPTLNISDEFSSNVANYQKVGMQKYSTLQGPPGTGKSHFAIGLALYYPSARIVYTACSHAAVDALCEKALKYLPIDKCSRIIPARARVECFDKFKVNSTLEQYVFCTVNALPETTADIVVFDEISMATNYDLSVVNARLRAKHYVYIGDPAQLPAPRTLLTKGTLEPEYFNSVCRLMKTIGPDMFLGTCRRCPAEIVDTVSALVYDNKLKAHKDKSAQCFKMFYKGVITHDVSSAINRPQIGVVREFLTRNPAWRKAVFISPYNSQNAVASKILGLPTQTVDSSQGSEYDYVIFTQTTETAHSCNVNRFNVAITRAKVGILCIMSDRDLYDKLQFTSLEIPRRNVATLQ nsp13	PALMEPGAFSGARPGLGGYSPPPEEAMPFEFDQPAQRGCSQLLLQVPDLAPGGPGAAGVPGAPPEEPQALRPAKAGSRGGYSPPPEETMPFELDGEGFGDDSPPPGLSRVIAQVDGSSQFAAVAASSAVRLTPAANAPPLWVPGAIGSPSQEAVRPPSNFTGSSPWMEISGPPFEIGSAPAGVDDTPVNMDSPPIALDGPPIKVSGAPDKRERAERPPVEEEAAEMEGAADAAEGGKVPSPGYGSPAAGAASADTAARAAPAAPADPDSGATPEDPDSGTAPADPDSGAFAADPDSGAAPAAPADPDSGAAPDAPADPDSGAAPDAPADPDAGAAPEAPAAPAAAETRAAHVAPAAPDAGAPTAPAASATRAAQVRRAASAAPASGARRKIHLRPPSPEIQAADPPTPRPTRASAWRGKSESSRGRRVYYDEGVASSDDDSSGDESDDGTSGCLRWFQHRRNRRRRKPQRNLLRNFLVQAFGGCFGRSESPQPKASRSLKVKKVPLAEKRRQMRKEALEKRAQKRAEKKRSKLIDKQLQDEKMGYMCTHRLLLLGAGESGKSTIVKQMRILHVNGFNGEGGEEDPQAARSNSDGEKATKVQ GNAS JHU28981 { 0.990, 0.986, 0.985 }
18	EYTFEKGDYGDAVVYRGTTTYKLNVGDYFV	MPYPAPNVPVVGITPSQMVANVFGTAGHPQ

	LTSHTVMPLSAPTLVPQEHYVRITGLYPTL<br>NISDEFSSNVANYQKVGMQKYSTLQGPPGT<br>GKSHFAIGLALYYPSARIVYTACSHAAVDA<br>LCEKALKYLPIDKC nsp13	AAHPHQSPSLVRQQTFPHYEASSATTSPFF<br>KPPAQHLNGSAAFNGVDDGRLASADRHTEV<br>PTGTCPVDPFEAQWAALENKSKQRTNPSPT<br>NPFSSDLQKTFEIE NUMB_frag<br>JHU05240 { 0.989, 0.982,<br>0.971 }
19	AVGACVLCNSQTSLRCGACIRRPFLCCKCC<br>YDHVISTSHKLVLSVNPYVCNAPGCDVTDV<br>TQLYLGGMSYYCKSHKPPISFPLCANGQVF<br>GLYKNTCVGSDNVTDFNAIATCDWTNAGDY<br>ILANTCTERLKLFAAETLKATEETFKLSYG<br>IATVREVLSDRELHLSWEVGKPRPPLNRNY<br>VFTGYRVTKNSKVQIGEYTFEKGDYGDAVV<br>YRGTTTYKLNVGDYFVLTSHTVMPLSAPTL<br>VPQEHYVRITGLYPTLNISDEFSSNVANYQ<br>KVGMQKYSTLQGPPGTGKSHFAIGLALYYP<br>SARIVYTACSHAAVDALCEKALKYLPIDKC<br>SRIIPARARVECFDKFKVNSTLEQYVFCTV<br>NALPETTADIVVFDEISMATNYDLSVVNAR<br>LRAKHYVYIGDPAQLPAPRTLLTKGTLEPE<br>YFNSVCRLMKTIGPDMFLGTCRRCPAEIVD<br>TVSALVYDNKLKAHKDKSAQCFKMFYKGVI<br>THDVSSAINRPQIGVVREFLTRNPAWRKAV<br>FISPYNSQNAVASKILGLPTQTVDSSQGSE<br>YDYVIFTQTTETAHSCNVNRFNVAITRAKV<br>GILCIMSDRDLYDKLQFTSLEIPRRNVATL<br>Q nsp13	ESHGKEYPEHLPLEVLEAGIKSGRYIQGIL<br>NVNKHRAQIEAFVRLQGASSKDSDLVSDIL<br>IHGMKARNRSIHGDVVVVELLPKNEWKGRT<br>VALCENDCDDKASGESPSEPMPTGRVVGIL<br>QKNWRDYVVTFPSKEEVQSQGKNAQKILVT<br>PWDYRIPKIRISTQQAETLQDFRVVVRIDS<br>WESTSVYPNGHFVRVLGRIGDLEGEIATIL<br>VENSISVIPFSEAQMCEMPVNTPESPWKVS<br>PEEEQKRKDLRKSHLVFSIDPKGCEDVDDT<br>LSVRTLNNGNLELGVHIADVTHFVAPNSYI<br>DIEARTRATTYYLADRRYDMLPSVLSADLC<br>SLLGGVDRYAVSIMWELDKASYEIKKVWYG<br>RTIIRSAYKLFYEAAQELLDGNLSVVDDIP<br>EFKDLDEKSRQAKLEELVWAIGKLTDIARH<br>VRAKRDGCGALELEGVEVCVQLDDKKNIHD<br>LIPKQPLEVHETVAECMILANHWVAKKIWE<br>SFPHQALLRQHPPPHQEFFSELRECAKAKG<br>FFIDTRSNKTLADSLDNANDPHDPIVNRLL<br>RSMATQAMSNALYFSTGSCAEEEFHHYGLA<br>LDKYTHFTSPIRRYSDIVVHRLLMAAISKD<br>K DIS3L JHU09250 { 0.991,<br>0.988, 0.988 }
20	LKLFAAETLKATEETFKLSYGIATVREVLS<br>DRELHLSWEVGKPRPPLNRNYVFTGYRVTK<br>NSKVQIGEYTFEKGDYGDAVVYRGTTTYKL<br>NVGDYFVLTSHTVMPLSAPTLVPQEHYVRI<br>TGLYPTLNISDEFSSNVANYQKVGMQKYST<br>LQGPPGTGKSHFAIGLALYYPSARIVYTAC<br>SHAAVDALCEKALKYLPIDKCSRIIPARAR<br>VECFDKFKVNSTLEQYVFCTVNALPETTAD<br>IVVFDEISMATNYDLSVVNARLRAKHYVYI<br>GDPAQLPAPRTLLTKGTLEPEYFNSVCRLM<br>KTIGPDMFLGTCRRCPAEIVDTVSALVYDN<br>KLKAHKDKSAQCFKMFYKGVITHDVSSAIN<br>RPQIGVVREFLTRNPAWRKAVFISPYNSQN<br>AVASKILGLPTQTVDSSQGSEYDYVIFTQT<br>TETAHSCNVNRF nsp13	MPDYLGADQRKTKEDEKDDKPIRALDEGDI<br>ALLKTYGQSTYSRQIKQVEDDIQQLLKKIN<br>ELTGIKESDTGLAPPALWDLAADKQTLQSE<br>QPLQVARCTKIINADSEDPKYIINVKQFAK<br>FVVDLSDQVAPTDIEEGMRVGVDRNKYQIH<br>IPLPPKIDPTVTMMQVEEKPDVTYSDVGGC<br>KEQIEKLREVVETPLLHPERFVNLGIEPPK<br>GVLLFGPPGTGKTLCARAVANRTDACFIRV<br>IGSELVQKYVGEGARMVRELFEMARTKKAC<br>LIFFDEIDAIGGARFDDGAGGDNEVQRTML<br>ELINQLDGFDPRGNIKVLMATNRPDTLDPA<br>LMRPGRLDRKIEFSLPDLEGRTHIFKIHAR<br>SMSVERDIRFELLARLCPNSTGAEIRSVCT<br>EAGMFAIRARRKIATEKDFLEAVNKVIKSY<br>AKFSATPRYMTY PSMC2 JHU00355<br>{ 0.990, 0.989, 0.988 }
21	SSQAWQPGVAMPNLYKMQRMLLEKCDLQNY<br>GDSATLPKGIMMNVAKYTQLCQYLNTLTLA<br>VPYNMRVIHFGAGSDKGVAPGTAVLRQWLP<br>TGTLLVDSDLNDFVSDADSTLIGDCATVHT<br>ANKWDLIISDMYDPKTKNVTKENDSKEGFF<br>TYICGFIQQKLALGGSVAIKITEHSWNADL<br>YKLMGHFAWWTAFVTNVNASSSEAFLIGCN<br>YLGKPREQIDGYVMHANYIFWRNTNPIQLS<br>SYSLFDMSKFPLKLRGTAVMSLKEGQINDM<br>ILSLLSKGRLIIRENNRVVISSDVLVNN	RGSRMNGLPSPTHSAHCSFYRTRTLQALSN<br>EKKAKKVRFYRNGDRYFKGIVYAVSSDRFR<br>SFDALLADLTRSLSDNINLPQGVRYIYTID<br>GSRKIGSMDELEEGESYVCSSDNFFKKVEY<br>TKNVNPNWSVNVKTSANMKAPQSLASSNSA<br>QARENKDFVRPKLVTIIRSGVKPRKAVRVL<br>LNKKTAHSFEQVLTDITEAIKLETGVVKKL<br>YTLDGKQVTCLHDFFGDDDVFIACGPEKFR<br>YAQDDFSLDENECRVMKGNPSATAGPKASP<br>TPQKTSAKSPGPMRRSKSPADSGNDQDA

	nsp16	DCX JHU04060 { 0.987, 0.986, 0.985 }
22	SSQAWQPGVAMPNLYKMQRMLLEKCDLQNY GDSATLPKGIMMNVAKYTQLCQYLNTLTLA VPYNMRVIHFGAGSDKGVAPGTAVLRQWLP TGTLLVDSDLNDFVSDADSTLIGDCATVHT ANKWDLIISDMYDPKTKNVTKENDSKEGFF TYICGFIQQKLALGGSVAIKITEHSWNADL YKLMGHFAWWTAFVTNVNASSSEAFLIGCN YLGKPREQIDGYVMHANYIFWRNTNPIQLS SYSLFDMSKFPLKLRGTAVMSLKEGQINDM ILSLLSKGRLIIRENNRVVISSDVLVNN nsp16	RGSRMNGLPSPTHSAHCSFYRTRTLQALSN EKKAKKVRFYRNGDRYFKGIVYAVSSDRFR SFDALLADLTRSLSDNINLPQGVRYIYTID GSRKIGSMDELEEGESYVCSSDNFFKKVEY TKNVNPNWSVNVKTSANMKAPQSLASSNSA QARENKDFVRPKLVTIIRSGVKPRKAVRVL LNKKTAHSFEQVLTDITEAIKLETGVVKKL YTLDGKQVTCLHDFFGDDDVFIACGPEKFR YAQDDFSLDENECRVMKGNPSATAGPKASP TPQKTSAKSPGPMRRSKSPADSANGTSS DCX JHU03009 { 0.987, 0.986, 0.985 }
23	SSQAWQPGVAMPNLYKMQRMLLEKCDLQNY GDSATLPKGIMMNVAKYTQLCQYLNTLTLA VPYNMRVIHFGAGSDKGVAPGTAVLRQWLP TGTLLVDSDLNDFVSDADSTLIGDCATVHT ANKWDLIISDMYDPKTKNVTKENDSKEGFF TYICGFIQQKLALGGSVAIKITEHSWNADL YKLMGHFAWWTAFVTNVNASSSEAFLIGCN YLGKPREQIDGYVMHANYIFWRNTNPIQLS SYSLFDMSKFPLKLRGTAVMSLKEGQINDM ILSLLSKGRLIIRENNRVVISSDVLVNN nsp16	RGSRMNGLPSPTHSAHCSFYRTRTLQALSN EKKAKKVRFYRNGDRYFKGIVYAVSSDRFR SFDALLADLTRSLSDNINLPQGVRYIYTID GSRKIGSMDELEEGESYVCSSDNFFKKVEY TKNVNPNWSVNVKTSANMKAPQSLASSNSA QARENKDFVRPKLVTIIRSGVKPRKAVRVL LNKKTAHSFEQVLTDITEAIKLETGVVKKL YTLDGKQVTCLHDFFGDDDVFIACGPEKFR YAQDDFSLDENECRVMKGNPSATAGPKASP TPQKTSAKSPGPMRRSKSPADSGNDQDA DCX JHU04060 { 0.987, 0.986, 0.985 }
24	SSQAWQPGVAMPNLYKMQRMLLEKCDLQNY GDSATLPKGIMMNVAKYTQLCQYLNTLTLA VPYNMRVIHFGAGSDKGVAPGTAVLRQWLP TGTLLVDSDLNDFVSDADSTLIGDCATVHT ANKWDLIISDMYDPKTKNVTKENDSKEGFF TYICGFIQQKLALGGSVAIKITEHSWNADL YKLMGHFAWWTAFVTNVNASSSEAFLIGCN YLGKPREQIDGYVMHANYIFWRNTNPIQLS SYSLFDMSKFPLKLRGTAVMSLKEGQINDM ILSLLSKGRLIIRENNRVVISSDVLVNN nsp16	SQSQAEIRKQILGSSSSGKFCLYTEEFAS KDMTPLKPAEMQEANLTSMVLFMKRIDIAG LGHCDFMNRPAPESLMQALEDLDYLAALDN DGNLSEFGIIMSEFPLDPQLSKSILASCEF DCVDEVLTIAAMVTAPNCFSHVPHGAEEAA LTCWKTFLHPEGDHFTLISIYKAYQDTTLN SSSEYCVEKWCRDYFLNCSALRMADVIRAE LLEIIKRIELPYAEPAFGSKENTLNIKKAL LSGYFMQIARDVDGSGNYLMLTHKQVAQLH PLSGYSITKKMPEWVLFHKFSISENNYI DHX32 JHU10488 { 0.989, 0.985, 0.984 }
25	SSQAWQPGVAMPNLYKMQRMLLEKCDLQNY GDSATLPKGIMMNVAKYTQLCQYLNTLTLA VPYNMRVIHFGAGSDKGVAPGTAVLRQWLP TGTLLVDSDLNDFVSDADSTLIGDCATVHT ANKWDLIISDMYDPKTKNVTKENDSKEGFF TYICGFIQQKLALGGSVAIKITEHSWNADL YKLMGHFAWWTAFVTNVNASSSEAFLIGCN YLGKPREQIDGYVMHANYIFWRNTNPIQLS SYSLFDMSKFPLKLRGTAVMSLKEGQINDM ILSLLSKGRLIIRENNRVVISSDVLVNN nsp16	MASRQPEVPALEASAPLGKMSLPIGIYRRA VSYDDTLEDPAPMTPPPSDMGSVPWKPVIP ERKYQHLAKVEEGEASLPSPAMTLSSAIDS VDKVPVVKAKATHVIMNSLITKQTQESIQH FERQAGLRDAGYTPHKGLTTEETKYLRVAE ALHKLKLQSGEVTKEERQPASAQSTPSTTP HSSPKQRPRGWFTSGSSTALPGPNPSTMDS GSGDKDRNLSDKWSLFGPRSLQKYDSGSFA TQAYRGAQKPSPLELIRAQANRMAEDPAAL KPPKMDIPVMEGKKQPPRAHNLKPRDLN KIAA1191 JHU07922 { 0.983, 0.982, 0.978 }
26	SSQAWQPGVAMPNLYKMQRMLLEKCDLQNY GDSATLPKGIMMNVAKYTQLCQYLNTLTLA VPYNMRVIHFGAGSDKGVAPGTAVLRQWLP TGTLLVDSDLNDFVSDADSTLIGDCATVHT	SADMSENSFYLKLDSVTAADTAVYYCAAGH LVMGFGAHWGQGKLVSVSPASTKGPSVFPL APCSRSTSESTAALGCLVKDYFPEPVTVSW NSGALTSGVHTFPAVLQSSGLYSLSSVVTV

	ANKWDLIISDMYDPKTKNVTKENDSKEGFF<br>TYICGFIQQKLALGGSVAIKITEHSWNADL<br>YKLMGHFAWWTAFVTNVNASSSEAFLIGCN<br>YLGKPREQIDGYVMHANYIFWRNTNPIQLS<br>SYSLFDMSKFPLKLRGTAVMSLKEGQINDM<br>ILSLLSKGRLIIRENNRVVISSDVLVNN<br>nsp16	PSSSLGTKTYTCNVDHKPSNTKVDKRVESK<br>YGPPCPSCPAPEFLGGPSVFLFPPKPKDTL<br>MISRTPEVTCVVVDVSQEDPEVQFNWYVDG<br>VEVHNAKTKPREEQFNSTYRVVSVLTVLHQ<br>DWLNGKEYKCKVSNKGLPSSIEKTISKAKG<br>QPREPQVYTLPPSQEEMTKNQVSLTCLV<br>IGHG4 JHU03305 { 0.986, 0.985,<br>0.984 }
27	SSQAWQPGVAMPNLYKMQRMLLEKCDLQNY<br>GDSATLPKGIMMNVAKYTQLCQYLNTLTLA<br>VPYNMRVIHFGAGSDKGVAPGTAVLRQWLP<br>TGTLLVDSDLNDFVSDADSTLIGDCATVHT<br>ANKWDLIISDMYDPKTKNVTKENDSKEGFF<br>TYICGFIQQKLALGGSVAIKITEHSWNADL<br>YKLMGHFAWWTAFVTNVNASSSEAFLIGCN<br>YLGKPREQIDGYVMHANYIFWRNTNPIQLS<br>SYSLFDMSKFPLKLRGTAVMSLKEGQINDM<br>ILSLLSKGRLIIRENNRVVISSDVLVNN<br>nsp16	PSDGQAREKLALYVYEYLLHVGAQKSAQTF<br>LSEIRWEKNITLGEPPGFLHSWWCVFWDLY<br>CAAPERRDTCEHSSEAKAFHDYSAAAAPSP<br>VLGNIPPNDGMPGGPIPPGFFQGPPGSQPS<br>PHAQPPPHNPSSMMGPHSQPFMSPRYAGGP<br>RPPIRMGNQPPGGVPGTQPLLPNSMDPTRQ<br>QGHPNMGGSMQRMNPPRGMGPMGPGPQNYG<br>SGMRPPPNSLGPAMPGINMGPGAGRPWPNP<br>NSANSIPYSSSSPGTYVGPPGGGGPPGTPI<br>MPSPADSTNSSDNIYTMINPVPPGGSRS<br>SSBP3 JHU09872 { 0.983, 0.982,<br>0.975 }
28	SSQAWQPGVAMPNLYKMQRMLLEKCDLQNY<br>GDSATLPKGIMMNVAKYTQLCQYLNTLTLA<br>VPYNMRVIHFGAGSDKGVAPGTAVLRQWLP<br>TGTLLVDSDLNDFVSDADSTLIGDCATVHT<br>ANKWDLIISDMYDPKTKNVTKENDSKEGFF<br>TYICGFIQQKLALGGSVAIKITEHSWNADL<br>YKLMGHFAWWTAFVTNVNASSSEAFLIGCN<br>YLGKPREQIDGYVMHANYIFWRNTNPIQLS<br>SYSLFDMSKFPLKLRGTAVMSLKEGQINDM<br>ILSLLSKGRLIIRENNRVVISSDVLVNN<br>nsp16	VPPGRAAGSGWAWRPVARDALLARAFHSCT<br>ELRGRFYLVGGLLAGGAREPSSDTVVFDPA<br>RGQAVRLGARGSPPRSHHDAAPVDGRWLCV<br>VGGWDGSRRLATVTALDTERGVWEAWTGTP<br>GDCPPAGLSSHTCTRISDRELQVAGREGGI<br>HTQRRYGSIYTLRLDPSARTYCYKQEGCHT<br>ASRSGHCAALLQTPGPHPGHQLLLFGGCNL<br>AEPEVAGHWSHGKIKEEPPVAPHLMEQLAR<br>LVSSGQGSQKGPHGLRHHSCSVVGPFAVLF<br>GGETLTRTRDTICNDLYIYDTRTSPPLW<br>KLHDC9 JHU15417 { 0.983,<br>0.983, 0.981 }
29	SSQAWQPGVAMPNLYKMQRMLLEKCDLQNY<br>GDSATLPKGIMMNVAKYTQLCQYLNTLTLA<br>VPYNMRVIHFGAGSDKGVAPGTAVLRQWLP<br>TGTLLVDSDLNDFVSDADSTLIGDCATVHT<br>ANKWDLIISDMYDPKTKNVTKENDSKEGFF<br>TYICGFIQQKLALGGSVAIKITEHSWNADL<br>YKLMGHFAWWTAFVTNVNASSSEAFLIGCN<br>YLGKPREQIDGYVMHANYIFWRNTNPIQLS<br>SYSLFDMSKFPLKLRGTAVMSLKEGQINDM<br>ILSLLSKGRLIIRENNRVVISSDVLVNN<br>nsp16	PSPKLGLPKSSESALKCRWHLAVTKTQPQA<br>ACKPVRPSGAAEQKYVEKFLRVHGISLQET<br>TRAETGMAYRNLGKSGLRVSCLGLGTWVTF<br>GGQISDEVAERLMTIAYESGVNLFDTAEVY<br>AAGKAEVILGSIIKKKGWRRSSLVITTKLY<br>WGGKAETERGLSRKHIIEGLKGSLQRLQLE<br>YVDVVFANRPDSNTPMEEIVRAMTHVINQG<br>MAMYWGTSRWSAMEIMEAYSVARQFNMIPP<br>VCEQAEYHLFQREKVEVQLPELYHKIGVGA<br>MTWSPLACGIISGKYGNGVPESSRASLK<br>KCNAB1 JHU13741 { 0.991,<br>0.984, 0.984 }
30	SQAWQPGVAMPNLYKMQRMLLEKCDLQNYG<br>DSATLPKGIMMNVAKYTQLCQYLNTLTLAV<br>PYNMRVIHFGAGSDKGVAPGTAVLRQWLPT<br>GTLLVDSDLNDFVSDADSTLIGDCATVHTA<br>NKWDLIISDMYDPKTKNVTKENDSKEGFFT<br>YICGFIQQKLALGGSVAIKITEHSWNADLY<br>KLMGHFAWWTAFVTNVNASSSEAFLIGCNY<br>LGKPREQIDGYVMHANYIFWRNTNPIQLSS<br>YSLFDMSKFPLKLRGTAVMSLKEGQINDMI<br>LSLL nsp16	MPKVFLVKRRSLGVSVRSWDELPDEKRADT<br>YIPVGLGRLLHDPPEDCRSDGGSSSGSGSS<br>SAGEPGGAESSSSPHAPESETPEPGDAEGP<br>DGHLATKQRPVARSKIKFTTGTCSDSVVHS<br>CDLCGKGFRLQRMLNRHLKCHNQVKRHLCT<br>FCGKGFNDTFDLKRHVRTHTGIRPYKCNVC<br>NKAFTQRCSLESHLKKIHGVQQQYAYKQRR<br>DKLYVCEDCGYTGPTQEDLYLHVNSAHPGS<br>SFLKKTSKKLAALLQGKLTSAHQENTSLSE<br>EEER OVOL2 JHU04191 { 0.987,

		0.984, 0.983 }
31	SNCVADYSVLYNSASFSTFKCYGVSPTKLN<br>DLCFTNVYADSFVIRGDEVRQIAPGQTGKI<br>ADYNYKLPDDFTGCVIAWNSNNLDSKVGGN<br>YNYLYRLFRKSNLKPFERDISTEIYQAGST<br>PCNGVEGFNCYFPLQSYGFQPTNGVGYQPY<br>RVVVLSFELLHAPATVCGPKKSTNLVKNKC<br>VNFNFNGLTGTGVLTESNKKFLPFQQFGRD<br>IADTTDAVRDPQTLEILDITPCSFGGVSVI<br>TPGTNTSNQVAVLYQDVNCTEVPVAIHADQ<br>LTPTWRVYSTGSNVFQTRAGCLIGAEHVNN<br>SYECDIPIGAGICASYQTQTNSPRRARSVA<br>SQSIIAYTMSLGAENSVAYSNNSIAIPTNF<br>TISVTTEILPVSMTKTSVDCTMYICGDSTE<br>CSNLLLQYGSFCTQLNRALTGIAVEQDKNT<br>QEVFAQVKQIYKTPPIKDFGGFNFSQI<br>Spike	MKGFIDDANYSVGLLDEGTNLGNVIDNYVY<br>EHTLTGKNAFFVGDLGKIVKKHSQWQNVVA<br>QIKPFYTVKCNSAPAVLEILAALGTGFACS<br>SKNEMALVQELGVPPENIIYISPCKQVSQI<br>KYAAKVGVNILTCDNEIELKKIARNHPNAK<br>VLLHIATEDNIGGEEGNMKFGTTLKNCRHL<br>LECAKELDVQIIGVKFHVSSACKESQVYVH<br>ALSDARCVFDMAGEIGFTMNMLDIGGGFTG<br>TEFQLEEVNHVISPLLDIYFPEGSGVKIIS<br>EPGSYYVSSAFTLAVNIIAKKVVENDKFPS<br>GVEKTGSDEPAFMYYMNDGVYGSFASKLSE<br>DLNTIPEVHKKYKEDEPLFTSSLWGPSCDE<br>LDQIVESCLLPELNVGDWLIFDNMGADSFH<br>EPSAFNDFQRPAIYYMMSFSDWYEMQDAGI<br>TSDSMMKNFFFVPSCIQLSQEDSFSAE<br>AZIN1 JHU00873 { 0.984, 0.983,<br>0.983 }
32	TGVLTESNKKFLPFQQFGRDIADTTDAVRD<br>PQTLEILDITPCSFGGVSVITPGTNTSNQV<br>AVLYQDVNCTEVPVAIHADQLTPTWRVYST<br>GSNVFQTRAGCLIGAEHVNNSYECDIPIGA<br>GICASYQTQTNSPRRARSVASQSIIAYTMS<br>LGAENSVAYSNNSIAIPTNFTISVTTEILP<br>VSMTKTSVDCTMYICGDSTECSNLLLQYGS<br>FCTQLNRALTGIAVEQDKNTQEVFAQVKQI<br>YKTPPIKDFGGFNFSQILPDPSKPSKRSFI<br>EDLLFNKVTLADAGFIKQYGDCLGDIAARD<br>LICAQKFNGLTVLPPLLTDEMIAQYTSALL<br>AGTI Spike	MASASSSRAGVALPFEKSQLTLKVVSAKPK<br>VHNRQPRINSYVEVAVDGLPSETKKTGKRI<br>GSSELLWNEIIILNVTAQSHLDLKVWSCHT<br>LRNELLGTASVNLSNVLKNNGGKMENMQLT<br>LNLQTENKGSVVSGGELTIFLDGPTVDLGN<br>VPNGSALTDGSQLPSRDSSGTAVAPENRHQ<br>PPSTNCFGGRSRTHRHSGASARTTPATGEQ<br>SPGARSRHRQPVKNSGHSGLANGTVNDEPT<br>TATDPEEPSVVGVTSPPAAPLSVTPNPNTT<br>SLPAPATPAEGEEPSTSGTQQLPAAAQAPD<br>ALPAGWEQRELPNGRVYYVDHNTKTTTWER<br>PLPP WWP2 JHU04987 { 0.986,<br>0.985, 0.985 }
33	YQDVNCTEVPVAIHADQLTPTWRVYSTGSN<br>VFQTRAGCLIGAEHVNNSYECDIPIGAGIC<br>ASYQTQTNSPRRARSVASQSIIAYTMSLGA<br>ENSVAYSNNSIAIPTNFTISVTTEILPVSM<br>TKTSVDCTMYICGDSTECSNLLLQYGSFCT<br>QLNRALTGIAVEQDKNTQEVFAQVKQIYKT<br>PPIKDFGGFNFSQ Spike	MISRTPEVTCVVVDVSHEDPEVKFNWYVDG<br>VEVHNAKTKPWEEQYNSTYHVVSVLTVVHQ<br>NWLNGKEYKCKVSNKGLQAPIEKTISKTKG<br>QPREPQVYTLPPSQKMTKNQVTLTCLVKGF<br>YPSDIAVEWESNGQPENNYKTTPPMLDSNG<br>SFFLYSKLTVDKSRWQQGNVFSCSVMHEGL<br>QNHYTQKSLSLSP BC031259.1_frag<br>JHU15432 { 0.984, 0.983,<br>0.979 }
34	DLCFTNVYADSFVIRGDEVRQIAPGQTGKI<br>ADYNYKLPDDFTGCVIAWNSNNLDSKVGGN<br>YNYLYRLFRKSNLKPFERDISTEIYQAGST<br>PC Spike	MAQSGGEARPGPKTAVQIRVAIQEAEDVDE<br>LEDEEEGAETRGAGDPARYLSPGWGSASEE<br>EPSRGHRNRSSVNSRTMLASFIVSSAPSTA<br>PS LARP6 JHU10982 { 0.988,<br>0.979, 0.979 }
35	QGFSALEPLVDLPIGINITRFQTLLALHRS<br>YLTPGDSSSGWTAGAAAYYVGYLQPRTFLL<br>KYNENGTITDAVDCALDPLSETKCTLKSFT<br>VEKGIYQTSNFRVQPTESIVRFPNITNLCP<br>FGEVFNATRFASVYAWNRKRISNCVADYSV<br>LYNSASFSTFKCYGVSPTKLNDLCFTNVYA<br>DSFVIRGDEVRQIAPGQTGKIADYNYKLPD<br>DFTGCVIAWN Spike	MCPHLRPVPGTPCLPRWAALSPGDSVPSMP<br>WLAETLNSSLGTVGLSPPLLPCLPLSVPGR<br>LGAGQPEAETKAPHRRAQEVPALGTASSVA<br>PARHQFPEGRSRPPPAAAVGLHSPAQPKAP<br>RSWDSATPSEMLCPFSPVPLDAPSPQCSGD<br>PWGTEQEGLDPVAQPKATRQPQPAQPPSSA<br>WGLATGSSQWTPAPRGGRGTAPTAQQTCEL<br>PECNLCDVFQ CPTP_frag JHU02353<br>{ 0.985, 0.982, 0.981 }

36	SSSGWTAGAAAYYVGYLQPRTFLLKYNENG TITDAVDCALDPLSETKCTLKSFTVEKGIY QTSNFRVQPTESIVRFPNITNLCPFGEVFN ATRFASVYAWNRKRISNCVADYSVLYNSAS FSTFKCYGVSPTKLNDLCFTNVYADSFVIR GDEVRQIAPGQTGKIADYNYKLPDDFTGCV IAWNSNNLDSKVGGNYNYLYRLFRKSNLKP FERDISTEIYQAGSTPCNGVEGFNCYFPLQ SYGFQPTNGVGYQPYRVVVLSFELLHAPAT VCGPKKSTNLVKNKCVNFNFNGLTGTGVLT ESNKKFLPFQQFGRDIADTTDAVRDPQTLE ILDI Spike	MSTEGGFGGTSSSDAQQSLQSFWPRVMEEI RNLTVKDFRVQELPLARIKKIMKLDEDVKM ISAEAPVLFAKAAQIFITELTLRAWIHTED NKRRTLQRNDIAMAITKFDQFDFLIDIVPR DELKPPKRQEEVRQSVTPAEPVQYYFTLAQ QPTAVQVQGQQQGQQTTSSTTTIQPGQIII AQPQQGQTTPVTMQVGEGQQVQIVQAQPQG QAQQAQSGTGQTMQVMQQIITNTGEIQQIP VQLNAGQLQYIRLAQPVSGTQVVQGQIQTL ATNAQQITQTEVQQGQQQFSQFTDGQQLYQ IQQVTMPAGQDLAQPMFIQSANQPSDGQAP QVTG NFYC JHU04956 { 0.987, 0.985, 0.981 }
37	SPRRARSVASQSIIAYTMSLGAENSVAYSN NSIAIPTNFTISVTTEILPVSMTKTSVDCT MYICGDSTECSNLLLQYGSFCTQLNRALTG IAVEQDKNTQEVFAQVKQIYKTPPIKDFGG FNFSQILPDPSKPSKRSFIEDLLFNKVTLA DAGFIKQYGDCLGDIAARDLICAQKFNGLT VLPPLLTDEMIAQYTSALLAGTITSGWTFG AGAALQIPFA Spike	MAGTGLLALRTLPGPSWVRGSGPSVLSRLQ DAAVVRPGFLSTAEEETLSRELEPELRRRR YEYDHWDAAIHGFRETEKSRWSEASRAILQ RVQAAAFGPGQTLLSSVHVLDLEARGYIKP HVDSIKFCGATIAGLSLLSPSVMRLVHTQE PGEWLELLLEPGSLYILRGSARYDFSHEIL RDEESFFGERRIPRGRRISVICRSLPEGMG PGESGQPPPA ALKBH7 JHU04132 { 0.987, 0.985, 0.977 }
38	GFSALEPLVDLPIGINITRFQTLLALHRSY LTPGDSSSGWTAGAAAYYVGYLQPRTFLLK YNENGTITDAVDCALDPLSETKCTLKSFTV EKGIYQTSNFRVQPTESIVRFPNITNLCPF GEVFNATRFASVYAWNRKRISNCVADYSVL YNSASFSTFKCYGVSPTKLNDLCFTNVYAD SFVIRGDEVRQIAPGQTGKIADYNYKLPDD FTGCVIAWNSNNLDSKVGGNYNYLYRLFRK SNLKPFERDISTEIYQAGSTPCNGVEGFNC YFPLQSYGFQPTNGVGYQPYRVVVLSFELL HAPATVCGPKKSTNLVKNKCVNFNFNGLTG TGVLTESNKKFLPFQQFGRDIADTTDAVRD PQTLEILDITPCSFGGVSVITPGTNTSNQV AVLYQDVNCTEVPVAIHADQLTPTWRVYST GSNVFQTRAGCLIGAEHVNNSYECDIPIGA GICASYQTQTNSPRRARSVASQSIIAYTMS LGAENSVAYSNNSIAIPTNFTISVTTEILP VSMTKTSVDCTMYICGDSTECSNLLLQYGS FCTQLNRALTGIAVEQDKNTQEVFAQVKQI YKTPPIKDFGGFNFSQILPDPSKPSKRSFI EDLLFNKVTLADAGFIKQYGDCLGDIAARD LICAQKFNGLTVLPPLLTDEMIAQYTSALL AGTITSGWTFGAGAAL Spike	MPGFLVRILLLLLVLLLLGPTRGLRNATQR MFEIDYSRDSFLKDGQPFRYISGSIHYSRV PRFYWKDRLLKMKMAGLNAIQTYVPWNFHE PWPGQYQFSEDHDVEYFLRLAHELGLLVIL RPGPYICAEWEMGGLPAWLLEKESILLRSS DPDYLAAVDKWLGVLLPKMKPLLYQNGGPV ITVQVENEYGSYFACDFDYLRFLQKRFRHH LGDDVVLFTTDGAHKTFLKCGALQGLYTTV DFGTGSNITDAFLSQRKCEPKGPLINSEFY TGWLDHWGQPHSTIKTEAVASSLYDILARG ASVNLYMFIGGTNFAYWNGANSPYAAQPTS YDYDAPLSEAGDLTEKYFALRNIIQKFEKV PEGPIPPSTPKFAYGKVTLEKLKTVGAALD ILCPSGPIKSLYPLTFIQVKQHYGFVLYRT TLPQDCSNPAPLSSPLNGVHDRAYVAVDGI PQGVLERNNVITLNITGKAGATLDLLVENM GRVNYGAYINDFKGLVSNLTLSSNILTDWT IFPLDTEDAVRSHLGGWGHRDSGHHDEAWA HNSSNYTLPAFYMGNFSIPSGIPDLPQDTF IQFPGWTKGQVWINGFNLGRYWPARGPQLT LFVPQHILMTSAPNTITVLELEWAPCSSDD PELCAVTFVDRPVIGSSVTYDHPSKPVEKR LMPPPPQKNKDSWLDH GLB1 JHU21299 { 0.988, 0.987, 0.984 }
39	QLSSNFGAISSVLNDILSRLDKVEAEVQID RLITGRLQSLQTYVTQQLIRAAEIRASANL AATKMSECVLGQSKRVDFCGKGYHLMSFPQ SAPHGVVFLHVTYVPAQEKNFTTAPAI Spike	MPGAEDDVVTPGTSEDRYKSGLTTLVATSV NSVTGIRIEDLPTSESTVHAQEQSPSATAS NVATSHSTEKVDGDTQTTVEKDGLSTVTLV GIIVGVLLAIGFIGAIIVVVMRKMSGR PDPN JHU14405 { 0.985, 0.985, 0.982 }
40	LLLQYGSFCTQLNRALTGIAVEQDKNTQEV FAQVKQIYKTPPIKDFGGFNFSQILPDPSK	MAAQYGSMSFNPSTPGASYGPGRQEPRNSQ LRIVLVGKTGAGKSATGNSILGRKVFHSGT

	PSKRSFIEDLLFNKVTLADAGFIKQYGDCL GDIAARDLICAQKFNGLTVLPPLLTDEMIA QYTSALLAGTITSGWTFGAGAALQIPFAMQ MAYRFNGIGVTQNVLYENQKLIANQFNSAI GKIQDSLSSTASALGKLQDVVNQNAQALNT LVKQLSSNFGAISSVLNDILSRLDKVEAEV QIDRLITGRLQSLQTYVTQQLIRAAEIRAS ANLAATKMSECVLGQSKRVDFCGKGYHLMS FPQSAPHGVVFLHVTYVPAQEKNFTTAP Spike	AAKSITKKCEKRSSSWKETELVVVDTPGIF DTEVPNAETSKEIIRCILLTSPGPHALLLV VPLGRYTEEEHKATEKILKMFGERARSFMI LIFTRKDDLGDTNLHDYLREAPEDIQDLMD IFGDRYCALNNKATGAEQEAQRAQLLGLIQ RVVRENKEGCYTNRMYQRAEEEIQKQTQAM QELHRVELEREKARIREEYEEKIRKLEDKV EQEKRKKQMEKKLAEQEAHYAVRQQRARTE VESKDGILELIMTALQIASFILLRLFAE GIMAP4 JHU04931 { 0.986, 0.984, 0.984 }
41	MFHLVDFQVTIAEILLIIMRTFKVSIWNLD YIINLIIKNLSKSLTENKYSQLDEEQPMEI D orf6	DQLWEDSVLTVKFPKLMVPRFSFAAPSSED DVFIPTVREVQCPEANIDTALCKESPGLWG A AHNAK2 JHU07968 { 0.985, 0.975, < 0.950 }
42	MFHLVDFQVTIAEILLIIMRTFKVSIWNLD YIINLIIKNLSKSLTENKYSQLDEEQPMEI D orf6	VFSDYYDLGYNMRSNLFRGAAEETKSLMKA SYTPEVIEKSVRDLEHWHGRKTDDLGRWHQ K TEX33 JHU20055 { 0.976, 0.971, 0.960 }
43	MFHLVDFQVTIAEILLIIMRTFKVSIWNLD YIINLIIKNLSKSLTENKYSQLDEEQPMEI D orf6	DVALKNFAKYFLHQSHEEREHAEKLMKLQN QRGGRIFLQDIKEPDCDDWESGQNAMECAL H FTH1 JHU15414 { 0.971, 0.958, 0.953 }
44	MFHLVDFQVTIAEILLIIMRTFKVSIWNLD YIINLIIKNLSKSLTENKYSQLDEEQPMEI D orf6	AEKSSKQKVDLQSLPTRAYLDQTVVPILLQ GLAVLAKERPPNPIEFLASYLLKNKAQFED R DPY30 JHU03411 { 0.983, 0.978, 0.960 }
45	MFHLVDFQVTIAEILLIIMRTFKVSIWNLD YIINLIIKNLSKSLTENKYSQLDEEQPMEI D orf6	MQASEDLLKEHYVDLKDRPFFAGLVKYMHS GPVVAMVWEGLNVVKTGRVMLGETNPADSK P NME1 JHU00057 { 0.975, 0.968, 0.960 }
46	MFHLVDFQVTIAEILLIIMRTFKVSIWNLD YIINLIIKNLSKSLTENKYSQLDEEQPMEI D orf6	VKHSDVLFLAVKPHIIPFILDEIGADVQAR HIVVSCAAGVTISSVEKKLMAFQPAPKVIR C PYCR2 JHU04471 { 0.987, 0.968, 0.960 }
47	MFHLVDFQVTIAEILLIIMRTFKVSIWNLD YIINLIIKNLSKSLTENKYSQLDEEQPMEI D orf6	GSGCFSSGGGGFSGQAVQCQSYGGVSSGGS SGGGSGCFSSGGGGGSVCGYSGGGSGGGSG C LOR JHU15534 { 0.965, 0.952, < 0.950 }
48	MFHLVDFQVTIAEILLIIMRTFKVSIWNLD YIINLIIKNLSKSLTENKYSQLDEEQPMEI D orf6	EFQKILWKEREMRTALEKEIERLESALSLW KWKYEELKESKPKNVKEFDILLGQHNDEMQ E CCDC102B_frag JHU15882 { 0.990, 0.981, 0.959 }
49	MFHLVDFQVTIAEILLIIMRTFKVSIWNLD YIINLIIKNLSKSLTENKYSQLDEEQPMEI D orf6	AEEEELRAELTKVEEEIVTLRQVLAAKERH CGELKRRLGLSTLGELKQNLSRSWHDVQVS S TPD52L2 JHU04498 { 0.976, 0.971, 0.952 }
50	MFHLVDFQVTIAEILLIIMRTFKVSIWNLD YIINLIIKNLSKSLTENKYSQLDEEQPMEI D orf6	VLEPEEDFEQFLLPVINEMREDIASLIREH GRAYLRTRSKLWEMDNMLIQIKTQVEASEE S MRFAP1L1 JHU02929 { 0.982, 0.977, 0.954 }
51	MKFLVFLGIITTVAAFHQECSLQSCTQHQP	QFVEGVRMEEIVEGCTGALHILARDVHNRI

	YVVDDPCPIHFYSKWYIRVGARKSAPLIEL<br>CVDEAGSKSPIQYIDIGNYTVSCLPFTINC<br>QEPKLGSLVVRCSFYEDFLEYHDVRVVLDF<br>I orf8	VIRGLNTIPLFVQLLYSPIENIQRVAAGVL<br>CELAQDKEAAEAIEAEGATAPLTELLHSRN<br>EGVATYAAAVLFRMSEDKPQDYKKRLSVEL<br>T CTNNB1 JHU07025 { 0.984, 0.983, 0.975 }
52	MKFLVFLGIITTVAAFHQECSLQSCTQHQP<br>YVVDDPCPIHFYSKWYIRVGARKSAPLIEL<br>CVDEAGSKSPIQYIDIGNYTVSCLPFTINC<br>QEPKLGSLVVRCSFYEDFLEYHDVRVVLDF<br>I orf8	EKMATVWDEAEQDGIGEEVLKMSTEEIIQR<br>TRLLDSEIKIMKSEVLRVTHELQAMKDKIK<br>ENSEKIKVNKTLPYLVSNVIELLDVDPNDQ<br>EEDGANIDLDSQRKGKCAVIKTSTRQTYFL<br>P PSMC3 JHU13762 { 0.981, 0.978, 0.975 }
53	MKFLVFLGIITTVAAFHQECSLQSCTQHQP<br>YVVDDPCPIHFYSKWYIRVGARKSAPLIEL<br>CVDEAGSKSPIQYIDIGNYTVSCLPFTINC<br>QEPKLGSLVVRCSFYEDFLEYHDVRVVLDF<br>I orf8	LPLESDAVECLNYQHYKGSDFDCELRLLIH<br>QSLAGGIIGVKGAKIKELRENTQTTIKLFQ<br>ECCPHSTDRVVLIGGKPDRVVECIKIILDL<br>ISESPIKGRAQPYDPNFYDETYDYGGFTMM<br>F HNRNPK JHU02733 { 0.982, 0.979, 0.975 }
54	MKFLVFLGIITTVAAFHQECSLQSCTQHQP<br>YVVDDPCPIHFYSKWYIRVGARKSAPLIEL<br>CVDEAGSKSPIQYIDIGNYTVSCLPFTINC<br>QEPKLGSLVVRCSFYEDFLEYHDVRVVLDF<br>I orf8	GLHLASLSHAILEALAAPDRAPFALLALRC<br>ACPEDARASPLPNEAWVALRRRHPGLAVEL<br>ELEPALPAESVTRVLQPAVPVAALRLNLSG<br>DTVGPVRFAAHHYAATLCALEVRAAASAEL<br>N FBXL8 JHU19904 { 0.987, 0.984, 0.983 }
55	MKFLVFLGIITTVAAFHQECSLQSCTQHQP<br>YVVDDPCPIHFYSKWYIRVGARKSAPLIEL<br>CVDEAGSKSPIQYIDIGNYTVSCLPFTINC<br>QEPKLGSLVVRCSFYEDFLEYHDVRVVLDF<br>I orf8	ATQLGATLLRLRGATLAAPGAAEGARLLQA<br>AFAAARCRPPSVLLISELEALLPARDDGAA<br>AGGALQVPLLACLDGGCGAGADGVLVVGTT<br>SRPAALDEATRRRFSLRFYVALPDSPARGQ<br>I FIGNL2 JHU17712 { 0.989, 0.982, 0.969 }
56	MKFLVFLGIITTVAAFHQECSLQSCTQHQP<br>YVVDDPCPIHFYSKWYIRVGARKSAPLIEL<br>CVDEAGSKSPIQYIDIGNYTVSCLPFTINC<br>QEPKLGSLVVRCSFYEDFLEYHDVRVVLDF<br>I orf8	LIRHQRIHTGEKPFECKECGKGFSQNTSLT<br>QHQRIHTGEKPYTCKECGKSFTRNPALLRH<br>QRMHTGEKPYECKDCGKGFMWNSDLSQHQR<br>VHTGDKPHECTDCGKSFFCKAHLIRHQRIH<br>T ZNF662 JHU29296 { 0.979, 0.974, 0.973 }
57	MKFLVFLGIITTVAAFHQECSLQSCTQHQP<br>YVVDDPCPIHFYSKWYIRVGARKSAPLIEL<br>CVDEAGSKSPIQYIDIGNYTVSCLPFTINC<br>QEPKLGSLVVRCSFYEDFLEYHDVRVVLDF<br>I orf8	LTQHQRVHTGERPYECDACGKAFSQSTHLT<br>QHQRIHTGEKPYKCDACGRAFSDCSALIRH<br>LRIHSGEKPYQCKVCPKAFAQSSSLIEHQR<br>IHTGEKPYKCSDCGKAFSRSSALMVHLRIH<br>I ZSCAN22 JHU19974 { 0.980, 0.975, 0.972 }
58	MKFLVFLGIITTVAAFHQECSLQSCTQHQP<br>YVVDDPCPIHFYSKWYIRVGARKSAPLIEL<br>CVDEAGSKSPIQYIDIGNYTVSCLPFTINC<br>QEPKLGSLVVRCSFYEDFLEYHDVRVVLDF<br>I orf8	ALKSLSLNSPVQPLENQCKTETQESQAFQE<br>RDGRMVAGKVLMAKQEIVECVASAAMISPG<br>KLPGETHSQRIAEEALGGLDNSKKQKGNAA<br>GNKISQLPSQDRHFSLATFNRRIPTEHSVL<br>E ZSCAN30 JHU29179 { 0.990, 0.978, 0.973 }
59	MKFLVFLGIITTVAAFHQECSLQSCTQHQP<br>YVVDDPCPIHFYSKWYIRVGARKSAPLIEL<br>CVDEAGSKSPIQYIDIGNYTVSCLPFTINC<br>QEPKLGSLVVRCSFYEDFLEYHDVRVVLDF<br>I orf8	LTQHQRIHTGEKPYECKECGKAFRRSAAYL<br>QHQRLHTGEKLYKCKECWKAFGCRSLFIVH<br>QRIHTGEKPYQCKECGKAFTQKIASIQHQR<br>VHTGEKPYECKVCGKAFKWYGSFVQHQKLH<br>P ZNF621 JHU11225 { 0.983, 0.977, 0.974 }
60	MKFLVFLGIITTVAAFHQECSLQSCTQHQP	LIQHQRIHTGEKPYECKECGKAFSSSSVFL

	YYVDDPCPIHFYSKWYIRVGARKSAPLIELCVDEAGSKSPIQYIDIGNYTVSCLPFTINCQEPKLGSLVVRCSFYEDFLEYHDVRVVLDFI orf8	QHQRFHTGEKLYECNECWKTFSCSSSFTVHQRMHTGEKPYECKECGKRLSSNTALTQHQRIHTGEKPFECKECGKAFNQKITLIQHQRVHT ZNF620 JHU24458 { 0.979, 0.978, 0.976 }
61	MGYINVFAFPFTIYSLLLCRMNSRNYIAQVDVVNFNLT orf10	KMQDKTQMQEKAKEIYMTFLSSKASSQVNVEGQSRLNE RGS10 JHU11967 { 0.972 }
62	MGYINVFAFPFTIYSLLLCRMNSRNYIAQVDVVNFNLT orf10	DLNLVQALRQFLWSFRLPGEAQKIDRMMEAFAQRYCQC CYTH1 JHU05740 { 0.971 }
63	MGYINVFAFPFTIYSLLLCRMNSRNYIAQVDVVNFNLT orf10	EKGCHFLHILACARLSIRPGLSEAVLQQVLELLEDQSD OBFC1 JHU09466 { 0.988 }
64	MGYINVFAFPFTIYSLLLCRMNSRNYIAQVDVVNFNLT orf10	KVALQKALLYYESIHGRPVTKNERQVMKPLYDRYRLVK FAM13A JHU15982 { 0.980 }
65	MGYINVFAFPFTIYSLLLCRMNSRNYIAQVDVVNFNLT orf10	IKTITSSLKFYLRNLSEPVMTYRLHKELVSAAKSDNLD OPHN1 JHU13650 { 0.983 }
66	MGYINVFAFPFTIYSLLLCRMNSRNYIAQVDVVNFNLT orf10	QAGFTLHSAIYAARPDVKCVVHIHTPAGAAVSAMKCGL ADD1 JHU13212 { 0.980 }
67	MGYINVFAFPFTIYSLLLCRMNSRNYIAQVDVVNFNLT orf10	SPQPLLQDITYLRIPVADTPEVPIKKHFKECINFIHCC DUSP15 JHU12281 { 0.980 }
68	MGYINVFAFPFTIYSLLLCRMNSRNYIAQVDVVNFNLT orf10	NVAHGLAWSYYIGYLRLILPELQARIRTYNQHYNNLLR TMEM173 JHU05940 { 0.988 }
69	MGYINVFAFPFTIYSLLLCRMNSRNYIAQVDVVNFNLT orf10	KMQDKTQMQEKAKEIYMTFLSSKASSQVNVEGQSRLNE RGS10 JHU08427 { 0.973 }
70	MGYINVFAFPFTIYSLLLCRMNSRNYIAQVDVVNFNLT orf10	ESSSSMPLSFPSLLPSVPHNTNPSPPLMSYITSQEMKC C14orf119 JHU04523 { 0.960 }
